# Supplementary material for: Genome-Wide Identification, Molecular Characterization, and Expression Analysis of the HSP70 and HSP90 Gene Families in Thamnaconus septentrionalis
Source: Int J Mol Sci. 2024 May 24;25(11):5706. doi: 10.3390/ijms25115706 (PMC11172388; doi:10.3390/ijms25115706)
Supplement: Supplementary file 1 [file ijms-25-05706-s001.zip › Table S3-Protein sequences of all species used to construct phylogenetic trees.pdf]

HSP70 tree_all species protein sequences

>HSPA1A Homo sapiens

MAKAAAIGIDLGTTYSCVGVFQHGKVEIIANDQGNRTTPSYVAFTDTERLIGDAAKN
QVALNPQNTVFDA

KRLIGRKFGDPVVQSDMKHWPFQVINDGDKPKVQVSYKGETKAFYPEEISSMVLTK
MKEIAEAYLGYPVT

NAVITVPAYFNDSQRQATKDAGVIAGLNVLRIINEPTAAAIAYGLDRTGKGERNVLIFD
LGGGTFDVSIL

TIDDGIFEVKATAGDTHLGGEDFDNRLVNHFVEEFKRKHKKDISQNKRAVRRLRTACE
RAKRTLSSSTQA

SLEIDSLFEGIDFYTSITRARFEELCSDLFRSTLEPVEKALRDAKLDKAQIHDLVLVGGS
TRIPKVQKLL

QDFFNGRDLNKSINPDEAVAYGAAVQAAILMGDKSENVQDLLLLDVAPLSLGLETAG
GVMTALIKRNSTI

PTKQTQIFTTYSDNQPGVLIQVYEGERAMTKDNNLLGRFELSGIPPAPRGVPQIEVTFD
IDANGILNVTA

TDKSTGKANKITITNDKGRLSKEEIERMVQEAEKYKAEDEVQRERVSAKNALESYAF
NMKSAVEDEGLKG

KISEADKKKVLDKCQEVISWLDANTLAEKDEFEHKRKELEQVCNPIISGLYQGAGGP
GPGGFGAQGPKGG

SGSGPTIEEVD

>hspa1a Mus musculus

MAKNTAIGIDLGTTYSCVGVFQHGKVEIIANDQGNRTTPSYVAFTDTERLIGDAAKNQ
VALNPQNTVFDA

KRLIGRKFGDAVVQSDMKHWPFQVVNDGDKPKVQVNYKGESRSFFPEEISSMVLTK
MKEIAEAYLGHPVT

NAVITVPAYFNDSQRQATKDAGVIAGLNVLRIINEPTAAAIAYGLDRTGKGERNVLIFD
LGGGTFDVSIL

TIDDGIFEVKATAGDTHLGGEDFDNRLVSHFVEEFKRKHKKDISQNKRAVRRLRTACE
RAKRTLSSSTQA

SLEIDSLFEGIDFYTSITRARFEELCSDLFRGTLEPVEKALRDAKMDKAQIHDLVLVGG STRIPKVQKLL

QDFFNGRDLNKSINPDEAVAYGAAVQAAILMGDKSENVQDLLLLDVAPLSLGLETAG GVMTALIKRNSTI

PTKQTQTFTTYSDNQPGVLIQVYEGERAMTRDNNLLGRFELSGIPPAPRGVPQIEVTF DIDANGILNVTA

TDKSTGKANKITITNDKGRLSKEEIERMVQEAERYKAEDEVQRDRVAAKNALESYAF NMKSAVEDEGLKG

KLSEADKKKVLDKCQEVISWLDSNTLADKEEFVHKREELERVCSPIISGLYQGAGAPG AGGFGAQAPKGA

SGSGPTIEEVD

>HSPA1B Homo sapiens

MAKAAAIGIDLGTTYSCVGVFQHGKVEIIANDQGNRTTPSYVAFTDTERLIGDAAKN QVALNPQNTVFDA

KRLIGRKFGDPVVQSDMKHWPFQVINDGDKPKVQVSYKGETKAFYPEEISSMVLTK MKEIAEAYLGYPVT

NAVITVPAYFNDSQRQATKDAGVIAGLNVLRIINEPTAAAIAYGLDRTGKGERNVLIFD LGGGTFDVSIL

TIDDGIFEVKATAGDTHLGGEDFDNRLVNHFVEEFKRKHKKDISQNKRAVRRLRTACE RAKRTLSSSTQA

SLEIDSLFEGIDFYTSITRARFEELCSDLFRSTLEPVEKALRDAKLDKAQIHDLVLVGGS TRIPKVQKLL

QDFFNGRDLNKSINPDEAVAYGAAVQAAILMGDKSENVQDLLLLDVAPLSLGLETAG GVMTALIKRNSTI

PTKQTQIFTTYSDNQPGVLIQVYEGERAMTKDNNLLGRFELSGIPPAPRGVPQIEVTFD IDANGILNVTA

TDKSTGKANKITITNDKGRLSKEEIERMVQEAEKYKAEDEVQRERVSAKNALESYAF NMKSAVEDEGLKG

KISEADKKKVLDKCQEVISWLDANTLAEKDEFEHKRKELEQVCNPIISGLYQGAGGP GPGGFGAQGPKGG

SGSGPTIEEVD

>hspa1b Mus musculus

MAKNTAIGIDLGTTYSCVGVFQHGKVEIIANDQGNRTTPSYVAFTDTERLIGDAAKNQ VALNPQNTVFDA

KRLIGRKFGDAVVQSDMKHWPFQVVNDGDKPKVQVNYKGESRSFFPEEISSMVLTK MKEIAEAYLGHPVT

NAVITVPAYFNDSQRQATKDAGVIAGLNVLRIINEPTAAAIAYGLDRTGKGERNVLIFD LGGGTFDVSIL

TIDDGIFEVKATAGDTHLGGEDFDNRLVSHFVEEFKRKHKKDISQNKRAVRRLRTACE RAKRTLSSSTQA

SLEIDSLFEGIDFYTSITRARFEELCSDLFRGTLEPVEKALRDAKMDKAQIHDLVLVGG STRIPKVQKLL

QDFFNGRDLNKSINPDEAVAYGAAVQAAILMGDKSENVQDLLLLDVAPLSLGLETAG GVMTALIKRNSTI

PTKQTQTFTTYSDNQPGVLIQVYEGERAMTRDNNLLGRFELSGIPPAPRGVPQIEVTF DIDANGILNVTA

TDKSTGKANKITITNDKGRLSKEEIERMVQEAERYKAEDEVQRDRVAAKNALESYAF NMKSAVEDEGLKG

KLSEADKKKVLDKCQEVISWLDSNTLADKEEFVHKREELERVCSPIISGLYQGAGAPG AGGFGAQAPPKG

ASGSGPTIEEVD

>hspa1b Danio rerio

MAKGLSIGIDLGTTYSCVGVFQHGKVEIIANDQGNRTTPSYVAFTDTERLIGDAAKNQ VAMNPNNTIFDA

KRLIGRKFDDQVVQSDMKLWPFKVISEDGKPKVQVEYKGENKTFYPEEISSMVLVK MREIAEAYLGHRVN

NAVITVPAYFNDSQRQATKDAGVIAGLNVLRIINEPTAAAIAYGLDKGRKGERNVLIFD LGGGTFDVSIL

TIEDGIFEVKATAGDTHLGGEDFDNRMVKHFVEEFKRKHKKDISQNKRAVRRLRTAC ERAKRTLSSSSQA

SIEIDSLFEGIDFYTSITRARFEELNAELFRGTLDPVEKALRDAKMDKSQIQDIVLVGGS TRIPKIQKLL

QDFFNGRDLNKSINPDEAVAYGAAVQAAILMGDTSENVQDLLLLDVAPLSLGIETAGG
VMTALIKRNTTI

PTKQTQIFSTYSDNQPGVLIQVFEGERAMTKDNNLLGKFDLTGIPPAPRGVPQIEVTFD
IDANGILNVSA

ADKSTGKENKITITNDKGRLSKEDIERMVQDAEKYKAEDEVQREKIAAKNALESYAF
SMKNTVEDENLRG

KISEQDKKKIIDKCTEVVSWLENNQLAEKEEYEHHQKELESVCNPIISRLYQGGAPPA
GGCGFQAQPESS

QGPTIEEVD

>hspa1b Oryzias latipes

MDTAKGVSIGIDLGTTYSCVGVFQHGKVEIIANDQGNRTTPSYVAFTDAERLIGDAAK
NQVAMNPSNTVF

DAKRLIGRNFNDPIVQSDMKLWPFKVINDNGKPKIQVDYKGETKAFYPEEISSMVLV
KMKEIAEAYLGQR

VSNVVITVPAYFNDSQRQATKDAGVIAGLNVMRIINEPTAAAIAYGLDKGKRGEQNVL
IFDLGGGTFDVS

ILTIEDGIFEVKATSGDTHLGGEDFDNRMVNHFVEEFKRKHKKDISQNKRAVRRLRTA
CERAKRTLSSSS

QASIEIDSLFDGIDFYTSITRARFEELNSDLFRGTLEPVEKALRDAKLDKSKIHEIVLVG
GSTRIPKIQK

LLQVLFNGRELNKSINPDEAVAYGAAVQAAILMGDTSENVQDLLLLDVTPLSLGIETA
GGVMTPLIKRNT

TIPTKQTQIFSTYSDNQPGVLIQVYEGERAMTKDNNLLGKFELTGIPPAPRGVPQVEVT
FDIDANGILNV

SAVDKSTGKENKITITNDKGRLSKEDIERMVQDAEKYKAEDELQREKVAAKNALESY
AYNMKSSVEDENI

VGKISEEDKKVVVDKCNQTISWLENNQLAEKDEYEHQQKELEKVCRPIVTKLYQGA
APSGSFSDQTGGSS

QGPTIEEVD

>hspa1b Gasterosteus aculeatus

MSSAKGVSIGIDLGTTYSCVGVFQHGKVEIIANDQGNRTTPSYVAFTDTERLIGDAAK

NQVAMNPTNTIF

DAKRLIGRKFNDAVVQSDMKLWPFKVISDNGKPKVQVDYKGETKAFYPEEISSMVLV KMKEIAEAYLGQG

VSNAVITVPAYFNDSQRQATKDAGVISGLNVLRIINEPTAAAIAYGLDKGKRGERNVLI FDLGGGTFDVS

ILTIEDGIFEVKATAGDTHLGGEDFDNRMVNHFVEEFKRKHKKDISGNKRAVRRLRTA CERAKRTLSSST

QASIEIDSLFEGIDFYTSITRARFEELNSELFRGTLEPVEKALQDAKLDKSKIHEIVLVG GSTRIPKIQK

LLQDFFNGRELNKSINPDEAVAYGAAVQAAILMGDTSENVQDLLLLDVAPLSLGIETA GGVMTPLIKRNT

TIPSKQTQIFSTYSDNQPGVLIQVYEGERAMTKDNNLLGKFDLTGIPPAPRGVPQVEVT FDVDANGILNV

SAVDKSTGKENKITITNDKGRLSKEEIERMVQDSEKYKAEDDVQREKIAAKNSLESYA YHMKSSVEDENL

KGKISEEDKKKVVDKCNQTISWLENNQLAEKDEYEHQLSELEKVCKPVVTTLYQGA APSGSCGSQAGGSS

RGPTIEEVD

>HSPA1L Homo sapiens

MATAKGIAIGIDLGTTYSCVGVFQHGKVEIIANDQGNRTTPSYVAFTDTERLIGDAAK NQVAMNPQNTVF

DAKRLIGRKFNDPVVQADMKLWPFQVINEGGKPKVLVSYKGENKAFYPEEISSMVLT KLKETAEAFLGHP

VTNAVITVPAYFNDSQRQATKDAGVIAGLNVLRIINEPTAAAIAYGLDKGGQGERHVLI FDLGGGTFDVS

ILTIDDGIFEVKATAGDTHLGGEDFDNRLVSHFVEEFKRKHKKDISQNKRAVRRLRTAC ERAKRTLSSST

QANLEIDSLYEGIDFYTSITRARFEELCADLFRGTLEPVEKALRDAKMDKAKIHDIVLV GGSTRIPKVQR

LLQDYFNGRDLNKSINPDEAVAYGAAVQAAILMGDKSEKVQDLLLLDVAPLSLGLET AGGVMTALIKRNS

TIPTKQTQIFTTYSDNQPGVLIQVYEGERAMTKDNNLLGRFDLTGIPPAPRGVPQIEVT FDIDANGILNV

TATDKSTGKVNKITITNDKGRLSKEEIERMVLDAEKYKAEDEVQREKIAAKNALESYA FNMKSVVSDEGL

KGKISESDKNKILDKCNELLSWLEVNQLAEKDEFDHKRKELEQMCNPIITKLYQGGC TGPACGTGYVPGR

PATGPTIEEVD

>hspa1l Mus musculus

MAANKGMAIGIDLGTTYSCVGVFQHGKVEIIANDQGNRTTPSYVAFTDTERLIGDAA KNQVAMNPQNTVF

DAKRLIGRKFNDPVVQSDMKLWPFQVINEAGKPKVMVSYKGEKKAFYPEEISSMVLT KMKETAEAFLGHN

VTNAVITVPAYFNDSQRQATKDAGVIAGLNVLRIINEPTAAAIAYGLDKGSHGERHVLI FDLGGGTFDVS

ILTIDDGIFEVKATAGDTHLGGEDFDNRLVSHFVEEFKRKHKKDISQNKRAVRRLRTAC ERAKRTLSSST

QANLEIDSLYEGIDFYTSITRARFEELCADLFRGTLEPVEKSLRDAKMDKAKIHDIVLV GGSTRIPKVQK

LLQDYFNGRDLNKSINPDEAVAYGAAVQAAILMGDKSEKVQDLLLLDVAPLSLGLET AGGVMTVLIKRNS

TIPTKQTQIFTTYSDNQPGVLIQVYEGERAMTRDNNLLGRFDLTGIPPAPRGVPQIEVT FDIDANGILNV

TAMDKSTGKANKITITNDKGRLSKEEIERMVQEAERYKAEDEGQREKIAAKNALESY AFNMKSAVGDEGL

KDKISESDKKKILDKCNEVLSWLEANQLAEKDEFDHKRKELENMCNPIITKLYQSGCT GPTCTPGYTPGR

AATGPTIEEVD

>HSPA4 Homo sapiens

MSVVGIDLGFQSCYVAVARAGGIETIANEYSDRCTPACISFGPKNRSIGAAAKSQVISN AKNTVQGFKRF

HGRAFSDPFVEAEKSNLAYDIVQLPTGLTGIKVTYMEEERNFTTEQVTAMLLSKLKET

AESVLKKPVVDC

VVSVPCFYTDAERRSVMDATQIAGLNCLRLMNETTAVALAYGIYKQDLPALEEKPRN
VVFVDMGHSAYQV

SVCAFNRGKLKVLATAFDTTLGGRKFDEVLVNHFCEEFGKKYKLDIKSKIRALLRLSQ
ECEKLKKLMSAN

ASDLPLSIECFMNDVDVSGTMNRGKFLEMCNDLLARVEPPLRSVLEQTKLKKEDIYA
VEIVGGATRIPAV

KEKISKFFGKELSTTLNADEAVTRGCALQCAILSPAFKVREFSITDVVPYPISLRWNSPA
EEGSSDCEVF

SKNHAAPFSKVLTFYRKEPFTLEAYYSSPQDLPYPDPAIAQFSVQKVTPQSDGSSSKVK
VKVRVNVHGIF

SVSSASLVEVHKSEENEEPMETDQNAKEEEKMQVDQEEPHVEEQQQQTPAENKAESE
EMETSQAGSKDKK

MDQPPQAKKAKVKTSTVDLPIENQLLWQIDREMLNLYIENEGKMIMQDKLEKERND
AKNAVEEYVYEMRD

KLSGEYEKFVSEDDRNSFTLKLEDTENWLYEDGEDQPKQVYVDKLAELKNLGQPIKI
RFQESEERPKLFE

ELGKQIQQYMKIISSFKNKEDQYDHLDAADMTKVEKSTNEAMEWMNNKLNLQNKQ
SLTMDPVVKSKEIEA

KIKELTSTCSPIISKPKPKVEPPKEEQKNAEQNGPVDGQGDNPGPQAAEQGTDTAVPSD
SDKKLPEMDID

>hspa4 Mus musculus

MSVVGIDLGFQSCYVAVARAGGIETIANEYSDRCTPACVSFGPKNRSIGAAAKSQVISN
AKNTVQGFKRF

HGRAFSDPFVEAEKSNLAYDIVQLPTGLTGIKVTYMEEERNFTTEQVTAMLLSKLKET
AESVLKKPVVDC

VVSVPSFYTDAERRSVMDATQIAGLNCLRLMNETTAVALAYGIYKQDLPALEEKPRN
VVFVDMGHSAYQV

SVCAFNKGKLKVLATAFDTTLGGRKFDEVLVNHFCEEFGKKYKLDIKSKIRALLRLSQ
ECEKLKKLMSAN

ASDLPLSIECFMNDIDVSGTMNRGKFLEMCDDLLARVEPPLRSVLEQSKLKKEDIYAV

EIVGGATRIPAV

KEKISKFFGKELSTTLNADEAVTRGCALQCAILSPAFKVREFSITDVVPYPISLRWNSPA
EEGLSDCEVF

PKNHAAPFSKVLTFYRKEPFTLEAYYSSPQDLPYPDPAIAQFSVQKVTPQSDGSSSKVK
VKVRVNVHGIF

SVSSAALVEVHKSEESEEPMETDQNAKEEEKMQVDQEEPHTEEQQQQPQTPAENKAE
SEEMETSQAGSKD

KKTDQPPQAKKAKVKTSTVDLPIESQLLWQLDREMLGLYTENEGKMIMQDKLEKER
NDAKNAVEEYVYEM

RDKLSGEYEKFVSEDDRNTFTLKLEDTENWLYEDGEDQPKQVYVDKLAELKSLGQPI
KTRFQESEERPKL

FEELGKQIQQYMKVISSFKNKEDQYEHLDAADVTKVEKSTNEAMEWMNSKLNLQN
KQSLTVDPVVKTKEI

EAKIKELTSICSPIISKPKPKVEPPKEEPKHAEQNGPVDGQGDNPGSQAAEHGADTAVP
SDGDKKLPEMD

ID

>hspa4 Gallus gallus

MSVVGIDLGFQSCYVAVARAGGIETIANEYSDRSTPSCIAFGPKNRSIGAAAKSQVISN
AKNTVQSFKRF

HGRAFSDPFVQAEKTSLAYELVQLPTGSTGIKVMYMEEERNFTIEQMTGMLLTKLKE
TAENALKKPVVDC

VVSVPCFYTDAERRSVMDATQIAGLNCLRLINESTAVALAYGIYKQDLPALEEKPRNV
VFVDMGHSAYQV

SICAFNKGKLKVLATAFDTTLGGRKFDEMLVEYFCEEFGKKYKLDIKSKIRALLRLYQ
ECEKLKKLMSAN

ASDLPMNIECFMNDIDVSGTMNRSKFLEMCDGLLARVEAPLRSVLEQAKLKKEDIYA
VEIVGGTTRIPAV

KEKISKFFGKEVSTTLNADEAVARGCALQCAILSPAFKVREFSITDLIPYPISLRWNSPA
EEGLSDCEVF

PKNHAAPFSKVLTFYRKEPFTLEAYYSSPKELPYPDPAIAHFLVQKVTPQTDGSSSKVK
VKVRVNIHGIF

SVSSASLVEVHKSDENEEPMETDQHAKEEEKMQVDQEEQQKTEEQQQAQAENKAES
EEMETSQGDSKDKK

VDQPPQAKKAKVKTTTVDLPIENQLVWQIGKDMLNLFIENEGKMIMQDKLEKERND
AKNAVEEYVYDMRD

KLCSIYEKFVSEDDRNSFTLKLEDTENWLYEDGEDQPKQIYIDKLTELKALGQPIQARF
QESEERPKAFE

DLGKQIQQYMKTVHAFKAKDEQYDHLDEADVAKVEKSANEAMEWMNNKLNLQN
KRSLTLDPVIKAKDIQA

KTKELTSICNPIVTKPKPKVELPKEEQKPTEPNGPVEGQGDGSSGSQTADPSTAPAPAA
AEKKLPEMDID

>hspa4a Danio rerio

MSVVGFDVGFQSCYVAVARAGGIETVANEYSDRCTPSFVSFGPRNRSIGAAAKSQVVT
NCKNTVQGFKRF

HGRAFSDPYVETTQSSLVYDLAQMPNGTTGIKVMYMEEEKLFGIEQVTAMLLTKLKE
TAESALKKPVADC

VISVPSYFTDAERRSVMDAAQIAGLNCLRLMNDTTAVALAYGIYKQDLPAPEEKPRTV
VFVDVGHAGYQV

SACAFNKGKLKVLGSAFDPELGGKDFDEVLVKHFCEEFAQKYKLDVRSKPRALVRLY
QECEKLKKLMSAN

SSDLPLNIECFMNDIDVSSKLNRAKFEELCAGLLAKVEAPLQSIMEQTRLKKEDIYAVE
IIGGASRIPAI

KERISKFFGKELSTTLNLDEAVARGCALQCAILSPAFKVREFSITDVVPYPISLKWTSAA
DEGVSDCEVF

PKNHAAPFSKVLTFYRKEPFTLEAYYNNPKALPYPDPTIGQFTIHKVVPQASGESSKV
KVKVRVNVHGVF

SVSSASLVELLKPGEGEEPMETDTPAKDEENKMQVDQEAQKAQADDQKEQADKKSD
TEDMETSPEDKQEK

KNDQPPQAKKAKVKTKTVDLPIMNSLQWQLASDALNLFMENEGKMIMQDKLEKER
NDAKNYVEEYVYEMR

DKLHGVLENFVSEAERDSFSLKLEDTENWLYEEGEDQQKQVYIDKLAELKKLGDPIQ
SRYIEAEVRPKAF

EELGRQIQLYMKVVEAFKAKDELYDHLDELEMVKVEKQVNDAMTWMNNKMNLQS KQSLSQDPAVKVQEIQ

TKTKELYSACNPVVTKSKPKVEPPKEETAAEQNGPVNGQQPSDAQPEKSPENTEAKL PEMDID

>hspa4b Danio rerio

MSVVGFDVGFLNCYVAVARAGGIETVANEYSDRCTPACVSFGPRNRSIGAAAKSQMV TNCKNTVHGFKRF

HGRAFSDPFVQNLKPSLVYDLAQMPSGTTGLKVMYMEEEKVFSIEQVTAMLLTKLKE TAESALKKPVADC

VISVPCFYTDAERRSVIDAAQIAGLNCLRLMNETTAVALAYGIYKQDLPAPEEKPRNV VFVDIGHSGYQV

SVCAFNKGKLKILATAFDPEMGGKYFDERLVKYFCEEFVVKYKLDAKTKPRALIRLF QECEKLKKLMSAN

SSDLPLNIECFMNDVDVSSRLNRAQFEEMCADILARVEPPLRSLLEQAHLKKDDIHAV EIVGGASRMPAI

KERISKFFGKEPSTTLNADEAVARGCALQCAILSPAFKVREFSITEVVPFPISLKWNSAA EDGVSDCEVF

PKNHAAPFSKVLTFYRREPFTLDAYYNSPKELPYPDPTIGQYVVQKVVPQASGESSKV KVKVRVNIHGIF

SVSSASLVEVQKSEEEEESMETEQSTEKENENKMQVDQEEQKTPETEQENGEKKPGT EEMETSAEEGKQE

KKSDQPPQAKKPKVKTKVLDLPIENNPQWQLANDMLNLFVESEGKMIMQDKLEKER NDAKNYVEEYVYEM

RDKLHGIFEKFVTESDRDVLSLKLEDTEVWLYEDGEDQPKQIYIDKLAELKNLGQPIQ DRYREFEERPKA

FEELGRQLQQYMKIVEAYKTKEEQYDHLEEAEIQKVDKMVNDVMIWMNSKMNQQS KQSLAIEPVVKTTEI

QAKTRELFSTCNPVVTKPKPKVDLPKEENPSEPNGPVNTQENPEAQPGGTEPAAADS AGNTENKPDMDLD

>hspa4a Oryzias latipes

MSVVGFDLGYQSCYVAVARAGGIETVANEYSDRCTPSFVSFGPRNRSIGASAKSQAVT

NY

KNTVQGFKRFHGRAFSDPYVQSAKSNLVYDVAQMPSGSTGLKVMYMEEEKVYGVE
QVTAM

LLTKLKETAESALKKQVIDCVISVPSYFTDAERRSVMDAAQIAGLNCLRLMNDTTAVT
LA

YGIYKQDLPAPEEKPRTVVFVDLGHSGYQVSVCAFNKGKLKILATAFDSDLGGKDFD
DIL

ANHFCEEFAKKYKLDVRSKPRALVRLYQECEKLKKLMSANSSDLPLNIECFMNDIDV
SSK

LNRGQFEEMCAGLLAKVEGPLRSVMEQTKLKKEDIYAVEIVGGASRIPAIKERIGKFF
GK

ELSTTLNADEAVARGCALQCAILSPAFKVREFSITDVVPFSISLKWNSAADDGLSDCEV
F

PKNHAAPFSKVLTFYRSEPFTLEAYYNNAKELPCPDTNIGQFLIQNVVPQATGESAKV
KV

KVRVNIHGVFSVSSASLVEVVKAVEGEEPMETDQIVKEDEVCVCEQQHKGKMIMQD
KLEK

ERNDAKNNVEEYVYEMRDKLHGVLEKFVNEAERDAFSLKLEDTENWLYEEGEDQQ
KQVYI

DKLAELKKIGQPIYSRYMEAEERPKAFEELGRRIQMYMKIVEAYKAKEEQYDHLDEM
EVT

QVDKQVSEAMIWMNSKLNQQKSHDLALDPVVKVGEIQAKAKELYSSCNPVVSKPKP
KVEP

PKEEKTENGPVNGLSPETAETEPSSPTKATPAGSKQETTESKLPEMDID

>hspa4b Oryzias latipes

MSVIGFDVGFLNCYVAVARAGGIETVANEYSDRCTPACVSFGPRNRSIGAAAKSQVVT
NC

KNTVQGFKRFHGRVFSDPYVQSIKNSLVYDIAQMPTGTTGIKVRAETFSVSTMIIISKL
F

WTNCVVSSVPCYYTDAERRSVVDAAQIAGLNCLRLMNETTAVALAYGIYKQDLPAPE
EKA

RNVVFVDLGHSGYQTSVCAFNKGKLKVLATACDPQLGGKDFDEMLVQHFCEDFGKR
YKLD

VKTKPRALVRLYQECEKLKKLMSANSSDLPLNIECFMNDIDVTGKMNRGQFEEMCA
DILT

RVEPPLQSLLEQAKLKKEDIYAVEIVGGASRIPAVKERISKFFGKELSTTLNADEAVARG

CALQCAILSPAFKVREFSITEVVPYPISLKWHSAAEDGLSDCEVFPKNHAAPFSKVLTF
Y

RREPFSLEAYYSCPSELPYPDPTIGQFLIQKVVPQASGESSKVKVKVRVNIHGIFSVSSA

SLVEVQKSDETEEPMETEQAATDKDGEVPQSTSTEENKGEKKSDQPPQAKKAKVKTK
VLE

LPIENSPQWQLANDMLNLFVENEGKMIMQDKLEKERNDAKNYVEEYVYDMRDKLH
GIFEK

FVSESVNALSLKLEDTENWLYEDGEDQPKQVYIDKLAELKKLGQPIQERYLEAEERP
KAF

DEMGKQIQQYMKFVEAFKMNEEQYNHLDEADVSKVDKLTSDAMIWMNSTMNQQS
KQSLTL

DPAVKVKDIRAKTRELFSACNPIVTKPKPRVEAPKEDTPAEQNGPVNGQEKPQEEPAD
KG

AAENTGNPASESADTKPDMDLD

>hspa4a Gasterosteus aculeatus

MSVVGFDLGFQSCYVAVARAGGIETVANEYSDRCTPSVISFGPRNRSIGAAAKSQVVT
NC

RNTVQAFKRFHSRAFSDPYVQSAKSNLVYDLAEMPSGATGIKVMYMEEERVFSIEQV
TGM

LLSKLKETAESALKKPVADCVISVPSYFTDSDRRSVMDAAQIAGLNCLRLMNETTAVS
LA

YGIYKQDLPAPEEKPRNVVFVDVGHSGYQVSVCAFNKGKLKILATAFDAELGGKALD
EIL

VNHFCEEFGKKYKLDVKSKPRALVRLHQECEKLKKLMSANSSDLPLNIECFMNDIDV
SGK

LNRGQFEEMCAGHLAKVEGPLRSVMEQAKLKKEDIYAVEIVGGASRIPSIKERISRFFG

K

ELSTTLNADEAVARGCALQCAILSPAFKVREFSITDVVPYSISLKWNSAAEEGLSDCEV
F

QKNHAAPFSKVLTFFRKEPFTLEGYYSNPKELPYPNSHIGPFLIHNLVPHASGESSKVK
V

KVRVNVHGMFSVSSASLIEVLKTAEGEEQLETDHPAKEEENKMHVDQDDPKLQTGD
NGEN

KSEVEEMETTEDSKQAKKNDQPPQAKKPKVKTKTVELPVEHKLLGQLSDDVLNMSV
ENEG

QMIMQDKLEKERNDAKNNVEEYVYDMRDKLHGVLEKFVNEAHRDAFSLKLEDTET
WLYED

GEDQQKQVYIDKLAELKEMGQPIFERYMEAEERPRAFQELGRLIQMYMKIIDAYKAK
DEL

YDHMDELEVTSVEKQVKAAMIWMNGKMNQQNNQDLTLEPVVKVAEIQAKIKELYS
ACNPV

VSKPKPKAELPQEEKTENGPVNGQEGQEGTEGTDSQPGNSDKATPAGAEQGAAEKK
LPDM

DID

>hspa4b Gasterosteus aculeatus

MSVVGFDVGFLNCYVAVARAGGIETVANEYSDRCTPACVSFGPRNRAIGAAAKSQIVT
NC

KNTVQGFKRLHGRAFTDPYVQRLKNSLVYDIVQMPTGTTGIKVNYMEEEKVFSVEQ
VTAM

LLTKLKETAEHALKKPVADCVVSVPCYYTDAERRSVVDAAQIAGLNCLRLMNETTAV
ALA

YGIYKQDLPAPEEKPRNVVFVDLGHSGYQTSVCAFNKGKVKVLSTACDPELGGKDF
DEML

VRYFCEDFSKKYKIDVKSRPRALIRVYQECEKLKRVMSANSSDLPFNIECLMNDIDVS
GK

MNRGQFEEMCADILARIEAPLQNLLEHAKLKREDIYAVEIVGGASRIPAVKERISKFFG
K

ELNTTLNADEAVARGCALQCAILSPAFKVREFSITDVVTYPISLKWPSAAEEGLSDCEV
F

PMNHAAPFSKVLTFYRKEPFSLEAYYNKPSELCYPDPTIGQFLIQKVIPQASGESSKVK
V

KVRVNIHGIFSVSSASLVEVQKSDETEEPMETEHANEKEGESKMQTDQDEQQGQADG
SKE

AEEKTPRENEEMDTTTEEGKGEKKSDQPPQAKKPKVKTKVVELPIENSPQWQLADD
MLNL

FVENEGKMIMQDKLEKERNDAKNYVEEYVYEMRDKLHGVLEKFVSESDRDALSLK
LEDTE

NWLYEDGEDQPKQVYIDKLTELKKLGQPIKERYTEAEMRPKAFEELGKQIQQYMKFV
EAF

KMKDEQYDHLDEADVKKVDKLTSDAMMWMNSSMNQQSKQNLTVDPSVKVKDIEA
KTRELF

SACNSIVTKPKPRVELPKEDNPAEQNGPVNGQEKAPEEAASKAATENPGSENKPDMD
LD

>hspa4 Larimichthys crocea

MSVVGFDVGFMSCYVAVARAGGIETVANEYSDRSTPACVSFGPRNRSIGAAAKSQVV
TNCKNTVQGFKRF

HGRAFSDPYVQRLKNSLVYDIAQMPTGTTGIKVMYMEEEKVFSIEQVTAMLLTKLKE
TAENALKKPVADC

VVSVPCYYTDAERRSVVDAAQIAGLNCLRLMNETTAVALAYGIYKQDLPAPEEKARN
VVFVDLGHSGFQT

SVCAFNKGKLKILSTACDPELGGKDFDEVLVKHFCEEFGKKYKLDVKTKPRALVRLY
QECEKLKKLMSAN

SSDLPLNIECFMNDIDVSAKLNRGAFEEMCADILARVEAPLQSLLEQTKLKKEDIYAVE
IVGGASRIPAI

KERISKFFGKELSTTLNADEAVARGCALQCAILSPAFKVREFSITDVAPYPISLKWHSAA
EEGLSDCEVF

PRNHAAPFSKVLTFYRKEPFSLEAYYNCPNELPYPDPTIGQFMIQKVVPQASGESSKV
KVKVRVNIHGIF

SVSSASLVEVQKSDETEEPMETEQANDKDGESKMQTDQDEQGQGDGQKETEEKTQRENEEMETTTEEGKG

EKKSDQPPSAKKPKVKTKVLDLPIENSPQWQLADDMLNLFVENEGKMIMQDKLEKERNDAKNNVEEYVYD

MRDKLHGMLEKFVSESDRDALSLKLEDTENWLYEDGEDQPKQVVYIDKLAELKKLGQPIQERYTEAEDRPK

AFDEMGKQIQLYMKFVEAYKMKEEQYDHLDEADVTKVDKMASDVMIWMNSAMNQQSKLSLSVDPSIKVKD

IQAKTRELFSACNPIVTKPKPKVELPKEDTPAEQNGPVNGQEKPQEETADKGTTENTGNPTSETTENKPD

MDLD

>hspa4 Oreochromis niloticus

MSVVGFDVGFLNCYVAVARAGGIETVANEYSDRCTPACVSFGPRNRSIGAAAKSQVVTNCKNTVQGFKKF

HGRAFSDPYVQSLKENMVYDIAQMPTGTTGIKVMYMEEEKVFSIEQVTAMLLTKLKETAENALKKPVADC

VVSVPCYYTDAERRSVVDAAQIAGLNCLRLMNETTAVALAYGIYKQDLPAPEEKARNVVFVDLGHSGYQT

SVCAFNKGKLKVLATACDPLLGGKDFDEVLVKHFCEEFGKKYKLDVKSKPRALVRLYQECEKLKKLMSAN

SSDLPLNIECFMNDIDVTGKMNRAQFEEMCADIWARVEPPLQSLLEQAKLKKEDIYAVEIVGGASRIPAV

KERISKFFGKELSTTLNADEAVARGCALQCAILSPAFKVREFSITDAVPYPISLKWHSAAEDGVSDCEVF

PKNHAAPFSKVLTFYRKEPFSLEAYYSSPNELPYPDPTIGQFMIQKVVPQASGESSKVKVKVRVNIHGIF

SVSSASLVEVQKSDEAEEPMETEQAADKDGESKMQTDQDEQPAQGDAQKETEEKTPRESEEMETSTEENK

GEKKSDQPPQAKKPKVKTKVLELPIENNPQWQLANDMLNLFVENEGKMIMQDKLEKERNDAKNNVEEYVY

EMRDKLHGMLEKFVSESDRDVLSLKLEDTENWLYEDGEDQPKQVVYIDKLAELKKLG

QPIQDRYAESEERP

KAFEELGKQIQQYMKFIEAYKMKDEQYDHLDEADVNKVDKLNNDAMIWMNSAMN QQSKQSLAVDPSVKVK

DIKAKTRELFGACNPIVTKPKPKVELPKEETPAEQNGPVDGQEKPQEAAADKGTTEA AGNPASETTESKP

DMDLD

>HSPA4L Homo sapiens

MSVVGIDLGFLNCYIAVARSGGIETIANEYSDRCTPACISLGSRTRAIGNAAKSQIVTNV RNTIHGFKKL

HGRSFDDPIVQTERIRLPYELQKMPNGSAGVKVRYLEEERPFAIEQVTGMLLAKLKET SENALKKPVADC

VISIPSFFTDAERRSVMAAAQVAGLNCLRLMNETTAVALAYGIYKQDLPPLDEKPRNV VFIDMGHSAYQV

LVCAFNKGKLKVLATTFDPYLGGRNFDEALVDYFCDEFKTKYKINVKENSRALLRLY QECEKLKKLMSAN

ASDLPLNIECFMNDLDVSSKMNRAQFEQLCASLLARVEPPLKAVMEQANLQREDISSI EIVGGATRIPAV

KEQITKFFLKDISTTLNADEAVARGCALQCAILSPAFKVREFSITDLVPYSITLRWKTSF EDGSGECEVF

CKNHPAPFSKVITFHKKEPFELEAFYTNLHEVPYPDARIGSFTIQNVFPQSDGDSSKVK VKVRVNIHGIF

SVASASVIEKQNLEGDHSDAPMETETSFKNENKDNMDKMQVDQEEGHQKCHAEHTP EEEIDHTGAKTKSA

VSDKQDRLNQTLKKGKVKSIDLPIQSSLCRQLGQDLLNSYIENEGKMIMQDKLEKER NDAKNAVEEYVYD

FRDRLGTVYEKFITPEDLSKLSAVLEDTENWLYEDGEDQPKQVYVDKLQELKKYGQP IQMKYMEHEERPK

ALNDLGKKIQLVMKVIEAYRNKDERYDHLDPTEMEKVEKCISDAMSWLNSKMNAQ NKLSLTQDPVVKVSE

IVAKSKELDNFCNPIIYKPKPKAEVPEDKPKANSEHNGPMDGQSGTETKSDSTKDSSQ HTKSSGEMEVD

>hspa4l Mus musculus

MSVVGIDLGFLNCYIAVARSGGIETIANEYSDRCTPACISLGSRTRAIGNAAKSQIVTNV
RNTIHGFKKL

HGRSFDDPIVQTERIRLPYELQKMPNGSTGVKVRYLEEERPFAIEQVTGMLLAKLKET
SENALKKPVADC

VISIPSFFTDAERRSVMAAAQVAGLNCLRLMNETTAVALAYGIYKQDLPSLDEKPRNV
VFIDMGHSAYQV

SVCAFNKGKLKVLATTFDPYLGGRNFDEALVDYFCDEFKTKYKINVKENSRALLRLY
QECEKLKKLMSAN

ASDLPLNIECFMNDLDVSSKMNRAQFEQLCASLLARVEPPLKSVMDQANLQREDINSI
EIVGGATRIPAV

KEQVTRFFLKDISTTLNADEAVARGCALQCAILSPAFKVREFSITDLVPYSVTLRWKTS
FEEGTGECEVF

SKNHPAPFSKVITFHKKEPFELEAFYTNLHEVPYPDPRIGNFTIQNVFPQSDGDSSKVK
VKVRINIHGIF

SVASASVIEKQNLEGDHNDAAMETEAPKSEGKEDVDKMQVDQEEGGHQKCHAEHT
PEEEIDHTGAKAKAP

PSDKQDRINQTIKKGKIKSIDLPIQSSLYRQLTQDLLNSYIENEGKMIMQDKLEKERND
AKNAVEEYVYD

FRDKLGTVYEKFITPEDMNKLSAMLEDTENWLYEEGEDQPKQVYVDRLQELKKYGQ
PIQMKYVEHEERPK

ALNDLGKKIQLVLKVIEAHRNKDERYDHLDPAEMERVEKYISDSMNWLNSKMNAQN
KLSLTQDPVVKVSE

IVTKSKELDNFCNPIVYKPKPKVEAPEDKAKTGSEHNGPMDGQSGSETSPDPPKGSSQ
HTDSGEMEVD

>hspa4l Gallus gallus

MSVVGIDLGFLNCYIGVARSGGIETIANEYSDRCTPACISLGSKTRAIGNAAKSQIVTN
VKNTLHGFKKL

HGRAFEDSYIQAERAKLPYELQKMPNGSVGVKVRYLDEERLFAVEQITGMLLAKLKE
TSESALKKPVADC

VISVPSFFTDAERRSVMAAAQIAGLNCLKLMNETTAVALAYGIYKQDLPALEEKPRNV

VFVDMGHSAYQV

SICAFNKGKLKVLATTFDPFLGGRNFDEALVDYFSEEFRTKYKLNVKENPRALLRLYQ
ECEKLKKLMSAN

ASDLPLNIECFMNDLDVSSKMNRAQFEQLCAALLSRVEPPLRAAMEQAKLQREDIYSI
EIVGGATRIPAV

KEQISSFFCKEISTTLNADEAVARGCALQCAILSPAFKVREFSITDVVPYSITLRWKSSY
EEGTGECEVF

SKNHAAPFSKVITFHKKEPFDLEAFYTHPHEVPYPDSRIGRFTIQNVGPQHDGDNSKV
KVKVRVNIHGLF

SVANASIIEKQNIDGDHNDAAMDTESSSKNQGREDELDKMQVDQDEGVQKSQAEQQ
SQADEEAENTGIET

KASSGDKQDHPTLPRAKTKVKSIDLPIQASLYRQLGQDLINCYIENEGKMMMQDKLE
KERNDAKNAVEEY

VYDFRDKLCGVFEKFITEEDTNKLTLMLEDTENWLYEDGEDQPKQVYMDKLQELRK
FGQPIQERYMEHEE

RPKVLNELGKKIQLLMKAVEAYKNKDEKYDHLDPAEMEKVEKYISEAMNWLNTKM
NAQNKLSLTQDPVVK

VAEIISKSKELDSFCNPIIYKPKPKIEPPNDGQSKANGEHNGPVNGQSSTETGPDPAKDN
SQQTKPSGEM

EVD

>hspa4l Danio rerio

MSVVGIDVGFQNCYIAVARSGGIETIANEYSDRCTPACISLASKNRTIGNAAKSQIITNF
KNTVHGFKKF

HGRAFDDPFVQGEKSRLPYSLHKLDNGNAGIKVRYLNEDKVFTIEQVTAMLLTKLKE
TSEHALKKPVVDC

VISVPSFFTDVERRSVMDATQIAGLNCLRLINDTTAVALAYGIYKQDLPNPEEKPRNVV
FVDIGHSSYQV

AIASFNKGKLKMLATAFDPYLGGRNFDEILVEYFCEDFKNRFKLNVKDNPRALLRLY
QECEKLKKLMSAN

SSDLPLNIECFMNDIDVHGKLNRTQFEEMCSQLMMRVEAPLRSVMEQSKLSRDEIYAI
EVVGGATRMPAI

KERISKFFGKDTSTTLNADEAVARGSALQCAILSPAFKVREFSITDTVPFPITLRWKSPT DESVGECEVY

SKNHPAPFSKIITFHKKEPFDLEAFYSCPHDLPYPDVRIGRFSVQNVVPQPDGDSSKVK VKVRVNVHGIF

SVSSASLIEKQKGEPEDVQMDTEPTVQNEGRPEEQSKMQVDQEGQGEQPSEEERANN SGIKDGDKQDQGA

SSSKAKSKVKSVDLPILANTTRQLDRDVLTHFVEYEKKMIIQDKLEKERNDAKNGVE EYVYDLRDKLCGI

YEKYVTEDESNRLTIMLEDTENWLYEEGEDQDKEIYQHKLAELKKYGEPIEERYREH EGRPRAFDELGKK

LQLFMKVVDMYRDKDERYEHLSAEDMGVVEKSVNEALGWMNTKMNAQSKLSFAQ DPAVKVADIIQKIQEL

EDVCNPILNRPKPKTEELVEEGDKSDGAHNGPTAQQGADSKGNQQTKPPSGEMDVD

>hspa4l Oryzias latipes

MSVVGIDVGFQNCYIAVARSGGIETIANEYSDRCTPACVSFASKNRMIGNAAKSQMIT NFKNTVHGFKKL

HGRAFDDAFILAEQPKLPYSLHKLPNGNAGIKVRYLDADKVFTVEQITGMLLSKLRE TSQSALKKPVVDC

VISVPSFFTDAERRSVFDATQIAGLNCLRLINDTTAVALAYGIYKQDLPNPEEKPRNVV FVDMGHSSFQV

SITAFNKGKLKVLATAFDPYLGGRNFDEALVDYFCEEFKTKYKLNVRDNPRALLRLH QECEKLKKLMSAN

SSDLPLNIECFMNDIDVSSRMNRAQFEDMCAQYLMRVEMPLKTALEQSKLSRDDVYA VELVGGATRIPAV

KDRIAKYFCKDISTTLNADEAVARGCALQCAILSPAFKVREFSITDVVTFPITLRWKTPT EDGLGECEVF

GKNHSAPFSKVITFHKKEPFDLEAFYSNPQELPYPDHRIGCFSVQNVVPQPDGDSSKV KVKVRVNIHGIF

SVSSASLIEKQKGEGEDMQIDSEPMVQNEGRAEEQTKMQVDPEGQNQGDQHNDDSS FNSKEGAAGESQDP

TAGGSKPKVKVKSLDLPIMSSNIRQLDTEVLANFVESERQMISQDKLVKEVNDAKNA

VEEYVYELREKLC

GVYQKYINEEDSNRLTLMLEDTENWLYEDGEDQPKHVYEEKLDALKRLGQPIQERHI
EHEDRPKAFEELG

KKLQLYLKFVDCYRQKDERFVHLSPEEMSIVEKCVNEGMGWLNNKMNAQSKLDITQ
DPVVKVADIIAKIQ

EVEDVCYPVINKPTPKVEETSAGNEQNAEAHNGPAPKQAAEGKADAKGSHQTKAGT
KEMEVE

>hspa4l Gasterosteus aculeatus

MSVVGIDVGYQNCHIGVARSGGIETITNEYSDRCTPACVSLASKNRMIGNAAKSQMT
TNFKNKVHGFKKF

HGRAFDDPFVQAEKPKLPYSLHKLPNGNAGVKVRYLDEDKVFTVEQITGMLLTKLK
ETSESALKKPVVDC

VISVPSFFTDAERRSVFDASQIAGLNCLRLINDTTAVALAYGIYKQDLPTPEERPRNVVF
VDMGHASFQV

SLTAFHKGKLKVLATAFDLHLGGRNFDEALVDFFCEEFKSKYKLNVRENPRALLRLH
QECEKLKKLMSAT

SSDLPLNIECFMNDIDVSSRMNRGQFEDMTAQYLMRVETPLKAVLEQSKLSRDDVYA
VEIVGGATRIPAV

KERIAKFFGKDISTTLNADEAVARGCALQCAILSPAFKVREFSIIDVVPFPITLRWKTPT
EDALGECEVF

SKNHAAPFSKVITFHKKEPFDLEAFYSCPQELPYPDHRIGCFSVQNVAPQPDGESSKV
KVKVRVNVHGIF

SVSSASLIEKQKGEDMQIDSEPMVQTEGGAEEQTKMQVDQEGQGQGDQQNEDNGS
NTKDWACGEKTDAAA

VAAGGSKPRVQVKSTELPVVANNIRQLDRDVLKDFVDCEHQMIIQDKFVKELNDAKN
AVEEYVYELRDKL

CGIYEKYITLDDSNRLTLMLEDTENWLYEEGEDQPKHVYEEKLDALKTLGQPIQDRH
REHEDRPRAFEEL

GKKIQLCIKFVDSYKQKDEQYLQLSAEEMIAVERCVSDSMVWMNSRMNAQSKLGIT
QDPVVKVADIIGKI

QELEDACNPLMNRQRPTVEEAAEVIDQSSAAHNGPAEKQAAAGGQGDAKGSQQATR

GTKEMEVN

>hspa4l Larimichthys crocea

MSVVGIDVGFQNCYIAVARSGGIETIANDYSDRCTPACVSLASKNRMIGNAAKSQMIT
NFKNTVHGFKKF

HGRAFDDPYVQVEKPKLPYSLHKLANGNAGIKVRYLDEDKVFTVEQITGMLLNKLK
ETSEMALKKPVVDC

VISVPSFFTDAERRSVFDATQIAGLNCLRLINDTTAVALAYGIYKQDLPTLEERPRNVVF
VDMGHSSFQV

SITAFNKGKLKVLATAFDPYLGGRNFDEVLVDYFCEEFKGKYKLNVRDNPRALLRLH
QECEKLKKLMSAN

SSDLPLNIECFMNDIDVTSRMNRGHFEDMCAQYLMRVDMPLKVVLEQSKLSRDDIYA
VELVGGATRIPSI

KERISKFFCKDISTTLNADEAVARGSALQCAILSPAFKVREFSITDVVPYPITLRWKSPT
EDGQGECEVF

SKNHAAPFSKVITFHKKEPFDLEAFYNCPQELPYPDHRIGCFSVQNVVPQPDGDSSKV
KVKVRVNVHGIF

SVSSASLIDKQKGEGEDMQIDSEPMVQNEGRAEDQTKMQVDQEGQSQGDQQNEDN
SPNEGAAGEKQDPAA

GGSKPKVKVKSIDLPIKANNIRQLDSDVLNNFVEYERQMIIQDKLVKELNDAKNAVEE
YVYDLRDKLCGI

YEKYITEDDSHRLTLMLEDTENWLYEDGEDQPKHIYEEKLDALKRLGQPIQDRHREH
EDRPRAFDELGKK

LQLYMKFVDCFKQKDERYLHLSVEEMSTVEKCVSENMGWMNTKMNAQSKLGITQD
PIVKVADIIAKIQEL

EDVCNPVINRPKPTVEEAPEVNDQNSGAHNGPTAKQGAEGKGDTKGSQQTKPGTKE
MEVD

>hspa4l Oreochromis niloticus

MSVVGIDVGFQNCYIAVARSGGIETIANEYSDRCTPACVSLASKNRMIGNAAKSQIITN
FKNTVHGFKKF

HGRAFDDPFIQAERPKLPYSLHKLANGNTGIKVRYLDEDKVFTVEQITGMLLTKLKET
SESALKKPVVDC

VISVPSFFTDAERRSVFDATQIAGLNCLRLINDTTAVALAYGIYKQDLPTPEERPRNVVF
VDMGHSSFQV

SITAFHKGKLKVLATAFDPYLGGRNFDEALVDYFCEEFKGKYKLNVKDNPRALLRLY
QECEKLKKLMSAN

SSDLPLNIECFMNDIDVSSRMNRSHFEEMCAQYLMRVEIPLKSALEQSKLSRDDICAV
EIVGGATRIPAI

KERIAKFFCKDVSTTLNADEAVARGCALQCAILSPAFKVREFSITDVVPFPITMRWKSP
TEDGLGECEVF

SKNHAAPFSKVITFHKREPFDLEAFYSNPQELPYSDHRIGFFSVQNVVPQPDGDSSKV
KVKVRVNIHGIF

SVSSASLIEKQKGEGEDMQTDTEPVVQNESRAEEQIKMQVDQESQTQGEPQNEDASS
SSKEGAAGEKQDP

AAGGSKPKVKVKSIDLPIVINNIRQLDSDVLSNFVEYERQMIIQDKLVKELNDAKNAV
EEYVYDLRDKLC

GIYEKYITEGDSNRLTLMLEDTEKWLYEDGEDQPKQVYEEKLDALMRLGQPIQDRHR
EHEDRPRAFEELG

KKLQLYMKFVDSYKQKDERFLHLSAEEMSTVEKCVTESMGWMNSKMNAQSKLALT
QDPVVKVADIIAKIQ

ELEDICNPVINRPKPTVEEAPEVNDQTSGAHNGPTAKQGGAEGKREAKGSQQTKPGA
KEMEVD

>HSPA5 Homo sapiens

MMKFTVVAAALLLLGAVRAEEEDKKEDVGTVVGIDLGTTYSCVGVFKNGRVEIIAN
DQGNRITPSYVAFT

PEGERLIGDAAKNQLTSNPENTVFDAKRLIGRTWNDPSVQQDIKFLPFKVVEKKTKPY
IQVDIGGGQTKT

FAPEEISAMVLTKMKETAEAYLGKKVTHAVVTVPAYFNDAQRQATKDAGTIAGLNV
MRIINEPTAAAIAY

GLDKREGEKNILVFDLGGGTFDVSLLTIDNGVFEVVATNGDTHLGGEDFDQRVMEHFI
KLYKKKTGKDVR

KDNRAVQKLRREVEKAKRALSSQHQARIEIESFFEGEDFSETLTRAKFEELNMDLFRS
TMKPVQKVLEDS

DLKKSDIDEIVLVGGSTRIPKIQQLVKEFFNGKEPSRGINPDEAVAYGAAVQAGVLSGD QDTGDLVLLDV

CPLTLGIETVGGVMTKLIPRNTVVPTKKSQIFSTASDNQPTVTIKVYEGERPLTKDNHL LGTFDLTGIPP

APRGVPQIEVTFEIDVNGILRVTAEDKGTGNKNKITITNDQNRLTPEEIERMVNDAEKF AEEDKKLKERI

DTRNELESYAYSLKNQIGDKEKLGGKLSSEDKETMEKAVEEKIEWLESHQDADIEDFK AKKKELEEIVQP

IISKLYGSGGPPPTGEEDTSEKDEL

>hspa5 Mus musculus

MMKFTVVAAALLLLGAVRAEEEDKKEDVGTVVGIDLGTTYSCVGVFKNGRVEIIAN DQGNRITPSYVAFT

PEGERLIGDAAKNQLTSNPENTVFDAKRLIGRTWNDPSVQQDIKFLPFKVVEKKTKPY IQVDIGGGQTKT

FAPEEISAMVLTKMKETAEAYLGKKVTHAVVTVPAYFNDAQRQATKDAGTIAGLNV MRIINEPTAAAIAY

GLDKREGEKNILVFDLGGGTFDVSLLTIDNGVFEVVATNGDTHLGGEDFDQRVMEHFI KLYKKKTGKDVR

KDNRAVQKLRREVEKAKRALSSQHQARIEIESFFEGEDFSETLTRAKFEELNMDLFRS TMKPVQKVLEDS

DLKKSDIDEIVLVGGSTRIPKIQQLVKEFFNGKEPSRGINPDEAVAYGAAVQAGVLSGD QDTGDLVLLDV

CPLTLGIETVGGVMTKLIPRNTVVPTKKSQIFSTASDNQPTVTIKVYEGERPLTKDNHL LGTFDLTGIPP

APRGVPQIEVTFEIDVNGILRVTAEDKGTGNKNKITITNDQNRLTPEEIERMVNDAEKF AEEDKKLKERI

DTRNELESYAYSLKNQIGDKEKLGGKLSSEDKETMEKAVEEKIEWLESHQDADIEDFK AKKKELEEIVQP

IISKLYGSGGPPPTGEEDTSEKDEL

>hspa5 Gallus gallus

MRHLLLALLLLGGARADDEEKKEDVGTVVGIDLGTTYSCVGVFKNGRVEIIANDQG

NRITPSYVAFTPEG

ERLIGDAAKNQLTSNPENTVFDAKRLIGRTWNDPSVQQDIKYLPFKVVEKKAKPHIQ
VDVGGGQTKTFAP

EEISAMVLTKMKETAEAYLGKKVTHAVVTVPAYFNDAQRQATKDAGTIAGLNVMRII
NEPTAAAIAYGLD

KREGEKNILVFDLGGGTFDVSLLTIDNGVFEVVATNGDTHLGGEDFDQRVMEHFIKLY
KKKTGKDVRKDN

RAVQKLRREVEKAKRALSSQHQARIEIESFFEGEDFSETLTRAKFEELNMDLFRSTMK
PVQKVLEDSDLK

KSDIDEIVLVGGSTRIPKIQQLVKEFFNGKEPSRGINPDEAVAYGAAVQAGVLSGDQDT
GDLVLLDVCPL

TLGIETVGGVMTKLIPRNTVVPTKKSQIFSTASDNQPTVTIKVYEGERPLTKDNHLLGT
FDLTGIPPAPR

GVPQIEVTFEIDVNGILRVTAEDKGTGNKNKITITNDQNRLTPEEIERMVNDAEKFAEE
DKKLKERIDAR

NELESYAYSLKNQIGDKEKLGGKLSSEDKETIEKAVEEKIEWLESHQDADIEDFKSKK
KELEEVVQPIVS

KLYGSAGPPPTGEEEAAEKDEL

>hspa5 Danio rerio

MRLLCLFLLVAGSVFAEEDDKKESVGTVIGIDLGTTYSCVGVYKNGRVEIIANDQGNR
ITPSYVAFTTEG

ERLIGDAAKNQLTSNPENTVFDAKRLIGRTWGDSSVQQDIKYFPFKVIEKKNKPHIQL
DIGSGQMKTFAP

EEISAMVLTKMKETAEAYLGKKVTHAVVTVPAYFNDAQRQATKDAGTIAGLNVMRII
NEPTAAAIAYGLD

KRDGEKNILVFDLGGGTFDVSLLTIDNGVFEVVATNGDTHLGGEDFDQRVMEHFIKLY
KKKTGKDVRKDN

RAVQKLRREVEKAKRALSAQHQARIEIESFFEGEDFSETLTRAKFEELNMDLFRSTMK
PVQKVLEDSDPK

KPDIDEIVLVGGSTRIPKIQQLVKEFFNGKEPSRGINPDEAVAYGAAVQAGVLSGEEET
GDLVLLDVCPL

TLGIETVGGVMTKLIPRNTVVPTKKSQIFSTASDNQPTVTIKVYEGERPLTKDNHLLGT
FDLTGIPPAPR

GVPQIEVTFEIDVNGILRVTAEDKGTGNKNKITITNDQNRLTPEDIERMVNEAERFADE
DKKLKERIDSR

NELESYAYSLKNQIGDKEKLGGKLSSEDKEAIEKAVEEKIEWLEAHQDADLEEFQAK
KKELEEVVQPIVS

KLYGSAGGPPPEEAEEKDEL

>hspa5 Oryzias latipes

MKLLWAVMLVVGAVFAEEEDKKDSVGTVIGIDLGTTYSCVGVFKNGRVEIIANDQGN
RITPSYVAFTSEG

ERLIGDAAKNQLTSNPENTVFDAKRLIGRSWGDSSVQQDIKYFPFKVIEKKSKPHIQID
IGGGQMKTFAP

EEISAMVLTKMKETAEAYLGKKVTHAVVTVPAYFNDAQRQATKDAGTIAGLNVMRII
NEPTAAAIAYGLD

KKDGEKNILVFDLGGGTFDVSLLTIDNGVFEVVATNGDTHLGGEDFDQRVMEHFIKLY
KKKTGKDVRKDN

RAVQKLRREVEKAKRALSAQHQARIEIESFFEGEDFSETLTRAKFEELNMDLFRSTMK
PVQKVLEDSDLK

KSEIDEIVLVGGSTRIPKIQQLVKEFFNGKEPSRGINPDEAVAYGAAVQAGVLSGEEDT
GDVVLLDVCPL

TLGIETVGGVMTKLIPRNTVVPTKKSQIFSTASDNQPTVTIKVYEGERPLTKDNHLLGT
FDLTGIPPAPR

GVPQIEVTFEIDVNGILRVTAEDKGTGNKNKITITNDQNRLTPEDIERMVNDAERFADE
DKRLKERIDAR

NELESYAYSLKNQIGDKEKLGGKLSDDDKETIEKAVEEKIEWMESHQDAETEDFQAK
KKELEEVVQPIIT

KLYGSAGGPPPEGAESEAEKDEL

>hspa5 Gasterosteus aculeatus

MKLLLAVMLVAGTVWADDDDKKESVGTVVGIDLGTTYSCVGVFKNGRVEIIANDQG
NRITPSYVAFTAEG

ERLIGDAAKNQLTSNPENTVFDAKRLIGRTWGDSAVQQDLKYFPFKVTEKKSKPHIQ

VDIGGGQLKTFAP

EEISAMVLTKMKETAEAYLGKKVTHAVVTVPAYFNDAQRQATKDAGTIAGLNVMRII NEPTAAAIAYGLD

KRDGEKNILVFDLGGGTFDVSLLTIDNGVFEVVATNGDTHLGGEDFDQRVMEHFIKLY KKKTGKDVRKDN

RAVQKLRREVEKAKRGLSAQHQARIEIESFFEGEDFSETLTRAKFEELNMDLFRSTMK PVQKVLDDSDLK

KSDIDEIVLVGGSTRIPKIQQLVKELFNGKEPSRGINPDEAVAYGAAVQAGVLSGEEDT GDLVLLDVCPL

TLGIETVGGVMTKLIPRNTVVPTKKSQIFSTASDNQPTVTIKVYEGERPLTKDNHLLGT FDLTGIPPAPR

GVPQIEVTFEIDVNGILRVTAEDKGTGNKNKITITNDQNRLTPEDIERMVNDAERFADE DKKLKERIDAR

NELESYAYSLKNQIGDKEKLGGKLSDEDKETIEKAVEEKIEWMESHQEAELEDFQAK KKELEEVVQPIIS

KLYGSAGAPPPEGAEGEPEEKDEL

>hspa5 Larimichthys crocea

MKLLWVVMLVAGTAFADDDDKKESVGTVVGIDLGTTYSCVGVFKNGRVEIIANDQG NRITPSYVAFTSEG

ERLIGDAAKNQLTSNPENTVFDAKRLIGRTWGDSSVQQDIKYLPFKVTEKKSKPHIQV DIGGGTMKTFAP

EEISAMVLTKMKETAEAYLGKKVTHAVVTVPAYFNDAQRQATKDAGTIAGLNVMRII NEPTAAAIAYGLD

KRDGEKNILVFDLGGGTFDVSLLTIDNGVFEVVATNGDTHLGGEDFDQRVMEHFIKLY KKKTGKDVRKDN

RAVQKLRREVEKAKRGLSAQHQARIEIESFFEGEDFSETLTRAKFEELNMDLFRSTMK PVQKVLEDSDLK

KSDIDEIVLVGGSTRIPKIQQLVKEFFNGKEPSRGINPDEAVAYGAAVQAGVLSGEEDT GDVVLLDVCPL

TLGIETVGGVMTKLIPRNTVVPTKKSQIFSTASDNQPTVTIKVYEGERPLTKDNHLLGT FDLTGIPPAPR

GVPQIEVTFEIDVNGILRVTAEDKGTGNKNKITITNDQNRLTPEDIERMVNDAERFADE DKKLKERIDSR

NELESYAYSLKNQIGDKEKLGGKLSDEDKEAIEKAVEEKIEWMESHQDAELEDFQAK KKELEEVVQPIIS

KLYGSAGGPPPEGAESEQEEKDEL

>HSPA8 Homo sapiens

MSKGPAVGIDLGTTYSCVGVFQHGKVEIIANDQGNRTTPSYVAFTDTERLIGDAAKNQ VAMNPTNTVFDA

KRLIGRRFDDAVVQSDMKHWPFMVVNDAGRPKVQVEYKGETKSFYPEEVSSMVLT KMKEIAEAYLGKTVT

NAVVTVPAYFNDSQRQATKDAGTIAGLNVLRIINEPTAAAIAYGLDKKVGAERNVLIF DLGGGTFDVSIL

TIEDGIFEVKSTAGDTHLGGEDFDNRMVNHFIAEFKRKHKKDISENKRAVRRLRTACE RAKRTLSSSTQA

SIEIDSLYEGIDFYTSITRARFEELNADLFRGTLDPVEKALRDAKLDKSQIHDIVLVGGS TRIPKIQKLL

QDFFNGKELNKSINPDEAVAYGAAVQAAILSGDKSENVQDLLLLDVTPLSLGIETAGG VMTVLIKRNTTI

PTKQTQTFTTYSDNQPGVLIQVYEGERAMTKDNNLLGKFELTGIPPAPRGVPQIEVTF DIDANGILNVSA

VDKSTGKENKITITNDKGRLSKEDIERMVQEAEKYKAEDEKQRDKVSSKNSLESYAF NMKATVEDEKLQG

KINDEDKQKILDKCNEIINWLDKNQTAEKEEFEHQQKELEKVCNPIITKLYQSAGGMP GGMPGGFPGGGA

PPSGGASSGPTIEEVD

>hspa8 Mus musculus

MSKGPAVGIDLGTTYSCVGVFQHGKVEIIANDQGNRTTPSYVAFTDTERLIGDAAKNQ VAMNPTNTVFDA

KRLIGRRFDDAVVQSDMKHWPFMVVNDAGRPKVQVEYKGETKSFYPEEVSSMVLT KMKEIAEAYLGKTVT

NAVVTVPAYFNDSQRQATKDAGTIAGLNVLRIINEPTAAAIAYGLDKKVGAERNVLIF

DLGGGTFDVSIL

TIEDGIFEVKSTAGDTHLGGEDFDNRMVNHFIAEFKRKHKKDISENKRAVRRLRTACE
RAKRTLSSSTQA

SIEIDSLYEGIDFYTSITRARFEELNADLFRGTLDPVEKALRDAKLDKSQIHDIVLVGGS
TRIPKIQKLL

QDFFNGKELNKSINPDEAVAYGAAVQAAILSGDKSENVQDLLLLDVTPLSLGIETAGG
VMTVLIKRNTTI

PTKQTQTFTTYSDNQPGVLIQVYEGERAMTKDNNLLGKFELTGIPPAPRGVPQIEVTF
DIDANGILNVSA

VDKSTGKENKITITNDKGRLSKEDIERMVQEAEKYKAEDEKQRDKVSSKNSLESYAF
NMKATVEDEKLQG

KINDEDKQKILDKCNEIISWLDKNQTAEKEEFEHQQKELEKVCNPIITKLYQSAGGMP
GGMPGGFPGGGA

PPSGGASSGPTIEEVD

>hspa8 Gallus gallus

MSKGPAVGIDLGTTYSCVGVFQHGKVEIIANDQGNRTTPSYVAFTDTERLIGDAAKNQ
VAMNPTNTVFDA

KRLIGRRFDDSVVQSDMKHWPFTVVNDAGRPKVQVEYKGETKSFYPEEISSMVLTK
MKEIAEAYLGKTVT

NAVVTVPAYFNDSQRQATKDAGTIAGLNVLRIINEPTAAAIAYGLDKKVGAERNVLIF
DLGGGTFDVSIL

TIEDGIFEVKSTAGDTHLGGEDFDNRMVNHFIAEFKRKHKKDISENKRAVRRLRTACE
RAKRTLSSSTQA

SIEIDSLYEGIDFYTSITRARFEELNADLFRGTLDPVEKALRDAKLDKSQIHDIVLVGGS
TRIPKIQKLL

QDFFNGKELNKSINPDEAVAYGAAVQAAILSGDKSENVQDLLLLDVTPLSLGIETAGG
VMTVLIKRNTTI

PTKQTQTFTTYSDNQPGVLIQVYEGERAMTKDNNLLGKFELTGIPPAPRGVPQIEVTF
DIDANGILNVSA

VDKSTGKENKITITNDKGRLSKEDIERMVQEAEKYKAEDEKQRDKVSSKNSLESYAF
NMKATVEDEKLQG

KISDEDKQKILDKCNEIINWLDKNQTAEKEEFEHQQKELEKVCNPIITKLYQSAGGMP
GGMPGGFPGGGA

PPSGGASSGPTIEEVD

>hspa8 Danio rerio

MSKGPAVGIDLGTTYSCVGVFQHGKVEIIANDQGNRTTPSYVAFTDTERLIGDAAKNQ
VA

MNPTNTVLDANRLNGRQFDDGVVQSDMKHWPFNVINDNSRPKVQVEYKGESKSFY
PEEIS

SMVLTKMKEIAEAYLGKTVSNAVITVPAYSNDSQRQATKDAGTISGLNVLVIINEPTAA
A

IAYGLDKKVGAERNVLIFDLGGGSFDVSILTIEDGIFEVKSTAGDTHLGGEDFDNRMV
NH

FITEFKRKHKKDISDNKRAVRRLRTACERAKRTLSSSTQASIEIDSLYEGIDFYTSITRA

RFEELNADLFRGTLDPVEKALRDAKMDKAQIHDIVLVGGSTRIPKIQKLLQDYFNGKE
LN

KSINPDEAVAYGAAVQAAILSGDKSENVQDLLLLDVTPLSLGIETAGGVMTVLIKRNT
TI

PTKQTQTFTTYSDNQPGVLIQVYEGERAMTKDNNLLGKFELTGIPPAPRGVPQIEVTF
DI

DANGIMNVSAVDKSTGKENKITITNDKGRLSKEDIERMVQEAEKYKAEDDVQRDKV
SAKN

GLESYAFNMKSTVEDEKLKGKISDEDKQKILDKCNEVIGWLDKNQTAEREEFEHQQK
ELE

KVCNPIITKLYQSAGGMPGGMPEGMPGGFPGAGAAPGGGSSGPTIEEVD

>hspa8 Oryzias latipes

MSKGPAVGIDLGTTYSCVGVFQHGKVEIIANDQGNRTTPSYVAFTDTERLIGDAAKNQ
VAMNPTNTVFDA

KRLIGRRFDDHVVQSDMNDWPFNVINDNTRPKVQVEYKGETKSFYPEEVSSMVLTK
MKEIAEAYLGKTVN

NAVITVPAYFNDSQRQATKDAGTISGLNVLRIINEPTAAAIAYGLDKKVGSERNVLIFD
LGGGTFDVSIL

TIEDGIFEVKSTAGDTHLGGEDFDNRMVNHFIAEFKRKYKKDISDNKRAVRRLRSACE
RAKRTLSSSTQA

SIEIDSLYEGVDFYTSITRARFEELNADLFRGTLDPVEKSLRDAKMDKGQIHDIVLVGG
STRIPKIQKLL

QDFFNGKELNKSINPDEAVAYGAAVQAAILSGDKSENVQDLLLLDVTPLSLGIETAGG
VMTVLIKRNTTI

PTKQTQTFTTYSDNQPGVLNQVYEVGAMTKDNNLLGKFELTGIPPALWCPQIEVTFDI
DANGIMNVSAVD

KSTGKENKITITNDKGRLSKEDIERMVQEAEKYKAEDDVQRDKVSAKNGLESYAFN
MKSTVEDEKLAGKI

SDEDKQKILDKCNEVISWLDKNQTAERDEYVHQQKELEKVCNPIITKLYQSAGGMPG
GCQRECQEVSLEL

VVLLAVALLDQPLKKLIKHSMTSPSKIFTKKTLFKCNMQSIKSVMRLNQFPFITIT

>hspa8a Gasterosteus aculeatus

MSKGPAVGIDLGTTYSCVGVFQHGKVEIIANDQGNRTTPSYVAFTDSERLIGDAAKNQ
VA

MNPTNTVFADAKRLIGRRFDDAVVQSDMKHWPFNVINDNTRPKVQVEYKGESKSFY
PEEV

SSMVLTKMKEIAEAYLGKTVNNAVVTVPAYFNDSQRQATKDAGTISGLNVLRIINEPT
AA

AIAYGLDKKVGAERNVLIFDLGGGTFDVSILTIEDGIFEVKSTAGDTHLGGEDFDNRM
VN

HFIAEFKRKYKKDISDNKRAVRRLRTACERAKRTLSSSTQASIEIDSLYEGVDFYTSITR

ARFEELNADLFRGTLDPVEKSLRDAKMDKGQIHDIVLVGGSTRIPKIQKLLQDFFNGK
EL

NKSINPDEAVAYGAAVQAAILSGDKSENVQDLLLLDVTPLSLGIETAGGVMTVLIKRN
TT

IPTKQTQTFTTYSDNQPGVLIQVYEGERAMTRDNNLLGKFELTGIPPAPRGVPQIEVTF
D

IDANGIMNVSAVDKSTGKENKITITNDKGRLSKEDIERMVQEAEKYKAEDDVQRDKV
SSK

NALESYAFNMKSTVEDEKLAGKISDDDKTKILEKCKEVISWLDKNQTAERDEYEHQQ KEL

EKVCNPIITKLYQSAGGMPEGMAGGFPGAGGAAPGSGGSSGPTIEEVD

>hspa8b Gasterosteus aculeatus

MSKGPAVGIDLGTTYSCVGVFQHGKVEIIANDQGNRTTPSYVAFTDTERLIGDAAKNQ VA

LNPNNTVFDAKRLIGRRFDDNVVQSDMKHWPFTVIDDSTRPKVQVEYKGETKAFYP EEIS

SMVLIKMKEIAEAYLGKTVTNAVVTVPAYFNDSQRQATKDAGTISGLNVLRIINEPTA AA

IAYGLDKKVGAERNVLIFDLGGGTFDVSILTIEDGIFEVKSTAGDTHLGGEDFDNRMV NH

FISEFKRKYKKDISDNKRAVRRLRTACERAKRTLSSSTQASIEIDSLYEGVDFYTSITRA

RFEELNADLFRGTLEPVEKSLRDAKMDKAQIHDIVLVGGSTRIPKIQKMLQDLFNGKE LN

KSINPDEAVAYGAAVQAAILSGDKSENVQDLLLLDVTPLSLGIETAGGVMTVLIKRNT TI

PTKQTQTFTTYSDNQPGVLIQVFEGERAMTKDNNHLGKFELTGIPPAPRGVPQIEVTF DI

DANGIMNVSAADKSTGKENKITITNDKGRLSKEDIERMVQEADKYKAEDDVQREKV SAKN

GLESYAFNMKSTVEDEKLKDKISEEDRQKIVEKCSEVITWLDGNQSAEKEEFEHQQK ELE

KLCNPIMTKLYQNAGGMPGGMPGGMPGGMPGGFGGAGGAPGGGASSGPTIEEVD

>hspa8 Larimichthys crocea

MSKGPAVGIDLGTTYSCVGVFQHGKVEIIANDQGNRTTPSYVAFTDTERLIGDAAKNQ VALNPNNTVFDA

KRLIGRRFDDSVVQSDMKHWPFTVINDASRPKVKVEYKGETKTFYPEEISSMVLIKM KEIAEAYLGKTVT

NAVVTVPAYFNDSQRQATKDAGTISGLNVLRIINEPTAAAIAYGLDKKVGSERNVLIFD LGGGTFDVSIL

TIEDGIFEVKSTAGDTHLGGEDFDNRMVNHFISEFKRKFKKDISDNKRAVRRLRTACE RAKRTLSSSTQA

SIEIDSLYEGVDFYTSITRARFEELNADLFRGTLEPVEKSLRDAKMDKAQIHDIVLVGG STRIPKIQKLL

QDFFNGKDLNKSINPDEAVAYGAAVQAAILSGDKSENVQDLLLLDVTPLSLGIETAGG VMTVLIKRNTTI

PTKQTQTFTTYSDNQPGVLIQVFEGERAMTKDNNLLGKFELVGIPPAPRGVPQIEVTF DIDANGIMNVSA

VDKSTGKENKITITNDKGRLSKEDIERMVQEAERYKAEDDVQRDKVTAKNALESYAF NMKSTVEDEKLKD

KISDEDKKKIVDKCNEIISWLDRNQSAEKDEFEHQQKELEKICNPIMTKLYQSAGGAP GGMPGGFPGAGG

APGGGASSGPTIEEVD

>hspa8 Oreochromis niloticus

MSKGPAVGIDLGTTYSCVGVFQHGKVEIIANDQGNRTTPSYVAFTDTERLIGDAAKNQ VAMNPTNTVFDA

KRLIGRRFDDPVVQSDMKHWPFNVINDNSRPKVQVEYKGETKSFYPEEISSMVLTKM KEIAEAYLGKTVN

NAVITVPAYFNDSQRQATKDAGTISGLNVLRIINEPTAAAIAYGLDKKVGSERNVLIFD LGGGTFDVSIL

TIEDGIFEVKSTAGDTHLGGEDFDNRMVNHFIAEFKRKYKKDISDNKRAVRRLRTACE RAKRTLSSSTQA

SIEIDSLYEGVDFYTSITRARFEELNADLFRGTLDPVEKSLRDAKMDKGQIHDIVLVGG STRIPKIQKLL

QDFFNGKELNKSINPDEAVAYGAAVQAAILSGDKSENVQDLLLLDVTPLSLGIETAGG VMTVLIKRNTTI

PTKQTQTFTTYSDNQPGVLIQVYEGERAMTKDNNLLGKFELTGIPPAPRGVPQIEVTF DIDANGIMNVSA

VDKSTGKENKITITNDKGRLSKEDIERMVQEAEKYKAEDDVQRDKVAAKNGLESYA FNMKSTVEDEKLAG

KISDDDKQKILDKCNEVISWLDKNQTAEKDEYEHQQKELEKVCNPIITKLYQSAGGM

PGGMPEGMPGGFP

GAGGAAPGGGSSGPTIEEVD

>HSPA9 Homo sapiens

MISASRAAAARLVGAAASRGPTAARHQDSWNGLSHEAFRLVSRRDYASEAIKGAVVG
IDLGTTNSCVAVM

EGKQAKVLENAEGARTTPSVVAFTADGERLVGMPAKRQAVTNPNNTFYATKRLIGRR
YDDPEVQKDIKNV

PFKIVRASNGDAWVEAHGKLYSPSQIGAFVLMKMKETAENYLGHTAKNAVITVPAYF
NDSQRQATKDAGQ

ISGLNVLRVINEPTAAALAYGLDKSEDKVIAVYDLGGGTFDISILEIQKGVFEVKSTNG
DTFLGGEDFDQ

ALLRHIVKEFKRETGVDLTKDNMALQRVREAAEKAKCELSSSVQTDINLPYLTMDSS
GPKHLNMKLTRAQ

FEGIVTDLIRRTIAPCQKAMQDAEVSKSDIGEVILVGGMTRMPKVQQTVQDLFGRAPS
KAVNPDEAVAIG

AAIQGGVLAGDVTDVLLLDVTPLSLGIETLGGVFTKLINRNTTIPTKKSQVFSTAADG
QTQVEIKVCQGE

REMAGDNKLLGQFTLIGIPPAPRGVPQIEVTFDIDANGIVHVSAKDKGTGREQQIVIQS
SGGLSKDDIEN

MVKNAEKYAEEDRRKKERVEAVNMAEGIIHDTETKMEEFKDQLPADECNKLKEEISK
MRELLARKDSETG

ENIRQAASSLQQASLKLFEMAYKKMASEREGSGSSGTGEQKEDQKEEKQ

>hspa9 Mus musculus

MISASRAAAARLVGTAASRSPAAARPQDGWNGLSHEAFRFVSRRDYASEAIKGAVVG
IDLGTTNSCVAVM

EGKQAKVLENAEGARTTPSVVAFTADGERLVGMPAKRQAVTNPNNTFYATKRLIGRR
YDDPEVQKDTKNV

PFKIVRASNGDAWVEAHGKLYSPSQIGAFVLMKMKETAENYLGHTAKNAVITVPAYF
NDSQRQATKDAGQ

ISGLNVLRVINEPTAAALAYGLDKSEDKVIAVYDLGGGTFDISILEIQKGVFEVKSTNG
DTFLGGEDFDQ

ALLRHIVKEFKRETGVDLTKDNMALQRVREAAEKAKCELSSSVQTDINLPYLTMDAS GPKHLNMKLTRAQ

FEGIVTDLIKRTIAPCQKAMQDAEVSKSDIGEVILVGGMTRMPKVQQTVQDLFGRAPS KAVNPDEAVAIG

AAIQGGVLAGDVTDVLLLDVTPLSLGIETLGGVFTKLINRNTTIPTKKSQVFSTAADG QTQVEIKVCQGE

REMAGDNKLLGQFTLIGIPPAPRGVPQIEVTFDIDANGIVHVSAKDKGTGREQQIVIQS SGGLSKDDIEN

MVKNAEKYAEEDRRKKERVEAVNMAEGIIHDTETKMEEFKDQLPADECNKLKEEISK MRALLAGKDSETG

ENIRQAASSLQQASLKLFEMAYKKMASEREGSGSSGTGEQKEDQKEEKQ

>hspa9 Gallus gallus

MISASRAAARLPLLLPRGGPVPAVPGLAQTFWNGLSQNVLRAASSRKYASEAIKGAVI GIDLGTTNSCVA

VMEGKQAKVLENSEGARTTPSVVAFTADGERLVGMPAKRQAVTNPHNTFYATKRLIG RRFDDSEVKKDIK

NVPFKIVRASNGDAWVEAHGKLYSPSQIGAFVLMKMKETAENYLGHPAKNAVITVPA YFNDSQRQATKDA

GQISGLNVLRVINEPTAAALAYGLDKSEDKIIAVYDLGGGTFDISILEIQKGVFEVKSTN GDTFLGGEDF

DQALLQYIVKEFKRETGVDLTKDNMALQRVREASEKAKCELSSSVQTDINLPYLTMD ASGPKHLNMKLSR

SQFEGIVADLIKRTVAPCQKAMQDAEVSKSDIGEVILVGGMTRMPKVQQTVQDLFGR APSKAVNPDEAVA

IGAAIQGGVLAGDVTDVLLLDVTPLSLGIETLGGVFTKLINRNTTIPTKKSQVFSTAAD GQTQVEIKVCQ

GEREMASDNKLLGQFTLVGIPPAPRGVPQIEVTFDIDANGIVHVSAKDKGTGREQQIVI QSSGGLSKDEI

ENMVKNAEKYAEEDRRRKERVEAVNLAEGIIHDTESKMEEFKDQLPADECNKLKEEI AKMRELLARKDTE

TGENIRQAATSLQQASLKLFEMAYKKMASERESSGSSGDQKEEKQ

>hspa9 Danio rerio

MLSVSRTARLVRNVSCSQKTSSGVSDLIKKACLNGWTQKTLQTAARRHYASEAIRGA VIGIDLGTTNSCV

AVMDGKNAKVLENAEGARTTPSVVAFTSDGERLVGMPAKRQAVTNPNNTLYATKRLI GRRFDDAEVQKDL

KNVPYKIVRASNGDAWLEVHGKMYSPSQAGAFILIKMKETAESYLGQSVKNAVVTV PAYFNDSQRQATKD

AGQIAGLNVLRVINEPTAAALAYGLDKTQDKIIAVYDLGGGTFDISVLEIQKGVFEVKS TNGDTFLGGED

FDQHLLRHIVKEFKKESGVDLMKDNMALQRVREAAEKAKCELSSSLQTDINLPYLTM DASGPKHLNMKLT

RSQFEGIVADLIRRTVAPCQKAMQDAEVSKSDIGEVLLVGGMTRMPKVQQTVQDLFG RAPSKSVNPDEAV

AIGAAIQGGVLAGDVTDVLLLDVTPLSLGIETLGGVFTKLINRNTTIPTKKSQVFSTAA DGQTQVEIKVC

QGEREMATDNKVLGQFTLVGIPPALRGVPQIEVTFDIDANGIVHVSAKDKGTGREQQI VIQSSGGLSKDD

IENMVKNAEKYAEEDRRRKDRVEAVNMAEGIVHDTESKMEEFKDQLPADECNKLKE EISKVRELLSRKDT

ETGENIKQAATSLQQASLKLFEMAYKKMASEREGSSGSSSSGEAGEKKEGQQ

>hspa9 Oryzias latipes

MLSAARTISRSLPTRSCSRNVSSLVKKACWSGFKPDVLGNLSRRNYASEAIKGSVIGID LGTTNSCVAVM

DGKQAKVLENAEGARTTPSVVAFTAEGERLVGMPAKRQAVTNPQNTLYATKRLIGRR YDDPEVQKDLKNV

PYKIVRASNGDAWVEAHGKMYSPSQMGAFVLMKMKETAENFMGTKVKNAVITVPA YFNDSQRQATKDAGQ

IAGLNVLRVINEPTAAALAYGLDKSQDKIIAVYDLGGGTFDVSVLEIQKGVFEVKSTN GDTFLGGEDFDQ

QLLKHIVKEFKRESGVDLMKDNMALQRVREAAEKAKCELSSSLQTDINLPYLTMDAS GPKHLNMKLTRSQ

FEGIVADLIRRTVAPCQKAMQDAEVSKGDIGEVLLVGGMTRMPKVQQTVQDLFGRAP SKSVNPDEAVAIG

AAIQGGVLAGDVTDVLLLDVTPLSLGIETLGGVFTKLINRNTTIPTKKSQVFSTAADG QTQVEIKVCQGE

REMALDNKVLGQFSLVGIPPAPRGVPQIEVTFDIDANGIVHVSARDKGTGREQQIVIQS SGGLSKDDIEN

MIKNAERYAEEDRRRKERVEAVNMAEGIIHDTESKMEEFKDQLPADECSKLKEEITKV RDILANKEAETG

ENIKQAANSLQQASLKLFEVAYKKMAAERDGGSSGGSSSSSTEGEKKEGQQ

>hspa9 Gasterosteus aculeatus

MLTAATCVSRTLPTRNIARNVSSIVKVCWTGGFTPNALRALSRRDYASEAIKGAVIGID LGTTNSCVAVM

EGKQAKVLENAEGARTTPSVVAFTAEGERLVGMPAKRQSVTNPQNTLYATKRLIGRR YDDAEVQKDLKNV

PFKIVRASNGDAWVEVHGKMYSPSQAGAFVLIKMKETAENYLGTKVKNAVVTVPAY FNDSQRQATKDAGQ

IAGLNVLRVINEPTAAALAYGLDKTQDKIIAVYDLGGGTFDISVLEIQKGVFEVKSTNG DTFLGGEDFDQ

HLLQHIVKEFKKESGVDLMKDSMGLQRVREAAEKAKCELSSSLQTDINLPYLTMDAS GPKHLNMKLTRSQ

FEGIVGDLIRRTVAPCQKAMQDAEVSKGDIGEVLLVGGMSRMPKVQQTVQDLFGRA PSKSVNPDEAVAIG

AAIQGGVLAGDVTDVLLLDVTPLSLGIETLGGVFTKLINRNTTIPTKKSQVFSTAADG QTQVEIKVCQGE

REMAADNKVLGQFTLVGIPPAPRGVPQVEVTFDIDANGIVHVSAKDKGTGREQQIVIQ SSGGLSKDDIEN

MIKNAEKYAEEDMRRKDRVEAVNMAEGIVHDTEAKMEEFKEQLPADECTKLKEEISK VRDLLANKDSETG

ENIKQAATTLQQASLKLFEMAYKKMAAERDGGGSSSSSSGSSSSEGEKKEGQQ

>hspa9 Larimichthys crocea

MLSVARTVSRTLPTGTCTGNVSSLIKKACWGGFQSDALRALSRRDYASEAVKGAVIGI

DLGTTNSCVAVM

EGKQAKVLENAEGARTTPSVIAFTADGERLVGMPAKRQSVTNPQNTLYATKRLIGRRY
DDPEVQKDLKNV

PFKIVRASNGDAWVEAHGKMYSPSQAGAFVLMKMKETAESYLGTKVKNAVVTVPA
YFNDSQRQATKDAGQ

IAGLNVLRVINEPTAAALAYGLDKTQDKIIAVYDLGGGTFDISVLEIQKGVFEVKSTNG
DTFLGGEDFDQ

HLLKHIVKEFKRESGVDLTKDNMALQRVREAAEKAKCELSSSLQTDINLPYLTMDAS
GPKHLNMKLTRSQ

FEGIVADLIRRTVAPCQKAMQDAEVSKGDIGEVLLVGGMSRMPKVQQTVQDLFGRAP
SKSVNPDEAVAIG

AAIQGGVLAGDVTDVLLLDVTPLSLGIETLGGVFTKLINRNTTIPTKKSQVFSTAADG
QTQVEIKVCQGE

REMAADNKVLGQFTLVGIPPAPRGVPQIEVTFDIDANGIVHVSAKDKGTGREQQIVIQ
SSGGLSKDDIEN

MIKNAEKYAEEDRRRKDRVEAVNTAEGIVHDTESKMEEFKDQLPADECTKLKEEIAK
VRELLANKDSETG

ENIKQAATTLQQSSLKLFEMAYKKMAAERDSSSSSSSSGSSEGEKKEGQQ

>hspa9 Oreochromis niloticus

MLSVTRSVSRTLPTRSNIRSVSSLIKKACWTGFQPDALRALSRRDYASEAVKGAVIGID
LGTTNSCVAVM

EGKQAKVLENAEGARTTPSVIAFTADGERLVGMPAKRQAVTNPQNTLYATKRLIGRRF
EDPEVQKDLKNV

PYKIVRASNGDAWVEAHGKMYSPSQAGAFVLMKMKETAESYLGSTVKNAVITVPAY
FNDSQRQATKDAGQ

IAGLSVLRVINEPTAAALAYGLDKTQDKIIAVYDLGGGTFDISILEIQKGVFEVKSTNGD
TFLGGEDFDQ

HLLRHIVKEFKRESGVDLTKDNMALQRVREAAEKAKCELSSSLQTDINLPYLTMDAS
GPKHLNMKLTRAQ

FEGIVADLIRRTVAPCQKAMQDAEVSKGDIGEVLLVGGMTRMPKVQQTVQDLFGRAP
SKSVNPDEAVAIG

AAIQGGVLAGDVTDVLLLDVTPLSLGIETLGGVFTKLINRNTTIPTKKSQVFSTAADG QTQVEIKVCQGE

REMATDNKLLGQFTLVGIPPAPRGVPQIEVTFDIDANGIVHVSAKDKGTGREQQIVIQS SGGLSKDDIEN

MIKNAEKYAEEDRRRKDRVEAVNMAEGIIHDTESKMEEFKDQLPADECTKLKEEISK VRNLLTNKDSETG

ENIKQAATDLQQASLKLFEMAYKKMAAERDSTSSSSSSSEGEKKEGQQ

>HSPA12A Homo sapiens

MADKEAGGSDGPRETAPTSAYSSPARSLGDTGITPLSPSHIVNDTDSNVSEQQSFLVVV AVDFGTTSSGY

AYSFTKEPECIHVMRRWEGGDPGVSNQKTPTTILLTPERKFHSFGYAARDFYHDLDPN EAKQWLYLEKFK

MKLHTTGDLTMDTDLTAANGKKVKALEIFAYALQYFKEQALKELSDQAGSEFENSDV RWVITVPAIWKQP

AKQFMRQAAYQAGLASPENSEQLIIALEPEAASIYCRKLRLHQMIELSSKAAVNGYSG SDTVGAGFTQAK

EHIRRNRQSRTFLVENVIGEIWSELEEGDKYVVVDSGGGTVDLTVHQIRLPEGHLKEL YKATGGPYGSLG

VDYEFEKLLYKIFGEDFIEQFKIKRPAAWVDLMIAFESRKRAAAPDRTNPLNITLPFSFI DYYKKFRGHS

VEHALRKSNVDFVKWSSQGMLRMSPDAMNALFKPTIDSIIEHLRDLFQKPEVSTVKF LFLVGGFAEAPLL

QQAVQAAFGDQCRIIIPQDVGLTILKGAVLFGLDPAVIKVRRSPLTYGVGVLNRYVEGK HPPEKLLVKDG

TRWCTDVFDKFISADQSVALGELVKRSYTPAKPSQLVIVINIYSSEHDNVSFITDPGVK KCGTLRLDLTG

TSGTAVPARREIQTLMQFGDTEIKATAIDIATSKSVKVGIDFLNY

>hspa12a Mus musculus

MADKEAGGGDAGPRETAPTSTYSSPARSLGDTGITPLSPSHILNDADPVSEQQTFLVV VAIDFGTTSSGY

AYSFTKEPECIHVMRRWEGGDPGVSNQKTPTTILLTPERKFHSFGYAARDFYHDLDPS

EAKQWLYLEKFK

MKLHTTGDLTMDTDLTAANGKKVKALEIFAYALQYFKEQALKELSDQAGSDFENSD
VRWVITVPAIWKQP

AKQFMREAAYQAGLASPENSEQLIIALEPEAASIYCRKLRLHQMIELSSKAVVNGYSA
SDTVGAGFAQAK

EHVRRNRQSRTFLVENVIGEIWSELEEGDKYVVVDSGGGTVDLTVHQIRLPEGHLKEL
YKATGGPYGSLG

VDYEFEKLLCKIFGEDFIEQFKIKRPAAWVDLMIAFESRKRAAAPDRTNPLNITLPFSFI
DYYKKFRGHS

VEHALRKSNVDFVKWSSQGMLRMSPDAMNALFKPTIDSIIEHLRDLFQKPEVSTVKF
LFLVGGFAEAPLL

QQAVQTAFGDKCRIIIPQDVGLTILKGAVLFGLDPAVIKVRRSPLTYGVGVLNRYVEGK
HPPEKLLVKDG

TRWCTDVFDKFISADQSVALGELVKRSYTPAKPSQLVIIINIYSSEHDNVSFITDPGVKK
CGTLRLDLTG

SGGTAVPARREIQTIMQFGDTEIKATAVDITTSKSVKVGIDFLNY

>hspa12a Gallus gallus

MRRWEGGDPGVSNQKTPTTILLTPERKFHSFGYAARDFYHDLDPSESKHWLYFEKFK
MKLHTTSNLTMET

DLTAANGKKVKALEIFAYALQFFKEQALKELSDQAGSDFENTEVRWVITVPAIWKQPA
KQFMRQAAYKAG

MASPENPEQLIIALEPEAASIYCRKLRLHQMIELSSRAPVNGYSPSDTIGTGFTQAKEH
VRRNRQSRTFL

VENVIGEIWSELEEGDRYIVVDSGGGTVDMTVHQIRLPEGHLKELYKATGGPYGSLG
VDYEFEKLLCKIF

GEDFIEQFKIKRPAAWVDLMIAFESRKRAAAPDRTNPLNITLPFSFIDYYKKFRGHSVE
HALRKSNVDFV

KWSSQGMLRMSPDAMNALFKPTIDQIVQHLSDVFDKPEVTNVKFLFLVGGFAESPLL
QQAVQSAFGSRCR

VIIPQDVGLTILKGAVLFGLDPAVIKVRRSPLTYGVGVLNRFVEGRHPPEKLLIKDGTR
WCTDVFDKFIS

ADQSVALGETVTRSYTPAKPSQLVIVINIYSSEQDNVSFITESGVKKCGTLRLDLTGTD
ASVPNRREIKT

LMQFGDTEIKAMAIDVATSKSVKVGIDFLNY

>hspa12a Danio rerio

MANSVLYIAIDFGTSYSGYAISFKTKQPQESIQIPNWGVNFGYNTFKTPTCILFDEHETF
QKFGYDAMMT

YTRSTPKSQARKSFLFEHFKMELYDKEIHRDLMITAKNGGQMKALTVFSESLRYLKD
HALEKIKENTTGK

TFIASDVTWVLTVPAIWNAAAKQFMREAAIEAGLVIESEPERLVFALEPEAASIYCKHL
PSEGYISEEAC

RDTLEQKPGTQYMVVDCGGGTIDITVHEVVEDGKLKELNAASGNDMGGQTVDRKFI
SFLKEIFSEKIYNK

FEQNFPAEALKLKYDIALAKSCDQLVLIQCPVTLQELAKKEKAIESYFEGVEGAEWDE
GSIIIKEDKLQS

FFDESLKTTAEKLEKIMSNPELNIEYMLLVGGFAECKILKKFLKERFDKCKIVCPVEPQ
VVIIKGAIRYA

KQPKVVKSRMSALTYGIRIDAPFDEVLHKGKTSYVNSERQTYCDVCFETFVRKGESV
NCDEVRHFFFNPS

HTNQTFATFQFFSTNEKQVQFIDEPGVELVGVFQVGMPKTDGGLSREIKLEIKFGSTE
MKATATDVDSGE

TTSVKLDFINS

>hspa12a Oryzias latipes

MSEKDTATDKILTDAMANPSPAKSIGDPGITPLSPSHIQQNDSDQVQSGPSFVVVVAID
F

GTTSSGYAYAFTKEPECIHTMRRWEGGDPGVSNQKTPTTILLTPDRKFHSFGYAARDF
YH

DLDPSESKQWLYLEKFKMKLHTTPNLSINTDLHAANGKRVKALDIFAYALAFFKEQA
LKE

LSDQTGGDFDNNDVRWVITVPAIWKMPAKQFMREAAYKSGLVSRDTPEQLIIALEPE
AAS

IYCRKLRLHQMVDLATSNTQNGFNPNDNVGSGVSQAKEHVRRNRQSRTFLVENVIGE

LWS

ELEEGDRYVVVDCGGGTVDLTVHQIRLPEGHLKELYKASGGPYGSIGIDFEFEKLLCRI
F

GQDFIDQFKIKRPAAWVDLMIAFESRKRAAAPDRTNPLNINLPFSFIDYYKKFRGHSVE
H

ALRKSNVDFVKWSSQGMLRMSPDAMNSLFKPTIDHIIQHLTELFEKPEVCDIKFLFLV
GG

FAESPLLQQAVQNMLQGRCRIIIPHDVGLTILKGAVLFGLDPSVIKVRRSPLTYGVGVL
N

RFVEGKHPPEKLLVKDGTRWCTDVFDTFIAADQSVALGEMVKRSYTPAKPSQQVIVIH
VY

CSEKESVSFISEPGVRKCGTLRLDVSGTESTAQRREIQTLMQFGDTEIRAMAVDVSTG
RT

VKAGIDFLSH

>hspa12a Gasterosteus aculeatus

MMTEREAAKEETLTDAMANPSPAKSMGDPGITPLSPSHTQQNDTDQVPSGPSFVVVV
AIDFGTTSSGYAY

AFTKEPECIHTMRRWEGGDPGVSNQKTPTTILLTPDRKFHSFGYAARDFYHDLDPSES
KQWLYLEKFKMK

LHTTANLSIDTDLHAANGKRVKALDIFAYALAFFKEQALKELSDQTGIEFDNNDVRW
VITVPAIWKMPAK

QFMREAAYKSGLVSRENPEQLIIALEPEAASIYCRKLRLHQMVDLGTLTTQNGCSPTD
NVGSGMTQGDRY

VVVDCGGGTVDLTVHQIRLPEGHLKELYKASGGPYGSLGIDYAFEKLLCKIFGQDFID
QFKIKRPAAWVD

LMIAFESRKRAAAPDRTNPLNINLPFSFIDYYKKFRGHSVEHALRKSNVDFVKWSSQG
MLRMSPEAMNSL

FKPTIDHIIQHLTELFEKPEVSDIKFLFLVGGFAESPLLQQAVQDMLQGRSRIIIPHDVGL
TILKGAVLF

GLDPTIIKVRRSPLTYGVGVLNRFVEGKHPPEKLLVKDGTRWCTDVFDTFIAADQSVA
LGEMVKRSYTPA

KPSQQVIVIHVYCSEKEKAGFISEPGVRKCGTLRLDVSGTESTAPRREIQTLMQFGDTEIRAMAVDVSTG

RTVKASIDFLSH

>hspa12a Larimichthys crocea

MMTEKETAAEKIISGRRAKYQKKETTALKDAMANPSPAKSMGDPGITPLSPSHTQQNDTDQVPSGPSFVV

VVAIDFGTTSSGYAYAFTKEPECIHTMRRWEGGDPGVSNQKTPTTILLTPDRKFHSFGYAARDFYHDLDP

SESKHWLYLEKFKMKLHTTANLSIDTDLHAANGKRVKALDIFAYALAFFKEQALKELSDQTGGEFDNNDV

RWVITVPAIWKMPAKQFMREAAYKSALVSRENPEQLIIALEPEAASIYCRKLRLHQMVDLGTQTTQNGFS

PTENVGSGMTQGDRYVVVDCGGGTVDLTVHQIRLPEGHLKELYKASGGPYGSLGIDYEFEKLLCKIFGQD

FIDQFKIKRPAAWVDLMIAFESRKRAAAPDRTNPLNINLPFSFIDYYKKFRGHSVEHALRKSK

>hspa12a Oreochromis niloticus

MMTEKDASTEKIITDAMADPSPAKSMGDPGITPLSPSHTQQNDTDQVPSGPSFVVVVAIDFGTTSSGYAY

AFAKEPECIHTMRRWEGGDPGVSNQKTPTTILLTPDRKFHSFGYAARDFYHDLDPSESKHWLYLEKFKMK

LHTTANLSINTDLHAANGKRVKALDIFAYALAFFKEQALKELSDQTGGEFDNSDVRWVITVPAIWKMPAK

QFMREAAYKSGLVSRENPEQLIIALEPEAASIYCRKLRLHQMVDLGTQTTQNGFSPSDNVGSGMSQAAKE

HVRRNRQSRTFLVENVIGELWSELEEGDRYVVVDCGGGTVDLTVHQIRLPEGHLKELYKASGGPYGSIGI

DYEFEKLLCKIFGQDFIDQFKIKRPAAWVDLMIAFESRKRAAAPDRTNPLNINLPFSFIDYYKKFRGHSV

EHALRKSNVDFVKWSSQGMLRMSPDAMNSLFKPTIDHIIQHLTELFEKPEVSDIKFLFLVGGFAESPLLQ

QAVQNMLQGRSRIIIPHDVGLTILKGAVLFGLDPSVIKVRRSPLTYGVGVLNRFVEGKH PPEKLLVKDGT

RWCTDVFDTFIAADQSVALGEMVKRSYTPAKPSQQVIVIHVYCSEKENVGFISEPGVR KCGTLRLDVSGT

ESTAARREIQTLMQFGDTEIRAMAVDVSTGRTVKASIDFLSH

>HSPA12B Homo sapiens

MMRKWEGGDPGVAHQKTPTCLLLTPEGAFHSFGYTARDYYHDLDPEEARDWLYFEK FKMKIHSATDLTLK

TQLEAVNGKTMPALEVFAHALRFFREHALQELREQSPSLPEKDTVRWVLTVPAIWKQ PAKQFMREAAYLA

GLVSRENAEQLLIALEPEAASVYCRKLRLHQLLDLSGRAPGGGRLGERRSIDSSFRQA REQLRRSRHSRT

FLVESGVGELWAEMQAGDRYVVADCGGGTVDLTVHQLEQPHGTLKELYKASGGPYG AVGVDLAFEQLLCR

IFGEDFIATFKRQRPAAWVDLTIAFEARKRTAGPHRAGALNISLPFSFIDFYRKQRGHN VETALRRSSVN

FVKWSSQGMLRMSCEAMNELFQPTVSGIIQHIEALLARPEVQGVKLLFLVGGFAESAV LQHAVQAALGAR

GLRVVVPHDVGLTILKGAVLFGQAPGVVRVRRSPLTYGVGVLNRFVPGRHPPEKLLV RDGRRWCTDVFER

FVAAEQSVALGEEVRRSYCPARPGQRRVLINLYCCAAEDARFITDPGVRKCGALSLEL EPADCGQDTAGA

PPGRREIRAAMQFGDTEIKVTAVDVSTNRSVRASIDFLSN

>hspa12b Mus musculus

MLTVPEMGLQGLYISSSPERSPVPSPPGSPRTQESCGIAPLTPSQSPKPEARALQQASFS VVVAIDFGTT

SSGYAFSFASDPEAIHMMRKWEGGDPGVAHQKTPTCLLLTPEGIFHSFGYTARDYYHD LDPEEARDWLYF

EKFKMKIHSATDLTLKTQLEAVNGKKMLALEVFAHALRFFKEHALQELREQSECMLE KGAVRWVLTVPAI

WKQPAKQFMREAAYLAGLVSREDAEKLLIALEPEAASVYCRKLRLHQLMDLSSRTAG

RGRLGERRSIDSS

FRHAREQLRRSRHSRTFLVEAGVGELWAEMQEGDRYMVADCGGGTVDLTVHQLEQP
HGTLKELYKASGGP

YGAVGVDLAFEQLLCRIFGEDFIAKFKRQRPAAWVDLTIAFEARKRTAGPHRAGALNI
SLPFSFIDFYRK

QRGHNVETALRRSSVNLVKWSSQGMLRMSCEAMNELFQPTVSGIIQHIEMLLAKPEV
QGVKLLFLVGGFA

ESAVLQHAVQEALGTRGLRVVVPHDVGLTILKGAVLFGQAPGVVRVRRSPLTYGVGV
LNRFVPGHHPPEK

LLVRDGRRWCTDVFERFVAAEQSVALGEEVRRSYCPARPGQRRVLINLYCCAAEDAR
FITDPGVRKCGAL

SLELEPEGCPENTGTSPSRREIRAAMQFGDTEIKVTAVDVSTNRSVRAAIDFLSN

>hspa12b Danio rerio

MADVLQLSINSLQVPGEDKSDSTSPSGSPFPSRNECSITPLTPSPSPRTEVRPRLARPFY
VVVAIDFGTT

SSGYAFSFIEDPETIHMMRRWEGGDPGVANQKSPTCLLLTPDLRFHSFGFAARDSYHD
LDPEEARHWLYF

DKFKMKIHSTSDLTMETELESVSGRRVQAIEVFAHALRFFREHALKEVKDQSSSVLEG
NEVRWVITVPAV

WRQPAKQFMREAAYLAGLVPPDSPEQLLIALEPEAASIYCRKLRLHQVTDLSQRPVTN
GFDIDGSRPFDS

SFRQAREQLRRARHSRTFLVESGTGELWSEMQTGDRYIVADCGGGTVDLTVHQIEQP
QGTLKELYKASGG

PYGAVGVDLAFETMLCQIFGTDFIDSFKAKRPAAWVDLTIAFEARKRTAAPGRANTLN
ISLPFSFIDFYK

QHRGQSVETALRKSNMNFIKWSSQGMLRLSTEATNELFQPTINNIIKHIENVMQKEEV
KGVRFLFLVGGF

AESPMLQRAIQNTLGRNCRIIIPHDVGLTILKGAVLFGLDPTVVRVRRCPLTYGVGVLN
RFVEGRHPHDK

LLIKDGREWCTDILDRFVSVDQSVALGEVVRRSYTPARMGQRKIIINIYCSDTDDITYIT
DPGVRKCGAI

TLDLLESGEASASTGDNDKGSAFERREIRTTMQFGDTEIKVTAVDVATGRLVRASIDFL
SN

>hspa12b Oryzias latipes

MADEVQPDSSSLQILGERTSIPGSPAIARNDCSITPLTPSPSPRVEIRPRMSCPFSVVVAID
FGTTSSGY

AFSFTQDSEAIHMMKRWEGGDPGVANQKSPTCLLLTPDLRFHSFGFAARDFYHDLDP
EEARHWLYFDKFK

MKIHSTSDLSMETELEAVNGRRVRAIEVFAHALRFFRQHALKEVKDQSSSVLEGEEIR
WVITVPAVWRQP

AKQFMREAAYLAGLISPECPEQLLIALEPEAASIYCRKLRLHQVIDLSLQPITNGLDAD
GSRPFDSSFRQ

AREQLRRSRHSRTFLVESGTGELWSELQTGDKYVVADCGGGTVDLTVHQIEQPQGTL
KELYKASGGPYGA

VGVDLAFENMLCQIFGEDFIQSFKAKRPAAWVDLTIAFEARKRTASPGRANALNISLPF
SFIDFYKRQRG

QSVEAALRRSNMNIVKWSSQGMLRLTQEAMNELFQPTINNIVKHIELLMAKPEVSGV
RFLFLVGGFAESP

MLQRAVQRALGRSCRIIIPHDVGLTILKGAVLFGLDPTVVRVRRSPLTYGVGVLNRFVE
GRHPRDKLLIK

DGREWCTDILDRFVSVDQSVALGEVVRRSYTPARVGQRKIIINIYCSATDDVTYISDPG
VRKCGAITLDL

PEPLPPTGAVGGAGAAASERREIRATMQFGDTEIKVTAVDVISNRSVKASIDFLSN

>hspa12b Gasterosteus aculeatus

MADMLQPDSSSLQLPGDRLSAPSSPATARNDCGITPLTPSPSPMVEVKPRMSHPFSVV
VAIDFGTTSSGY

AFSFTQDSDAIHMMKRWEGGDPGVANQKSPTCLLLTPDLRFHSFGFAARDFYHDLDP
EEARHWLYFDKFK

MKIHSTSDLTMETELEAVNGRRVRAIEVFGHALHFFREHALKEVKDQSSSVLEGEDV
RWVLTVPAVWRQP

AKQFMREAAYLAGLVSRDCPEQLLIALEPEAASIYCRKLRLHQVLDLSLQPIANGFDL
EGSRPFDSSFRQ

AREQLRRSRHSRTFLVESGTGELWSELQTGDRYIVADCGGGTVDLTVHQIEQPQGTLK ELYKASGGPHGA

VGVDLAFEAMLCQIFGEDFILSFKAKRPAAWVDLTIAFEARKRTAAPGRANALNISLPF SFIDFYKRHKG

QSVEAALRRSNMNIVKWSSQGMLRLTQEAMNELFQPTIDKIVKHIEELMAKPEVCGV RFLFLVGGFAESP

MLQRAVQRAVGRTCRIIIPHDVGLTILKGAVLFGLDPTVVRVRRCPLIYGVGVLNRFVE GRHPRDKLLVK

EGREWCTDILDRFVCIDQSVALGEVVRRSYTPARLGQRKIIINIYCSSAEDVAYITDPGV RKCGTITLDL

PEPLPVPGGAGAGGGAGPERREIRATMQFGDTEIKVTAVDVMSNRFVRASIDFLSN

>hspa12b Larimichthys crocea

MADVVQPDPNSLQLPGDNLSAPSSPATARNDCSITPLTPSPSPRVEVRPRMSCPFSVVV AIDFGTTSSGY

AFSFTQDSEAIHMMKRWEGGDPGVANQKSPTCLLLTPDLRFHSFGFAARDFYHDLDP EEARHWLYFDKFK

MKIHSTSDLTMETELEAVNGRRVRAIEVFAHALHFFREHALKEVKDQSSSVLEGEEIR WVITVPAVWRQP

AKQFMREAAYLAGLVSPDCPEQLLIALEPEAASIYCRKLRLHQVIDLSLQPITNGLDLE GSRPFDSSFRQ

AREQLRRSRHSRTFLVESGTGELWSELQTGDRYVVADCGGGTVDLTVHQIEQPQGTL KELYKASGGPYGA

VGVDLAFEAMLCQIFGEDFIQSFKAKRPAAWVDLTIAFEARKRTAAPGRANALNISLP FSFIDYYKRHRG

QSVEAALRRSNMNIVKWSSQGMLRLTQEAMNELFQPTIINIVKHIEELMVKPEVRGV RFLFLVGGFAESP

MLQKAVQRALGRTCRIIIPHDVGLTILKGAVLFGLDPTVVRVRRCPLTYGVGVLNRFV EGRHPRDKLLIK

EGREWCTDILDRFVSVDQSVALGEVVKRSYTPARLGQRKIIINIYCSTTDDVTYISDPG VRKCGTITLDL

PEPLPLPGAVGGAGAGGGPERREIRATMQFGDTEIKVTAVDVMSNRSVRASIDFLSN

>hspa12b Oreochromis niloticus

MADVLQPDSNSLQIPSENVSTPSSPATARNDCSITPLTPSPSPRVEVRPRMACPFSVVIAI
DFGTTSSGY

AFSFTQDSEAIHMMKRWEGGDPGVANQKSPTCLLLTPELRFHSFGFAARDFYHDLDP
EEARHWLYFDKFK

MKIHSTSDLTMETELEAVNGQKVRAIDVFAHALRFFREHALKEVKDQSSSVLEGDEIR
WVITVPAVWRQP

AKQFMREAAYLAGLVSPDCPEQLLIALEPEAASIYCRKLRLHQVIDLSMQPITNGLEL
DVSRPFDSSFRQ

AREQLRRSRHSRTFLVESGTGELWSELQTGDRYVVADCGGGTVDLTVHQIEQPQGTL
KELYKASGGPYGA

VGVDLAFEAMLCQIFGEDFIQSFKAKRPAAWVDLMIAFEARKRTASPGRTNALNISLP
FSFIDYYKRHRG

QSVEAALRRSNMNIVKWSSQGMLRLTQEAMNELFQPTISKIVKHIEELMTKPEVRGV
RFLFLVGGFAESP

MLQKAVQKALGRTCRIIIPHDVGLTILKGAVLFGLDPTVVRVRRCPLTYGVGVLNRFV
EGRHPRDKLLIK

DGREWCTDILDRFVSVDQSVALGEVVRRSYTPARLGQRKIIINIYCTATDDVTYISDPG
VRKCGTITLDL

PEPLPPPGAVGGAGAGTPERREIRATMQFGDTEIKVTAVDVMSNRSVRASIDFLSN

>HSPA13 Homo sapiens

MAREMTILGSAVLTLLLAGYLAQQYLPLPTPKVIGIDLGTTYCSVGVFFPGTGKVKVI
PDENGHISIPSM

VSFTDNDVYVGYESVELADSNPQNTIYDAKRFIGKIFTAEELEAEIGRYPFKVLNKNG
MVEFSVTSNETI

TVSPEYVGSRLLLKLKEMAEAYLGMPVANAVISVPAEFDLKQRNSTIEAANLAGLKIL
RVINEPTAAAMA

YGLHKADVFHVLVIDLGGGTLDVSLLNKQGGMFLTRAMSGNNKLGGQDFNQRLLQ
YLYKQIYQTYGFVPS

RKEEIHRLRQAVEMVKLNLTLHQSAQLSVLLTVEEQDRKEPHSSDTELPKDKLSSADD
HRVNSGFGRGLS

DKKSGESQVLFETEISRKLFDTLNEDLFQKILVPIQQVLKEGHLEKTEIDEVVLVGGST RIPRIRQVIQE

FFGKDPNTSVDPDLAVVTGVAIQAGIDGGSWPLQVSALEIPNKHLQKTNFN

>hspa13 Mus musculus

MAGEMTILGSAVLTLLLAGYLAQQYLPLPTPKVIGIDLGTTYCSVGVFFPGTGKVKVI PDENGHISIPSM

VSFTDGDVYVGYESLELADSNPQNTIYDAKRFIGKIFTPEELEAEVGRYPFKVLHRNG MAEFSVTSNETI

IVSPEFVGSRLLLKLKEMAEEYLGMPVANAVISVPAEFDLQQRNSTIQAANLAGLKILR VINEPTAAAMA

YGLHKVDVFYVLVIDLGGGTLDVSLLNKQGGMFLTRAMSGNNKLGGQDFNQRLLQ HLYKEIYQTYGFLPS

RKEEIHRLRQAVEMVKLNLTIHQSAQVSVLLTVEGKDSKEPQNGDSELPKDQLTPGD GHHVNRVFRPGLS

ESKSGKSQVLFETEVSRKLFDALNEDLFQKILVPIQQVLKEGLLDKTEIDEVVLVGGST RIPRIRQVIQE

FFGKDPNTSVDPDLAVVTGVAIQAGIDGGSWPLQVSALEIPNKHLQKTNFN

>hspa13 Gallus gallus

MAGQMAVLGSAVLALLLAGYLAQQYLPMPTPKVIGIDLGTTYCSVGVFLPGTGQVK VIADENGHNSIPSI

VSFTDRHVYVGYDGLELADANPQNTIYDAKRFIGKIFTSEELKSESSRYPFKIFNNDGS AEFSVTTNETF

RVSPEHIGSQLLLKLKRMAEDHLGVPILKAVISVPAEFDERQRNATVKAANLAGLEILR VINEPTAAAMA

YGLHKADVFNVLVVDLGGGTLDVSLLNKQGGMFLTRAMAGNNKLGGQDFNQRLM QHLYDQLNQVYGSLPS

RKEEIHRLRQAVEAVKLNLTIHEASTLRVSLTMPERKLTKELPESEVKLNTIHNSKPSQ KTEDLKNRGDA

SDEENNVVKVVFEREISRKLFEMLNEDLFKKILVPIEQVLKEGHLRKEEVDEIVLVGGS TRIPKIREVIR

DFFGKEPNTSVDPDLAVVMGVAIQAGIVGGSWPLQVSAIEIPNKHLRKTNFN

>hspa13 Danio rerio

MAGEMSMIGSVILALLLAGYLGQQYLPPPKPRVIGLDLGTTFCSVGVFQPGTGEIEIIE DDKGRKSIPSV

VSFTLTGVFAGHEGQELSDVNPQNTIYDAKRFIGKIFDEETLEKESARYPFKVIFNNGS ADFLVNTNSTF

TVTPEFIGSRLLLKMRKMAEKQLGVPVEKAVISVPAEFDERQRNYTIRAANLAGLDVL RVINEPTAAAMA

YGLHKAEVFNVLVVDLGGGTLDVSLLNKQGGMFLTRAMAGNNQLGGQDFTQRLLQ YTTERVRQQYGVPPT

LKEDIHLLRQAVEAAKLNLTQEPHVHLRVPLYLQMTGASGAQEEKVLFEEKLTRETFE ELNADLFQKILA

PIETVLVEGHLDKQEVDEIVLVGGSTRIPRIRQLISQYFGKEPNTSVDPDLAVVTGVAIQ AGIMGGSWPL

QVSAIEIPNRHLRKTNFS

>hspa13 Oryzias latipes

MSGELSMIGSVILALFLAGYLGQQYLPPPKPKVIGLDLGTTFCSVGVFHPGTGEVEVI ADEQGRKSIPSA

VSFTATEVLAGHEALELADSNAHNTFYDAKRFIGKLFEPGLLEQESARYPFKVINNNG SAEFVVFTNRSF

TVTPEFIGSRLLLKMKKMAELQLGVPIQKAVISVPAEFDERQRNYTQRAANLAGLEIL RVINEPTAAAMA

YGLHKVDVFNVLVVDLGGGTLDVSLLNKQGGMFLTRAMAGNNKLGGQDFSQKLLQ YTTERVRQEFGVVPT

LKEDIHHLRQAVEAAKLNLTLHATATVSVPLHLHSHQASGGPRTSAVLFRTVITRALFE ELNEELFQKIL

APIKTVLAEGHLGKEDVDEIVLVGGSTRIPRIREIIRQFFGKEPNTSVDPDLAVVTGVAI QAGIMGGSWP

LQVSAVEIPNRHLRKTNFS

>hspa13 Gasterosteus aculeatus

MSGEISMIGSVILALFLAGYLGQQYLPPPKPKVIGLDLGTTFCSVGVFHPGSGEVEVIA D

EEGRRSIPSAVSFTATAVLAGHEALELADDNPQNTVYDAKRFIGKIFEPGVLEQESARYP

FKVINNNGSAEFLLSTNHTFTVSPEFIGSRLLLKMRKMAERHLDARIQKAVISVPAEFDE

RQRNFTVRAANLAGLEILRVINEPTAAAMAYGLHKVDVFNVLVIDLGGGTLDVSLLNKQG

GMFFTRAMAAGNNKLGGQDFSQRLLQYTIERARQEFGVPPALKEDIHRLRQAVEAAKLNL

TLQPSAAVSVALRLRGPDGSEAAAPVPFRAVITRELFEELNEDLFQKILAPVETVLAEGG

LEKVDVDEIVLVGGSTRIPRIRRLISEYFGKDPNTSVDPDLAVVTGVALQAGIMGGSWPL

QVSAIEIPNRHLLKRNFS

>hspa13 Larimichthys crocea

MSGEISMIGSVILALFLAGYLGQQYLPPPKPKVIGLDLGTTFCSVGVFHPGSGEVEVIADEEGRKSIPSA

VSFTTAAVLAGHEAVDLADSNPQNTVYDAKRFIGKIFEPEVLEQESARYPFKVINNNGSAEFLVSTNHTF

TVSPEFIGSRLLLKMRKMAERQLGMPIQKAVISVPAEFDERQRNYTIRAANLAGLEILRVINEPTAAAMA

YGLHKVDVFNVLVVDLGGGTLDVSLLNKQGGMFLTRAMAGNNKLGGQDFSQRLLQYTTDRVRQEFGVPPT

LKEDIHRLRQAVEAAKLNLTLHPSVDVRVPLHLHTHDGSESPEGSAPAKVLFQAVITRKLFEELNEDLFQ

KILAPVETVLTEGHLEKEDVDEIVLVGGSTRIPRIRKLISEYFGKEPNTSVDPDLAVVTGVAIQAGIMGG

SWPLQVSAIEIPNRHLRKTNFS

>hspa13 Oreochromis niloticus

MAGEVSMIGSVILALFLAGYLGQQYLPPPKPKVIGLDLGTTFCSVGVFHPGSGEVEVIADEEGRKSIPSA

VSFTTTAVLAGHEAVDLADINPQNTIYDAKRFIGKIFEPEVLEKESARYPFKVINNNGS

AEFVISTNHTF

TVTPEFIGSRLLLKLRKLAERQLGVVVQKAVISVPAEFDERQRNYTARAANLAGLEVL
RVINEPTAAAMA

YGLHKVDVFNVLVVDLGGGTLDVSLLNKQGGMFLTRAMAGNNKLGGQDFSQRLLQ
YTTERVQQEFGVPPT

LKEDIHHLRQAVEAAKLNLTLQPSVTIQVPLHLRTLDRFDSSTPPPVLFQAVITRTLFEE
LNEDLFQKIL

APVETVLAEGHLDKKEVDEIVLVGGSTRIPRIRRLISEYFGKEPNTSVDPDLAVVTGVA
IQAGIMGGSWP

LQVSAIEIPNRHLRKTNFS

>HSPA14 Homo sapiens

MAAIGVHLGCTSACVAVYKDGRAGVVANDAGDRVTPAVVAYSENEEIVGLAAKQSRI
RNISNTVMKVKQI

LGRSSSDPQAQKYIAESKCLVIEKNGKLRYEIDTGEETKFVNPEDVARLIFSKMKETAH
SVLGSDANDVV

ITVPFDFGEKQKNALGEAARAAGFNVLRLIHEPSAALLAYGIGQDSPTGKSNILVFKL
GGTSLSLSVMEV

NSGIYRVLSTNTDDNIGGAHFTETLAQYLASEFQRSFKHDVRGNARAMMKLTNSAEV
AKHSLSTLGSANC

FLDSLYEGQDFDCNVSRARFELLCSPLFNKCIEAIRGLLDQNGFTADDINKVVLCGGSS
RIPKLQQLIKD

LFPAVELLNSIPPDEVIPIGAAIEAGILIGKENLLVEDSLMIECSARDILVKGVDESGASR
FTVLFPSGT

PLPARRQHTLQAPGSISSVCLELYESDGKNSAKEETKFAQVVLQDLDKKENGLRDILA
VLTMKRDGSLHV

TCTDQETGKCEAISIEIAS

>hspa14 Mus musculus

MAGPMWLQMMQGIESHQPLLLTRNVNSSADPQAQKYISESKCLVIEKNGKLRYEIDT
GEETKLVNPEDVA

RLIFSKMKETAHSVLGSDANDVVVTVPFDFGEKQKSALGEAAGAAGFNVLRLIHEPS
AALLAYGIGQDHP

TGKSNVLVFKLGGTSLSLSVMEVNSGMYRVLSTNTSDNIGGAHFTDTLAQYLASEFQ
RLFKHDVRGNARA

MMKLMNSAEVAKHSLSTLGSANCFVDSLYEGQDFDCNVSRARFELLCSPLFNKCTEA
IRELLRQTGFTAD

DINKVVLCGGSSRIPKLQQLIKDLFPAVDLLNSIPPDEVIPIGAAIEAGILVGKESTSGDD
SVMIECSAK

DILVKGVDESGADRFTVLFPSGTPLPARRQHTLQAPGRVSSVCLELYESEGKNSAKEE
AKFAQVVLQDLD

KKENGLRDILAVLTMKRDGSLQVTCTDQDTGKCEAITVEVAS

>hspa14 Gallus gallus

MAAIGVHLGATCACAAVYKDGRADVVANDAGDRVTPAVVAFTENEEVVGLAAKQS
RIRNVSNTVVKVKQI

LGRSSGDPQAEKYIKESKCLVVEKNGKLQYEIDNKLISPEDVAKLIFSKMKETAQSAL
GSDVNDVVITVP

FDFGENQKNALGEAAAAAGFNVMRLIHEPSAALLAYGIGQDSPTGKSNVLVYKLGG
TSLSITVIEVNSGI

YRVLATDTDDGIGGVYFTEALAQHLASEFQRSCKHDIRGNPRAMMKLMNSADVAKH
SLSTLGSANCFVDS

LYDGLDFDCNVSRARFELICSSLFSKCVEAIKKLLEQVGFTAEDINKVVLCGGSARIPK
LQQLIKDIFPN

VELLSSIPPDEVIPIGAAIEAGILLGKENPSLEEEALFIECSAKDILLKGVDETGADKFTV
LFPSGTPLP

ARRQHTLHAPGSTSSVCLELYESLGKSPMNEENKFAQIVLQDLDKKDGLHDILTVLTM
KRDGSLHVTCTD

QDTGKCEAITVEVAS

>hspa14 Danio rerio

MAAIGVHFGYTCACVAVFKDGRADVVANDAGDRVTPAVVAYRDTEQIVGIAAKQGRI
RNAANTVVKVKQI

LGRRYDDPDAQAHKEESKCIVVNKSGLPRYEIDTGETTKYVSPEDVAKLIFHKMKETA
QSALGSDVKDAV

ITVPFEFDEMQKNALRQAAESAGFNVLRLIHEPSAALLAYDIGQDSPLGKSHVLVYKL

GGTSLSVTVLEV

NSGVYRVLATQTDHQTGGESFTQELAQHLAAEFKKTFKQDVSGNARAMMKLMNSA
DVAKHTLSTLGSANC

FVDSLYDGMDFECNVSRARFELICSSLFNKCIQPIKSLLEQVNLSTSDVNKVVLSGGSA
RIPKLQQMIRD

LFPDVELLNSIPPDEVIPVGAAMQAGILVGKDSLALGEDSITVDCCASDITLKEVDDSG
LEVFTVLFPSG

TPLPARRQHTLQGPGSLSSVRLQLFQAQQPIAQIVLGDLEPKEELHDVVTVLTMKRDG
SLHVTCTEQSSG

RSEAITIETAAAAS

>hspa14 Oryzias latipes

MSAIGVHFGYTCACVAIFKDGRADVVANDAGDRVTPAVVGFRDTEQIVGLAAKQGRI
RNAANTVVKVKQM

LGRRFDDPETKVHQTETKCQVVSKDGKPYYEITVGQQPQHVAPEDVAKLIFSKMRET
AQSALGSDTSEAV

ITVPFEFAHAQRRALREAAEAAGFRVLRMIHEPAAALLAYDVGQDSSSGKSHVLVYK
LGGTSLSVTVLQI

NGGIFRVLSTKTDHGIGGESLTQALAQHLASEFKRVYKHDVSSNARAMLKLMISADM
TKHSLSSLGSANC

FVDSLHDGIDFECNVSRARFELLCSSLFNKSIQPIRSVLAEAGLSTSDINKVALCGGSAR
IPRLQQLIRE

MFPDVELLNSAPADEVIAVGAALEAGLLVGREGLAPEEESVTVDVSAADILLKEVDEA
GAETFTLLLPSG

TPLPARRHHSLPADGQLSSLCLEIYQRFSSERPDLLSKMILRELQPVQDNRCIDAVVTM
KRDGSLHVSCV

DRSTGRSEAVTIAAAAV

>hspa14 Gasterosteus aculeatus

MAAIGVHFGYTCACAAIFKDGRAEVVANDAGDRVTPAVVGYRDTEKIVGIAAKQGRI
RNAANTIVKVKQV

LGRSFDDPETQSHKTQTKCQVVNRQEKPYYEITAGDHPEYVAPEDVAKLIFHKMKET
AQSALGSDVTEAV

ITVPFEFAHAQKRALREAAEAAGFHVLRLIHEPAAALLAYNIGQDCSSGKSHVLVYKL
GGTSLSVTVLQV

NGGVFRVLNTHTDHSTGGESFTQALAQHLNAEFKRTYKYDVSTNVRAMLKLMNGA
DMAKHSLSSLESANC

FVDSLHDGIDFECNVSRARFELLCSSLFNKSIQPIRPLLEAAGLSTADINKVVLCGGSA
RIPRLQQMIRE

MFPDVELLSSSPPDEVIAVGAALEAGLLVGKDSLAPEEDSITVDVSATDILVKEVDESG
AEAFTILFPSG

TPLPARRHHVLSAQGGLSSLCLEIYQRFVTEQPEKLAKIVLRDLQPGTENHNIDTVVT
MKRDGSVHVSCV

EQSCGRPEVVTIAAAS

>hspa14 Larimichthys crocea

MCACVVHPLALRLFSMAAIGVHFGYTCACAAIFKDGRADVVANDAGDRVTPAVVAY
RDTEQIVGIAAKQG

RVRNAANTVVKVKQVLGRSFDDPETQAHKAQTKCQVVNKEEKPYYEITTGEHPEYV
APEDAAKLIFHKMK

ETAQSALGSDVTEAVITVPFEFAHAQKRALREAAEAAGFHVLRLIHEPAAALLAYNIG
QDCSSGRSHVLV

YKLGGTSLSVTVLQVSGGIFRVLNTHTDHSIGGESFTQALAQYLATEFKRTFKHDVSS
NARAMLKLMNGA

DMAKHSLSSLGSANCFVDSLHDGIDFECNVSSNMER

>hspa14 Oreochromis niloticus

MSAIGVHFGYTSACVAIFKDGRADVVANDAGDRVTPTVVGYRDTEQIVGIAAKQGRV
RNAASTVIKVKQV

LGRSFDDPEIQRHRTETKCQVVSRENKPFYEITSGQQPKYVAPEDVAKLIFQKMKETA
QSALGGSDVSDA

VITVPSEFAHAQKRALRNAAEAAGFNVLRLIHEPAAALLAYNIGQESPSGKSHVLVYK
LGGTSLSVTVLQ

VNGGMFLCRSTHTDHSIGGEAFTQALAQHLASEFKRTFKHDVSSNPRAMLKLMNGA
EMAKHSLSSLGSAN

CFVDSLHDGIDFECSVSRARFELLCSSLFNKSIQPIKAVLEEAGLTPHSISKVVLCGGSA

RIPRLQQMIR

EMFPDVELLNSAPPDEVIAVGAALEAGLLVGRDGLTPEEDSVTVDVSSIDVLVKEMDE
SGAEMFTVLLPV

GTPLPAHRHHILRGDGKLSSLCLEIYQRCITEQPEKLSKIVFRDLQPTEEAHSIDTVVTM
KSDGSIHLSC

VEQGTGRSEVVTIAAAS

>HSPH1 Homo sapiens

MSVVGLDVGSQSCYIAVARAGGIETIANEFSDRCTPSVISFGSKNRTIGVAAKNQQITH
ANNTVSNFKRF

HGRAFNDPFIQKEKENLSYDLVPLKNGGVGIKVMYMGEEHLFSVEQITAMLLTKLKE
TAENSLKKPVTDC

VISVLGTAFDPFLGGKNFDEKLVEHFCAEFKTKYKLDAKSKIRALLRLYQECEKLKKL
MSSNSTDLPLNI

ECFMNDKDVSGKMNRSQFEELCAELLQKIEVPLYSLLEQTHLKVEDVSAVEIVGGAT
RIPAVKERIAKFF

GKDISTTLNADEAVARGCALQCAILSPAFKVREFSVTDAVPFPISLIWNHDSEDTEGVH
EVFSRNHAAPF

SKVLTFLRRGPFELEAFYSDPQGVPYPEAKIGRFVVQNVSAQKDGEKSRVKVKVRVN
THGIFTISTASMV

EKVPTEENEMSSEADMECLNQRPPENPDTDANEKKVDQPPEAKKPKIKVVNVELPIE
ANLVWQLGKDLLN

MYIETEGKMIMQDKLEKERNDAKNAVEEYVYEFRDKLCGPYEKFICEQDHQNFLRL
LTETEDWLYEEGED

QAKQAYVDKLEELMKIGTPVKVRFQEAEERPKMFEELGQRLQHYAKIAADFRNKDE
KYNHIDESEMKKVE

KSVNEVMEWMNNVMNAQAKKSLDQDPVVRAQEIKTKIKELNNTCEPVVTQPKPKIE
SPKLERTPNGPNID

KKEEDLEDKNNFGAEPPHQNGECYPNEKNSVNMDLD

>hsph1 Mus musculus

MSVVGLDVGSQSCYIAVARAGGIETIANEFSDRCTPSVISFGSKNRTIGVAAKNQQITH
ANNTVSSFKRF

HGRAFNDPFIQKEKENLSYDLVPMKNGGVGIKVMYMDEEHFFSVEQITAMLLTKLKETAENNLKKPVTDC

VISVPSFFTDAERRSVLDAAQIVGLNCLRLMNDMTAVALNYGIYKQDLPNAEEKPRVVVFVDMGHSSFQV

SACAFNKGKLKVLGTAFDPFLGGKNFDEKLVEHFCAEFKTKYKLDAKSKIRALLRLHQECEKLKKLMSSN

STDLPLNIECFMNDKDVSGKMNRSQFEELCAELLQKIEVPLHSLMAQTQLKAEDVSAIEIVGGATRIPAV

KERIAKFFGKDVSTTLNADEAVARGCALQCAILSPAFKVREFSVTDAVPFPISLVWNHDSEETEGVHEVF

SRNHAAPFSKVLTFLRRGPFELEAFYSDPQGVPYPEAKIGRFVVQNVSAQKDGEKSRVKVKVRVNTHGIF

TISTASMVEKVPTEEEDGSSLEADMECPNQRPTESSDVDKNIQQDNSEAGTQPQVQTDGQQTSQSPPSPE

LTSEESKTPDADKANEKKVDQPPEAKKPKIKVVNVELPVEANLVWQLGRDLLNMYIETEGKMIMQDKLEK

ERNDAKNAVEECVYEFRDKLCGPYEKFICEQEHEKFLRLLTETEDWLYEEGEDQAKQAYIDKLEELMKMG

TPVKVRFQEAEERPKVLEELGQRLQHYAKIAADFRGKDEKYNHIDESEMKKVEKSVNEVMEWMNNVMNAQ

AKRSLDQDPVVRTHEIRAKVKELNNVCEPVVTQPKPKIESPKLERTPNGPNIDKKEDLEGKNNLGAEAPH

QNGECHPNEKGSVNMDLD

>hsph1 Gallus gallus

MAVVGFDLGFQSCYIAVARAGGIETVANEFSDRCTPSVVSFGSKNRAIGVSAKNQQITHAHNTVSNFKRF

HGRAFNDPFVQKEKEKLSYDLVPMKNGGVGVKVMYMDEEHIFSVEQISAMLLTKLKETAESNLKKPVTDC

VISVPSFFTDAERRSVLDAAQIVGLNCLRLMNDMTAVALNYGIYKQDLPAPEEKPRIVVFVDMGHSAFQV

SACAFNKSKLKVLGTAFDPFLGGRNFDGKLVDYFCAEIKAKYKLDPKSKVRALLRLY

QECEKLKKLMSSN

STDIPLNIECFMNDTDVSGKMNRSQFEELCADLLQRIEMPLLSLMEQTQLKVEDVTAV
EIVGGATRIPAV

KERIAKFFGKDVSTTLNADEAIARGCALQCAILSPAFKVREFSVTDATPFPISLLWNTE
AEDTEGVHEVF

SRNHAAPFSKVLTFYRKGPFELEAFYSDPNGVPYPESKIGRYIIQNVAAQKDGEKSKV
KVKVRVNTHGIF

SVSTASMVEPVKSEDSEDVGVETELETQDQMPAENSSDKNNQQENSEAGTQSQVQT
DGQQTSQSPPSSEP

PSEENKIPDVKKTSEKKGDQPPEAKKPKIKVKNVELPIEANLVWQLGKDLLNMYIETE
GKMIMQDKLEKE

RNDAKNAVEEYVYEFRDKLSGPYEKFVCEKDLQGFSALLTETEGWLYEEGEDEAKQ
VYVDKLEDLKKLGT

PIEMRYQEAEERPKLLEELGHRLQYYAAIAGEFRNKDEKYIHIDEMEMMKVEKCVSE
VIEWMNNAVSAQA

KKSLDQDPAVRSFEIKAKLQELNNVCEPIVTQPKPKVDSPKEENPLNEQGDYKTEDM
GEDDKNSDMPQQN

GECHPGDQNTVNMDLD

>hsph1 Danio rerio

MNDSTAVALNYGIYKEDLPGSDENPKIVAFVDLGHSAFQVSICAFNRGKVKVLSTAFD
PYLGGKDFDQRL

VDHFCAEFKSKYMMDVKSKTRALLRLTQECEKLKKLMSSNSTELSLNIECFIDDKDV
CGRMNRAKFEEMC

ADLIERVKFTLMKAVEQAGVRLQDISAVEIVGGATRIPAVKAQISSFFRRDVSTTLNAD
EAVARGCALQC

AMLSPAFRVREFSITDVIPFPISLSWSSEADEGKSCHEIFSRNHPCPSAKMITFYRNKPFI
LEAFYSDKS

SLPFPEAKIGEYKVQNIQPQENREKAKVKVKVEVNRSGVVSVSSAAVVLRVRSDESEI
TEINEDVDSHDA

SDDTDVQDKTEEVNIELSVNKENLPTAQCPPMEKEQNQRETLSMNGEVNSHPPHAKK
AKMKVKHVVLPME

ETFNQQLPKDRLSAYTEQENKMIQQDRQEKERNNAKNAVEENVYYFRHKLEGSYQT
FLNAQEHQAFSELL

NGTENWLYDEGADQDKQTYINRLAEIHKLGLPVENRYRESIRRPRMFEELSAKIQSY
MTIVEDYKNGSES

YCHIDAMDMDKVRVCVKDTQVWMTNIQDSQDKLTPDQEPAIHSTQIQKKLQTLNNV
CGEIVSKPKPRVDS

PMDDTIQPERLHKHKSPEDVTMDYTDFVQNERKNNHVNI

>HYOU1 Homo sapiens

MADKVRRQRPRRRVCWALVAVLLADLLALSDTLAVMSVDLGSESMKVAIVKPGVPM
EIVLNKESRRKTPV

IVTLKENERFFGDSAASMAIKNPKATLRYFQHLLGKQADNPHVALYQARFPEHELTFD
PQRQTVHFQISS

QLQFSPEEVLGMVLNYSRSLAEDFAEQPIKDAVITVPVFFNQAERRAVLQAARMAGL
KVLQLINDNTATA

LSYGVFRRKDINTTAQNIMFYDMGSGSTVCTIVTYQMVKTKEAGMQPQLQIRGVGF
DRTLGGLEMELRLR

ERLAGLFNEQRKGQRAKDVRENPRAMAKLLREANRLKTVLSANADHMAQIEGLMD
DVDFKAKVTRVEFEE

LCADLFERVPGPVQQALQSAEMSLDEIEQVILVGGATRVPRVQEVLLKAVGKEELGKN
INADEAAAMGAV

YQAAALSKAFKVKPFVVRDAVVYPILVEFTREVEEEPGIHSLKHNKRVLFSRMGPYPQ
RKVITFNRYSHD

FNFHINYGDLGFLGPEDLRVFGSQNLTTVKLKGVGDSFKKYPDYESKGIKAHFNLDES
GVLSLDRVESVF

ETLVEDSAEEESTLTKLGNTISSLFGGGTTPDAKENGTDTVQEEEESPAEGSKDEPGEQ
VELKEEAEAPV

EDGSQPPPPEPKGDATPEGEKATEKENGDKSEAQKPSEKAEAGPEGVAPAPEGEKKQK
PARKRRMVEEIG

VELVVLDLPDLPEDKLAQSVQKLQDLTLRDLEKQEREKAANSLEAFIFETQDKLYQPE
YQEVSTEEQREE

ISGKLSAASTWLEDEGVGATTVMLKEKLAELRKLCQGLFFRVEERKKWPERLSALDN

LLNHSSMFLKGAR

LIPEMDQIFTEVEMTTLEKVINETWAWKNATLAEQAKLPATEKPVLLSKDIEAKMMA LDREVQYLLNKAK

FTKPRPRPKDKNGTRAEPPLNASASDQGEKVIPPAGQTEDAEPISEPEKVETAGSEPGD TEPLELGGPGA

EPEQKEQSTGQKRPLKNDEL

>hyou1 Mus musculus

MAATVRRQRPRRLLCWALVAVLLADLLALSDTLAVMSVDLGSESMKVAIVKPGVPM EIVLNKESRRKTPV

TVTLKENERFLGDSAAGMAIKNPKATLRYFQHLLGKQADNPHVALYRSRFPEHELIV DPQRQTVRFQISP

QLQFSPEEVLGMVLNYSRSLAEDFAEQPIKDAVITVPAFFNQAERRAVLQAARMAGL KVLQLINDNTATA

LSYGVFRRKDINSTAQNVMFYDMGSGSTVCTIVTYQTVKTKEAGMQPQLQIRGVGF DRTLGGLEMELRLR

EHLAKLFNEQRKGQKAKDVRENPRAMAKLLREANRLKTVLSANADHMAQIEGLMD DVDFKAKVTRVEFEE

LCADLFDRVPGPVQQALQSAEMSLDQIEQVILVGGATRVPKVQEVLLKAVGKEELGK NINADEAAAMGAV

YQAAALSKAFKVKPFVVRDAVIYPILVEFTREVEEEPGLRSLKHNKRVLFSRMGPYPQ RKVITFNRYSHD

FNFHINYGDLGFLGPEDLRVFGSQNLTTVKLKGVGESFKKYPDYESKGIKAHFNLDES GVLSLDRVESVF

ETLVEDSPEEESTLTKLGNTISSLFGGGTSSDAKENGTDAVQEEEESPAEGSKDEPAEQ GELKEEAEPPA

EETSQPPPSEPKGDAAREGEKPDEKESGDKPEAQPNEKGQAGPEGAAPAPEEDKKPK PARKQKMVEEIGV

ELAVLDLPDLPEDELARSVQKLEELTLRDLEKQEREKAANSLEAFIFETQDKLYQPEY QEVSTEEQREEI

SGKLSATSTWLEDEGFGATTVMLKDKLAELRKLCQGLFFRVEERRKWPERLSALDNL LNHSSIFLKGARL

IPEMDQVFTEVEMTTLEKVINDTWAWKNATLAEQAKLPATEKPVLLSKDIEAKMMAL DREVQYLLNKAKF

TKPRPRPKDKNGTRAEPPLNASAGDQEEKVIPPAGQTEEAKPILEPDKEETGTEPADSE PLELGGPGAGP

EQEEQSAGQKRPSKNDEL

>hyou1 Gallus gallus

MARAPRWMLGWLLLACCVPHTEPLAVMSVDMGSESMKIAIVKPGVPMEIVLNKESR RKTPVAVALKENER

LFGDSALGMSIKTPKVAFRYFQDLLGKQIDNPQVALYQSRFPEHELVKDEKRQTVIFK LSQTLQYSPEEM

LGMVLNYSRGLAEEFAEQPIKDAVITVPAYFNQAERRAVLHAARMADLKVLQLINDN TAVALNYGVFRRK

DINATAQNIMFYDMGAGSTVCTIVTYQTVKTKDSGTQPQLQIQGIGFDRTLGGLEMEL RLRDYLAKLFND

QHPSKDVRKNPRAMAKLLKEANRLKTVLSANADHMAQIEGLLDDIDFKAKVSRQEF EDLCSDLFQRVPGP

VQQALSSAEMNLDGIDQVILVGGATRVPKVQEVLLKAVGKEELGKNINADEAAAMG AVYQAAALSKAFKV

KPFMVRDAAMFPIQVEFTREVEEDDKSKSLKHNKRILFQRMAPYPQRKVITFNRYTD DFEFYVNYGDLSF

LNQDDLRIFGSLNLTTVRLKGVGESFKKHSDYESKGIKAHFNMDESGVLSLDRVESVF ETLVEDKLEEES

TLTKLGNTISSLFGGGGHTPEAGENLTDSVQEEEESLAEAAKEEQGVKQGQKSSAED AGEEQGEEKQQSP

HPDQAEAVPPKEESQKNEEGEKSEARDPKEDKETVKEEELSKSSGAGTAAKAEEEKKI KAPKKQKLVHEI

TMELDVNDVPDLLEDELKSSMKKLQDLTIRDLEKQEREKSANSLESFIFETQDKLYQE EYLFVSTEEERE

EISKKLSEASNWMEEEGYAAATKELKDKLAELKKLCRNLFFRVEERRKWPERLAALE SLLNHSNIFLKGA

RMIPESDQIFTEVELGTLEKAINETTVWKNETLAEQNKLSPAEKPVLLSKDIELKIAGL

DREVQYLLNKA

KFAKPKPKKEKNATKSDSGKNATGTSESENTIPPTEGKQEEKPEDISPAKEPPTTEKVV
TDDEPGSDSSS

KKEKKPEAGGESRKNDEL

>hyou1 Danio rerio

MREKLSLWAIFCLVVAFLPSQTESVAVMSVDLGSEWMKVAIVKPGVPMEIVLNKESRR
KTPVAVCLKENE

RLFGDGALGVAVKNPKVVYRFLQSILGKTADNPQVAEYQKHFPEHQLQKDEKRGTV
YFKFSEEMQYTPEE

LLGMILNYSRTLAQDFAEQPIKDAVITVPAYFNQAERRAVLQAAHIAGLKVLQLINDN
TAVALNYGVFRR

KDINSTAQNIMFYDMGSGSTTATIVTYQTVKTKESGTQPQLQIRGVGFDRTLGGFEME
LRLRDHLAKLFN

EQKKSKKDVRDNLRAMAKLLKEAQRLKTVLSANAEHTAQIEGLMDDIDFKAKVTRS
EFEALCEDLFDRVP

GPVKQALAAAEMSMDEIEQVILVGGATRVPKVQDVLLKSVGKEELSKNINADEAAA
MGAVYQAAALSKAF

KVKPFLVRDAAVFPIQVEFSRETEEEDGVKSLKHNKRILFQRMAPYPQRKVITFNRYID
DFVFYINYGDL

SFLSEQDMKVFGSQNLTTVKLSGVGSSFKKHSDAESKGIKAHFNMDESGVLILDRVES
VFETIVEEKEEE

STLTKLGNTISSLFGGGSSEPSANVTEPVTDEEEVTPEAGKEQDQPEKQEETVQEKPET
EEGKEAEPQAE

EQKEDKEKAENQGETESEKTEKPEEKTTDEEKEADMKPKLQKKSKISADIAVELEVN
DVLDPSAEDMEGS

KKKLQDLTDRDLEKQEREKTLNSLEAFIFETQDKLYQDEYQAVVTEEEKEQISGRLSV
ASSWMDEEGYRA

GTKLLKEKLSELKKLCKGMFFRVEERKKWPDRLAALDSMLNHSNIFLKSARLIPESD
QIFTDVELKTLEK

VINETITWKNETVAEQEKLSPTVKPVLLSKDIEAKLSLLDREVNYLLNKAKFAKPKPK
DKAKDKNSTSES

SKANSTDDAEKVIPPKTEDGAEKVKPAEEPPVVEEKAEETILELNPAENTDDKTESTES SKSENHIEDEL

>hyou1 Oryzias latipes

MAAGSLAVVALSCLFLASLPSYSVSVAVMSVDLGSEWIKVAIVKPGVPMEIVLNKESR RKTPTAVCLKEN

ERLFGDSALGMSVKNPKTVYRHLQSLLGKKHSNLNVALYQKRFPEHQLLEDPVRGT VLFESSEAKWFTPE

ELLGMVLNYSRGLAQDFAEQQIKDAVITVPAFFNQAERRAVLHAAEMAGLKVLQLIN DNTAVALNYGVFR

RKDLDSTVKNVIFYDMGASSTTATVVTYQMVKTKEFGTQPQLQIRGVGFDRGLGGFE MDLRLRDHLAKLF

NEQKKSKKDVRENHRAMAKLLKEAQRLKTVLSANMEFMAQVEGLMDDIDFKSKVT RAEFEELCSDLFERV

PRPLHDALETADLKLDDIELVILVGGSTRVPKVQEVLLKATEKEELGKNINADEAAAM GAVYQAAVLSKA

FKVKPFLIRDAALFPIQVEFTREVEEEGIKTLKQNKRILFQRMAPYPQRKVITFNRYQS DFAFNINYGDM

SFLTPNELSVFGSVNLSTVKLSGVGSSFQKHADAESKGIKAHFNMDESGVLLLDRVES VFETTVDEKEEE

STLTRLGNTISTLFGGGSSDPTVNLTEPVQDDEETPLDSRKDNKDESQKEEAAKDTQS DFEKSSEDEKLQ

EKEEESNDKKDPTEEKDAEKHGKAEPEKKAKLQKKSKISEEITVEQIINDILNPTPDDL ASSKKKLQDLT

NRDLAKQEREKSLNNLEAFIFETQDKLYQEDYSQVVSDQEKEQIAAKLTEVSEWMDE EGYAATTKQLKEK

LSQLKGLCKDMFFRVEERRKWPDRLAALESLLNTSTFFFLGVPNKFQKMTRSSLRLS

>hyou1 Gasterosteus aculeatus

MERRMLAPFLLFSLVLAMLPSRTVTVAVMSIDLGSEWMKVAIVKPGVPMEIVLNKES RRKTPTAVCLKEN

ERFLGDSALGMSVKNPTTVYRHLQGLLGKKYDNLQVALYQKRFPEHQLLEDPVRGT VYFKNSEEMQFTPE

ELLGMLLNYSRGLAQDFAEQPIKDAVITVPAFFNQAERRAVLQAAQMAGVKVLQLIN
DNTAVALNYGVFR

RKDIDNTVKNVMFYDMGSGSTTATIVTYQTVKTKEAGTQPQLQIRGVGFDRGLGGFE
MDLRLRDHLAKLF

NEQKKSKKDVRENQRAMAKLLKEAQRLKTVLSANVDFMAQVEGLMDDIDFKAKV
TRTEFEELCADLFERV

PGPVQEALTTAEMKLEEIEQVILVGGSTRVPKVQEVLLKAVEKEELGKNINADEAAAM
GAVYQAAALSKA

FKVKPFLVRDAAIFPIQVEFTRETEEEGLKIHKHNKRVLFQRMAPYPQRKVITFNRYSA
DFAFYINYGDL

SFLSQHDLSVFGSLNLTTVKLSGVGDSFQKHTDAESKGIKAHFNMDESGVLLLDRVES
VFETIVEDKEEE

STLTKLGNTISTLFGGGSSEPSLNATEPVQDEEEVPPESEKDGKEEEGKAEGEKEEAAK
EKQEEAEKSAD

EETSQEKTEETGSKTDAKEEDGEKSVNAEAEKDAEKDAEKQTKPQKKSKIAEEITVE
LVINDILDPTLDD

VTSSKKKLQDLTDRDLAKQEREKSLNSLEAFIFETQDKLYQEDYQQVVSEEEKEQISS
KLSEASEWMDED

GYAATTKQLREKLSQLKSLCKDMFFRVEERRKWPDRLAALDSMLNTSSFFLRSAKLIP
EDDQIFTQVELN

TLEKVINETAKWKNETVAAQEKNSLTERPVLLSKDIESKLTLLDREVNYLLNKAKFAK
PKAKAKAKNGTS

SGKSSKGNNTEDKVIPPTEESTDTKTENSEEVQPGQAPPTEESTEHTGSDSQSQPTEET
KTTAPASGEAQ

LENHNEL

>hyou1 Larimichthys crocea

MGGRKLALVALFCLVAAMLPSHTVTVAVMSVDLGSEWMKMAIVKPGVPMEIVLNKE
SRRKTPIAVCLKEN

ERLFGDSALGVSVKNPKTVYRHLQSLLGKKHKNPQVALYQKRFPEHQLEEDAVRGT
VYFKYSDEVQYTPE

ELLGMALNYSCGLAQDFAEQPIKDAVITVPAFFNQAERRAVLQAAQMAGLKVLQLIN

DNTAVALNYGVFR

RKDIDSTAKNIMFYDMGAGSTTATIVSYQTVKNKESGTQPQLQIRGVGFDRGLGGFE MDLRLRDHLAKLF

NEQKKSKKDVRENHRAMAKLLKEAQRLKTVLSANMEFMAQVEGLMDDIDFKSKVT RAEFEELCADLFERV

PRPVQEALSVAEMKLDEIEQVILVGGATRVPKVQEVLLKAVGKEELGKNINADEAAA MGAVYQAAALSKA

FKVKPFIVRDAAIFPIQVEFTRETEEDGVKALKHNKRVLFQRMAPYPQRKVITFNRYN ADFSFDINYGDL

SFLSQEDISMFGSLNLTTVKLSGVGSSFQKHGDAESKGIKAHFNMDDSGVLLLDRVES VFETIVEEKEEE

STLTKLGNTISTLFGGGSSEPAPNVTEPVQDEEEVPPESGKDDKDEEGKEEAQKDEAA KEKQDDAEKSAG

EEKNQEKTEEEAGSKAEAKDESNGEKSATEAEKEAEKKAKPQKKSKISEDITAELLVN DILDPTADDVTS

SKKKLQDLTDRDLAKQEREKTLNSLEAFIFETQDKLYQDDYQLVVSEEEKEQMSAKL KEVSEWMDEDGYS

ATTKQLREKLSQLKSLCKDMFFRVEERRKWPERLAALESMLNTSSFFLRSAKLIPEDD QIFTEVELNMLE

KVINETTTWKNETVAEQEKRSPKERPILLSKDIESKLALLDREVNYLLNKAKFAKPKA KAKAKNSTSSEK

NSKANNTAEEKVIPPTEESADAKSETPEEAQPGEAPPTEESTPQSNSDSQSQPSEETTTA GPTEKPPPEN

HIDDEL

>hyou1 Oreochromis niloticus

MMEGRKLVPAALFCLVLATFPSHIVTVAVMSVDLGSEWMKIAIVKPGVPMEIVLNKES RRKTPTVVCLKE

NERLFGDSAMGVSVKNPKSVYRHLQSLLGKKHENLQVALYQKRFPEHHLQEDPVRG TVHFKNSEEMQYTP

EELLGMVLNYSRGLAQDFAEQTIKDAVITVPAFFNQAERRAVLHAAHIAGLKVLQLIN DNTAVALNYGVF

RRKDIDSTAKHVMFYDMGSGSTTATIVTYQTVKTKESGTQPQLQIRGVGFDRGLGGF
EMDLRLRDHLARL

FNEQKKSKKDVRENHRAMAKLLKEAQRLKTVLSANVDFMAQVEGLMDDIDFKAKV
TRSDFEELCADLFER

VPQPVQDALTAAEMKLEDIEQVILVGGSTRVPKVQEVLLKAVGKEELGKNINADEAA
AMGAVYQAAALSK

AFKVKPFLVRDAAVFPIQVEFTREVEEEEGVKTLKHNKRILFQRMATYPQRKVITFNR
YNDDFVFNINYG

DLSFLSQEDLRVFGSLNLTTVKLSGVGSSFQKHADAESKGIKAHFNMDESGVLLLDRV
ESVFETVVEEKE

EESTLTKLGNTISTLFGGGSSEPAPNVTEPVQDEEEVPPESGKDSKDEDGKDENHKDK
ATNEKPNDSEKN

SSEDKNQEKAKEAGSTAEAKEEKDGDKSVNTEVEKKARPQKKSKISEEITLELLINDI
VDPTADDLTSSK

KKLQDLTDRDLAKQEREKTLNSLEAFIFETQDKLYQEDYQQVVTEEEQEQISAKLKE
VSEWMDEDGYAAT

TKQLREKLFQLKSLCKDMFFRVEERRKWPEHLARLESLLNSSSFFLKSARLIPEDDQIF
TDVELNMLEKV

INETMMWKNETTAEQEKRSPKERPVLLSKDIESKRTLLEREVNYLFNKAKFAKPKPK
AKNGTSTDKSSKA

NSTAEENVSPPIEESTDTKSDTESSEEKPSDETPSTESTLDSQSQPKEETATTGKLERTQP
ENHIEDEL

>hspa13 Thamnaconus septentrionalis

MSGEISLIGSVILALFLAGYLGQQYLPPPKARVIGLDLGTTFCSVGIFQAGSGEVEVIAD

EQGRLSIPSTVAFTDTAVLVGHEAADVADSHPENTIYDAKRFIGKIFEPEALEQESARYP

FKVVSNNGSAEFVVNANGSFAVTPEFIGSRLLLKMKKMAERRLGLPIKMAVMSVPAE
FDE

RQRNYTVRAAQLAGLEVLRVINEPTAAAMAYGLHKVDVFNVLVVDLGGGTLDVSLL
NKQG

AMFLTRAMAGNNKLGGQDFSQRLLQFTSERVQQEFGVPPTLKEDIHSLRQAVEAAKI
NLT

LHPSVTIRVPLHLQTQEGSAAAAPAAPAPVLFHTVITRQLFEELNEDLFQKILAPVETVL

AEGNLQKEDVDEIVLVGGSTRIPRIRRLISEFFGKEPNTSVDPDLAVVTGVAIQAGIMGG

SWPLQVSAIEIPNRHLRKTNFS

>hspa4l.1 Thamnaconus septentrionalis

MSVVGIDLGFHNCYIAVARSGGIETITNDYSDRCTPACVSLASKNRIIGNAAKSQIITNF

KNTVHGFKKFHGRAFDDPFVHAEKPKLPYSLHKLANGNTGIKVRYLDEDKVFTIEQITGM

LLNKLKETSEGALKKPVVDCVISVPGYFTDAERRSVFDATQIAGLNCLRLINDTTAVALA

YGIYKQDLPTPEERPRNVVFVDMGHSSFQVSIAAFNKGKLKVLATTFDPYLGGRNFDEVL

ADYFCDEFKSKYKLNVRENPRAVLRLHQECEKLKKLMSANSSDLPLNIECFMNDIDVSSR

MNRGLFEDMCAQYLMRVEAPLKAVLEQSKLSRDDIYAVEIVGGATRMPAIKERIGRFFGK

DISTTLNADEAVARGCALQCAILSPACKVREFSITDVVPFPITLRWKSPTDDGLGECEVF

SKMHAAPFSKVITFHKKEPFDLEALYSNPAELPYPDHRIGCFSVQNVVPQPDGDSSKVKV

KVRINVHGIFSVSGASLIEKQKGEAEDMQIDSEPMVQNEGRAEDQTKMQVDQEGQTQGDQ

LNDDNTSSNKDGAPGEKQDPAAGGSKPKVKVKSTDLPIVTKNIRQLDSSVLNDFVEYERQ

MITQDKLVKELNDAKNAVEEYVYDLRDKLCGIYEKFVNEDDSNRLTLLLEETENWLYEEG

EDQPKQVYEEKLAALKMLGQPIQDRHREHEDRPRAFEELGKKLQLYMKVLEAFKQKDERY

LHLTAEEMSAVEKCVSESMVWMNSRMNAQSKIGITQDPVVKVADVISKIQELENVCNPVI

NRPKPTVEEAPQADDQSGGSNNIPTAKQGADGKADAKGSQQTKAGTKEMEVD

>hspa4a Thamnaconus septentrionalis

MSVVGFDLGFQSCYVGVARAGGIETIANEYSDRCTPSFVSFGQRSRSIGAAAKSQVVT
NS

KNTVQGFKKFHGRPFADPYVQAAKSNLVYDLAQMPSGSTGIKVMYLEEEKVFSVEQ
VTGM

LLTKLKETAESALKKPVADCVISVPSYFTDAERRSVMDAAQIAGLNCLRLMNETTAVT
LA

YGIYKQDLPAPEEKARVVVFVDLGHSGYQVSVCAFNKGKLKILALAFDPELGGKDFD
DIL

VSHFCEDFGKRYKLDVRSKPRALVRLFQECEKLKKLMSANSSDLPLNIECFMNDIDVS
GK

LNRGQFEEMCAGLLAKVEAPLRSVMEQAKLKKEDIYAVEIVGGASRIPSIKERISKFFG
K

ELSTTLNADEAVARGCALQCAILSPAFKVREFSITDVVPYSVSLKWNSAAEDGLSDCE
VF

PKNHPAPFSKVLTFFRKEPFALEAYYNNPKELPYPSSSLGNFLIQNVVPQESGESAKVK
V

KVRVNVHGVFSVASASLIEIVKPAEGEEPMETDQTTKEEENKMQVDQEDQKAPSGDT
EEK

KSEAEEMETTEEKQDKKNEQQPPQAKKPKVKTKTVELPVESKQHWQLTTDVLNMFV
ENEG

KMIMQDKLEKERNDAKNNVEEYVYDMRDKLHGALEKFISDADRDSFSLKLEDVEN
WLYED

GEDQQKQVYIDKLAELKKIGQPVVERYNESVERPKAFEELGRQIQLFMKVVEAFKAK
EEQ

YNHLDELEVTRVDKQLNDAMVWMNGKMNQQNGQDLTLEPVVRVAEIQAKTKELYS
ACNPV

VSKPKPKVEVPKDEKTENGPVNGQEAPESQQQQQPAAANQDKAPSSGPQQEAQGKL
PEMD

ID

>hspa8.1 Thamnaconus septentrionalis

MSKGPAVGIDLGTTYSCVGVFQHGKVEIIANDQGNRTTPSYVAFTDSERLIGDAAKNQ
VA

MNPTNTVFDAKRLIGRRFDDAVVQSDMKHWPFNVINDNTRPKVQVEYKGESKSFYP
EEIS

SMVLTKMKEIAEAYLGKTVNNAVVTVPAYFNDSQRQATKDAGTISGLNVLRIINEPTA
AA

IAYGLDKKVGAERNVLIFDLGGGTFDVSILTIEDGIFEVKSTAGDTHLGGEDFDNRMV
NH

FIAEFKRKFKKDISDNKRAVRRLRTACERAKRTLSSSTQASIEIDSLYEGVDFYTSITRA

RFEELNADLFRGTLDPVEKSLRDAKMDKGQIHDIVLVGGSTRIPKIQKLLQDFFNGKE
LN

KSINPDEAVAYGAAVQAAILSGDKSENVQDLLLLDVTPLSLGIETAGGVMTVLIKRNT
TI

PTKQTQTFTTYSDNQPGVLIQVYEGERAMTKDNNLLGKFELTGIPPAPRGVPQIEVTF
DI

DANGIMNASCSGQEHWKGEQDHHHQRQGSSEQGRH

>hspa14 Thamnaconus septentrionalis

MSAIGIHFGCTSACVAIIKDGRADVVANDAGDRVTPAVVAYRDDEQIVGIAAKQGRTW
NS

ANTVMKVKQLLGRRFDDAETQNYKSQSKCQVVSRQGNPYYEILAGEKPEYVAPVDA
AKAI

LHNMKETAQWALGSDVTDAVFTVPFEFSHSQEQALRDAAEAAGFRVLRLIHEPAAAL
LAY

NIGQESSSGRSHVLVYKLGGASLSISVLQVNGGMFQVLNSHTDLSMGGESFTDALAQ
FLA

TEFERTYKQNVRTNARAMLKLMNGADMAKHSLSSLGSANCFVDSLYNGVDFDCNV
SRARF

ELLCTPIFNKCIQPIRPLLEKVRLTTSDINKVVLCGGSTKIPRLQNMIQDIFPNVDILSS

TPPDEVIAVGAALQAGLLVSRDGPVLEQESTTVEVSATDILVKELDESGAEVFTLLLPS
G

TPLPARRHCVLSGDGKLSSVCMNIYQKLLLEQPQQLAKVILRDLQPKEVNHSIDTVLT
MK

RDGSVHVSCVEQNNGTPEFVTIAATP

>hspa8.2 Thamnaconus septentrionalis

MSKGPAVGIDLGTTYSCVGIFQHGKVEIIANDQGNRTTPSYVAFTDSERLIGDAAKNQ
VA

MNPSNTVFDAKRLIGRKFDDQVVQSDMKYWPFKVVNDSSKPKVEVEYKGEVKSFY
PEEIS

SMVLVKMKEISEAYLGKPVNNAVITVPAYFNDSQRQATKDAGTISGLNVLRIINEPTAA
A

IAYGLDKKVGSERNVLIFDLGGGTFDVSILTIEDGIFEVKSTAGDTHLGGEDFDNRMV
NH

FIAEFKRKFKKEISSNKRAVRRLRTACERAKRTLSSSTQASIEIDSLYEGVDFYTSITRA

RFEELNADLFRGTLDPVEKALRDAKMDKSQIHDIVLVGGSTRIPKIQKLLQDFFNGRD
LN

KSINPDEAVAYGAAVQAAILAGDKSENVQDLLLLDVTPLSLGIETAGGVMTVLIKRNT
TI

PTKQTQTFTTYSDNQPGVLIQVYEGERAMTKDNNILGKFELTGIPPAPRGVPQIEVTFD
I

DANGILNVSAVDKSTGKENKITITNDKGRLSKEDIEKMVQEAEQFKAEDEAQRDKVT
AKN

SLESLAFNMKSTVEDEKLQDKISPEDKKTIVDKCNEVIAWLDRNQMAEKDEYDHKQ
KELE

KVCNPIISKLYQGGGGMPGGMPGGMPGGFPGGAGAGSSSGPTIEEVD

>hspa1b Thamnaconus septentrionalis

MSSAKGISIGIDLGTTYSCVGVFQHGKVEIIANDQGNRTTPSYVAFTDSERLIGDAAKN
Q

VAMNPTNTVFDAKRLIGRKFDDPVVQSDMKLWPFKVVSDSGRPKVQVEHKGETKAF
YPEE

VSSMVLVKMKEIAEAFLGQTVSNAVVTVPAYFNDSQRQATKDAGVIAGLNVLRIINEP
TA

AAIAYGLDKGKRGERNVLIFDLGGGTFDVSILTIDDGIFEVKATAGDTHLGGEDFDNR
MV

RHFVEEFKRKHKKDISQNKRAVRRLRTACERAKRTLSSSTQASIEIDSLFEGIDFYTSIT

RARFEELNSELFRGTLEPVEKALQDAKMDKSKIHEIVLVGGSTRIPKIQKLLQDFFNGR
E

LNKSINPDEAVAYGAAVQAAILMGDTSENVQDLLLLDVAPLSLGIETAGGVMTSLIKR
NT

TIPTKQTQIFSTYSDNQPGVLIQVYEGERAMTKDNNLLGKFELVGIPPAPRGVPQIEVT
F

DIDANGILNVSAVDKSTGKENKITITNDKGRLSKEEIERMVQDSEKYKAEDDSQREKI
AA

KNSLETYAYHMKSCVEDEKLAGKISDADKRTVVDKCNQAISWLDNNQLAEKDEYEH
QQKE

LEKVCNPVVTKLYQGAAPSGGGAAQSGGSSKGPTIEEVD

>hspa2 Thamnaconus septentrionalis

MSGKKGVAIGIDLGTTYSCVGVFQHGKVEIIANDQGNRTTPSYVAFTDTERLIGDAAK
NQ

VALNPTNTVFDAKRLIGRKLDEPVVQADMKLWPFKVVGHEGKPKIQVEYKGEQKQF
HPEE

ISSMVLVKMKEIAEAYLGQKVSQAVVTVPAYFNDSQRQATKDAGVIAGLNVMRIINEP
TA

AAIAYGMDKGKSGERKVLIFDLGGGTFDVSVLAIEDGIFEVKSTAGDTHLGGEDFDN
RMV

KHFVDEFKRKHKKDISGNKRSLRRLRTACERAKRTLSSSTQASIEIDSLFEGIDFYTSIT

RARFEELCSDLFRGTLEPVEQALKDAKLDKASIEEIVLVGGSTRIPKIQKLLQDFFNNK
E

LNKSINPDEAVAYGAAVQAAILSGDTSSNVSDLLLLDVAPLSLGIETAGGVMTPLIKRN
S

TIPTKQSQTFTTYSDNQPGVLIQVFEGERAMTKDNNLLGKFELAGIPPAPRGVPQIDVT
F

DVDANGILNVSAVDKSTGKENKITITNDKGRLSKEEIERMVNDAEKYKDEDIAQKEKI

GA

KNSLESYIFQVRSSVQDEKLKDKISEEERKTVVDKCDEVIAWLENNQLADKEEFHDK
QKE

VEKVFSPIISKLYQGGMPAGDPGRQSQCGSQGPTIEEVD

>hspa4l.2 Thamnaconus septentrionalis

MSVVGFDVGFLNCYVAVARAGGIETVANEYSDRSTPACVSFGPRNRSIGAAAKSQLVT
NC

KNTVQGFKRFHGRAFSDPYVQRLKNSTIYDVVQMPTGTAGIKVMYMEEEKAFSIEQ
VTAM

LLTKLKETVQNTLKKPVSYCVVSVPCYYSDAERRSVLDAAQIAGLNCLRLMNETTAV
ALA

YGIYKQDLPAPEEKARNVVFVDLGHSGYQTSVCAFNKGKLKILSTACDPELGGKDFD
EVL

VKHFCEEFAKKYKLDVKSKPRALVRLYQECEKLKKLMSANSSDLPLNIECFMNDIDV
SAK

LNRGHFEEMCVDILARVEPPLHNLLENAKLKKEDIHAVEIVGGASRIPAVKERISKFFG
K

ELNTTLNADEAVARGCALQCAILSPAFKVREFSITDAVPYPISLKWQSAAEEGLSDCEV
F

PRNHAAPFSKVLTFYRKEPFSLEAYYNSPDELPYPDPTIGKYLIQKVVPQASGESSKVK
V

KVRVNIHGIFSVSSASLVEVQKSDETEEPMETEQANDKEEQPKMQTDQDEQQSQGDG
QNE

TEESTPEENEEMQNTTEEPKGEKKSDQPPQAKKPKVKTKLLDLPIENSPQWQLADDM
LNL

FVENEGKMIMQDKLEKERNDAKNNVEEYVYDMRDKLHGLLEKYVSESDRDAFSSK
LEDTE

NWLYEDGEDQPKQVYIDKLSDLKKLGQPIQERYTEAELRPKAFEEMGKQIQQYMKF
VEAY

KMKEEQYEHIDEADVTKVDKLACEAMMWMNSSMNQQNKLNLSVDPAIKIKDIQAK
TKELF

SACNPIVTKPKPKVELPKEDAAAEQNGPVNGQEQGRKEGTGEGATESADADPASETT
ENK

PDMDQD

>hspa5 Thamnaconus septentrionalis

MKLLWVVLLFAGTVLADADDDEKKESVGTVVGIDLGTTYSCVGVFKNGRVEIIAND
QGNR

ITPSYVAFTSEGERLIGDAAKNQLTSNPENTVFDAKRLIGRTWGDTSVQQDIKYFPFKV
T

EKKSKPHIQVDIGGGQMKTFAPEEISAMVLTKMKETAEAYLGKKVTHAVVTVPAYFN
DAQ

RQATKDAGTIAGLNVMRIINEPTAAAIAYGLDKRDGEKNILVFDLGGGTFDVSLLTIDN
G

VFEVVATNGDTHLGGEDFDQRVMEHFIKLYKKKTGKDVRKDNRAVQKLRREVEKAK
RALS

AQHQARIEIESFFEGEDFSETLTRAKFEELNMDLFRSTMKPVQKVLEDSDLKKNDIDEI
V

LVGGSTRIPKIQQLVKEFFNGKEPSRGINPDEAVAYGAAVQAGVLSGEEDTGDVVLLD
VC

PLTLGIETVGGVMTKLIGRNTVVPTKKSQIFSTASDSQPTVTIKVYEGERPLTKDNHLL
G

TFDLTGIPPAPRGVPQIEVTFEIDVNGILRVTAEDKGTGNKNKITITNDQNRLTPEDIER

MVNDAERFADEDKKLKERIDSRNELESYAYSLKNQIGDKEKLGGKLSDDDKETIEKA
VEE

KIEWMESHQDAELEDFQAKKKELEEIVQPIISKLYGSAGGPPPEGAEGTQEDKDEL

>hspa9 Thamnaconus septentrionalis

MLSVARSVSRTLPTSAGCTRSVSSLVKKACWSGFQQDALRTLSRRDYASEAVKGAVIG
ID

LGTTNSCVAVMEGKQAKVLENAEGARTTPSVIAFTADGERLVGMPAKRQAVTNPQNT
LYA

TKRLIGRRYDDAEVQKDLKNVPFKIVRASNGDAWVEAHGKMYSPSQSGAFVLMKM
KETAE

NYLGTKVKNAVVTVPAYFNDSQRQATKDAGQIAGLNVLRVINEPTAAALAYGLDKTQDKI

IAVYDLGGGTFDISVLEIQKGVFEVKSTNGDTFLGGEDFDQHLLRHIVKEFKRESGVDLT

KDNMALQRVREAAEKAKCELSSSLQTDINLPYLTMDASGPKHLNMKLTRSQFEGIVADLI

RRTVAPCQKAMQDAEVSKGDIGEVLLVGGMSRMPKVQQTVQDLFGRAPSKSVNPDEAVAI

GAAIQGGVLAGDVTDVLLLDVTPLSLGIETLGGVFTKLINRNTTIPTKKSQVFSTAADGQ

TQVEIKVCQGEREMASDNKVLGQFTLVGVPPAPRGVPQIEVTFDIDANGIVHVSAKDKGT

GREQQIVIQSSGGLSKDDIENMVKNAEKYAEEDRRRKDRVEAVNMAEGIVHDTESKMEEF

KDQLPADECAKLKEEIAKVREVLANKDSETGENIKQAANTLQQSSLKLFEMAYKKMAADR

EGSSGSSGSSSGSSSSSSSEGEKKEGQQ

>hyou1 Thamnaconus septentrionalis

MEARKLTLVAVSCLVLAMLPSQTDTVAVMSVDLGSEWMKMAIVKPGVPMEIVLNKESRRK

TPTAVCLKENERLLGDSALGMSVKNPKTVYRHLQILLGKKHDNPQVALYQKRFPEHQLQE

DPVRGTVYFKFSEDIQYTPEELLGMMLNYSCGLAQDFAEQPIKDAVITVPAFFNQAERRA

VLQAAQMAGLKVLQLINDNTAVALNYGVFRRKDIDGTPKSVMFYDMGSGSTTATIVTYQT

VKSKEFGTQPQLQIRGVGFDRGLGGFEMDIRLRDHLAKLFNGQKKSKKDVTGNHRAMAKL

LKEAQRLKTVLSANVDFMAQVEGLMDDIDFKAKVTRAEFEELCADLFERVPRPVQDALAA

AEMSMVEIEHVILVGGSTRVPKVQEVLLKAVGKEELSKNINADEAAAMGAVYQAAA

LSKA

FKVKTFLIRDAAVFPILVEFDRETEEDGGKTLKHSKRILFQRMAPYPQRKVITFNRYTN
D

FAFDINYGDLNFLSQEDISVFGSLNLTTVKLSGVGSSFLKHTDAESKGIKAHFNMGRS
CC

FHSQVESVFETVEEEKEEESTLTKLGNTISTLFGGGSSEPAPNVTEPVQDEEEVPPESGK

EGKEEEGKDEAPKEEAPKDTQEDAEKSAGEEKNQEKTEEAGSNTDAKETEAEKSGN
AEAE

KDVEKAKPKKKSKISEDITVELVLKDMRDPTAAALTSSKKKLQDLTDRDLAKHEREK
MLN

SLEAFIFETQDKLYQDEYQLVVSEEETAQITGKLREASEWMDEDGYSATTKELREKLS
QL

RSLCKDMFFRRLAALESMLNTSSFFLRSARLIPEDDQIFTEVELNMLDKVINETTVRR
WW

QKQDKRSVKERPVLLSKDIESKLSLLDPRVNYLLNKAKFAKPRAKPRPKTAVIPPSKEE
R

RTKRIVGERRMTRNPSHRSAPGRLFFSLLNIYHTSNDKSENIFYL

HSP90 tree_all species protein sequences

>HSP90AA1 Homo sapiens

MPEETQTQDQPMEEEEVETFAFQAEIAQLMSLIINTFYSNKEIFLRELISNSSDALDKIR

YESLTDPSKLDSGKELHINLIPNKQDRTLTIVDTGIGMTKADLINNLGTIAKSGTKAFM
E

ALQAGADISMIGQFGVGFYSAYLVAEKVTVITKHNDDEQYAWESSAGGSFTVRTDTG
EPM

GRGTKVILHLKEDQTEYLEERRIKEIVKKHSQFIGYPITLFVEKERDKEVSDDEAEEKE
D

KEEEKEKEEKESEDKPEIEDVGSDEEEEKKDGDKKKKKKIKEKYIDQEELNKTKPIWT
RN

PDDITNEEYGEFYKSLTNDWEDHLAVKHFSVEGQLEFRALLFVPRRAPFDLFENRKK

KNN

IKLYVRRVFIMDNCEELIPEYLNFIRGVVDSEDLPLNISREMLQQSKILKVIRKNLVKKC

LELFTELAEDKENYKKFYEQFSKNIKLGIHEDSQNRKKLSELLRYYTSASGDEMVSLK
DY

CTRMKENQKHIYYITGETKDQVANSAFVERLRKHGLEVIYMIEPIDEYCVQQLKEFEG
KT

LVSVTKEGLELPEDEEEKKKQEEKKTKFENLCKIMKDILEKKVEKVVVSNRLVTSPCC
IV

TSTYGWTANMERIMKAQALRDNSTMGYMAAKKHLEINPDHSIIETLRQKAEADKND
KSVK

DLVILLYETALLSSGFSLEDPQTHANRIYRMIKLGLGIDEDDPTADDTSAAVTEEMPPL
E

GDDDTSRMEEVD

>hsp90aa1 Mus musculus

MPEETQTQDQPMEEEEVETFAFQAEIAQLMSLIINTFYSNKEIFLRELISNSSDALDKIR

YESLTDPSKLDSGKELHINLIPSKQDRTLTIVDTGIGMTKADLINNLGTIAKSGTKAFM
E

ALQAGADISMIGQFGVGFYSAYLVAEKVTVITKHNDDEQYAWESSAGGSFTVRTDTG
EPM

GRGTKVILHLKEDQTEYLEERRIKEIVKKHSQFIGYPITLFVEKERDKEVSDDEAEEKE
E

KEEEKEKEEKESDDKPEIEDVGSDEEEEEKKDGDKKKKKKIKEKYIDQEELNKTKPI
WTR

NPDDITNEEYGEFYKSLTNDWEEHLAVKHFSVEGQLEFRALLFVPRRAPFDLFENRKK
KN

NIKLYVRRVFIMDNCEELIPEYLNFIRGVVDSEDLPLNISREMLQQSKILKVIRKNLVKK

CLELFTELAEDKENYKKFYEQFSKNIKLGIHEDSQNRKKLSELLRYYTSASGDEMVSL
KD

YCTRMKENQKHIYFITGETKDQVANSAFVERLRKHGLEVIYMIEPIDEYCVQQLKEFE
GK

TLVSVTKEGLELPEDEEEKKKQEEKKTKFENLCKIMKDILEKKVEKVVVSNRLVTSPCCI

VTSTYGWTANMERIMKAQALRDNSTMGYMAAKKHLEINPDHSIIETLRQKAEADKNDKSV

KDLVILLYETALLSSGFSLEDPQTHANRIYRMIKLGLGIDEDDPTVDDTSAAVTEEMPPL

EGDDDTSRMEEVD

>hsp90aa1 Gallus gallus

MPEAVQTQDQPMEEEVETFAFQAEIAQLMSLIINTFYSNKEIFLRELISNSSDALDKIRYESLTDPSKLD

SGKDLKINLIPNKHDRTLTIVDTGIGMTKADLVNNLGTIAKSGTKAFMEALQAGADISMIGQFGVGFYSA

YLVAEKVTVITKHNDDEQYAWESSAGGSFTVRLDNGEPLGRGTKVILHLKEDQTEYLEERRIKEIVKKHS

QFIGYPIRLFVEKERDKEVSDDEAEEKEEEKEEKEEKTEDKPEIEDVGSDEEEEKKDGDKKKKKKIKEKY

IDEEELNKTKPIWTRNPDDITNEEYGEFYKSLTNDWEDHLAVKHFSVEGQLEFRALLFVPRRAPFDLFEN

RKKKNNIKLYVRRVFIMDNCEELIPEYLNFMRGVVDSEDLPLNISREMLQQSKILKVIRKNLVKKCLELF

TELAEDKENYKKFYEQFSKNIKLGIHEDSQNRKKLSELLRYYTSASGDEMVSLKDYCTRMKENQKHVYYI

TGETKDQVANSAFVERLRKHGLEVIYMIEPIDEYCVQQLKEFEGKTLVSVTKEGLELPEDEEEKKKQEEK

KAKFENLCKIMKDILEKKVEKVVVSNRLVTSPCCIVTSTYGWTANMERIMKAQALRDNSTMGYMAAKKHL

EINPDHSIIETLRQKAEADKNDKSVKDLVILLYETALLSSGFSLEDPQTHANRIYRMIKLGLGIDEDDTA

AEEASPAVTEEMPPLEGDDDTSRMEEVD

>hsp90aa1.1 Danio rerio

MPEKSAQPVMEEEVETFAFQAEIAQLMSLIINTFYSNKEIFLRELISNSSDALDKIRYES

LTDPSKLDSCKDLKIELIPDQKERTLTIIDTGIGMTKADLINNLGTIAKSGTKAFMEALQ

AGADISMIGQFGVGFYSAYLVAEKVTVITKHNDDEQYIWESAAGGSFTVKPDFGESIG
RG

TKVILHLKEDQSEYVEEKRIKEVVKKHSQFIGYPITLYIEKQREKEVDLEEGEKQEEEE
V

AAGEDKDKPKIEDLGADEDEDSKDGKNKRKKKVKEKYIDAQELNKTKPIWTRNPDD
ITNE

EYGEFYKSLSNDWEDHLAVKHFSVEGQLEFRALLFVPRRAAFDLFENKKKRNNIKLY
VRR

VFIMDNCEELIPEYLNFIKGVVDSEDLPLNISREMLQQSKILKVIRKNLVKKCLDLFTE
L

AEDKDNYKKYYEQFSKNIKLGIHEDSQNRKKLSDLLRYYTSASGDEMVSLKDYVSR
MKDT

QKHIYYITGETKDQVANSAFVERLRKAGLEVIYMIEPIDEYCVQQLKEYDGKNLVSVT
KE

GLELPEDEEEKKKQDELKAKYENLCKIMKDILDKKIEKVTVSNRLVSSPCCIVTSTYG
WT

ANMERIMKSQALRDNSTMGYMTAKKHLEINPAHPIVETLREKAEADKNDKAVKDLVI
LLF

ETALLSSGFTLDDPQTHANRIYRMIKLGLGIDDDDSVVEEISQPAEEDMPVLEGDDDT
SR

MEEVD

>hsp90aa1.2 Danio rerio

MPEAHEQQMMEDEEVETFAFQAEIAQLMSLIINTFYSNKEIFLRELISNSSDALDKIRY
E

SLTDPSKLDSGKDLKIEIIPNKEERTLTIIDTGIGMTKADLINNLGTIAKSGTKAFMEAL

QAGADISMIGQFGVGFYSAYLVAEKVTVITKHLDDEQYAWESSAGGSFTVKVDNSEPI
GR

GTKVILHLKEDQTEYIEERRIKEIVKKHSQFIGYPITLFVEKERDKEVSDDEAEEEKEK
E

KEEEEEGEKDEDKPEIEDVGSDEDDHDHGDKCGDKKKKKKKIKEKYIDQEELNKTK

PLWT

RNPDDITNEEYGEFYKSLTNDWEDHLAVKHFSVEGQLEFRALLFVPRRAPFDLFENK
KKK

NNIKLYVRRVFIMDNCDELIPEYLNFIKGVVDSEDLPLNISREMLQQSKILKVIRKNLV
K

KCLELFTELAEDKDNYKKYYEQFSKNIKLGIHEDSQNRKKLSELLRYYTSASGDEMV
SLK

DYVTRMKDTQKHIYYITGETKDQVANSAFVERLRKAGLEVIYMIEPIDEYCVQQLKE
FEG

KNLVSVTKEGLELPEDEEEKKKQEEKKSKFENLCKIMKDILEKKVEKVTVSNRLVSSP
CC

IVTSTYGWTANMERIMKAQALRDNSTMGYMAAKKHLEINPDHPIVETLRQKAEADK
NDKS

VKDLVILLFETALLSSGFTLDDPQTHSNRIYRMIKLGLGIDEDDLSAEEPSSAPIEEMPP

LEGDDDTSRMEEVD

>hsp90aa1.1 Oryzias latipes

MPENAAHVMEEEVETFAFQAEIAQLMSLIINTFYSNKEIFLRELISNSSDALDKIRYESL

TDPSKLDSCKELKIEVRPDLHARTLTINDTGIGMTKADLINNLGTIAKSGTKAFMEAL
QA

GADISMIGQFGVGFYSAYLVAEKVTVITKHNDDEQYIWESAAGGSFTVKPDNGEPIGR
GT

KVILHLKEDQTEYCEEKRIKEVIKKHSQFIGYPITLYVEKTREKEVDLEEGEKVEEVEK
E

SAENKDKPQIEDVGSDEDEDTKDGKNKRKKKVKEKYIDAQELNKTKPIWTRNPDDIT
NEE

YGEFYKSLTNDWEDHLAVKHFSVEGQLEFRALLFVPRRAAFDLFENKKKRNNIKLYV
RRV

FIMDNCDELIPEYLNFIKGVVDSEDLPLNISREMLQQSKILKVIRKNLVKKCLELFTELS

EDKDNYKKLYEQFSKNIKLGIHEDSQNRKKLSELLRYYTSASGDEMVSLKDYVSRMK
DNQ

KHIYYITGETKDQVANSAFVERLRKAGLEVIYMIEPIDEYCVQQLKDFDGKNLVSVTK
EG

LELPEDEEEKKKQEEKKAQFENLCKIMKDILEKKVEKVTVSNRLVSSPCCIVTSTYGW
TA

NMERIMKAQALRDNSTMGYMAAKKHLEINPDHPIMETLRQKAEADKNDKSVKDLVI
LLFE

TALLSSGFTLEDPQTHANRIYRMIKLGLGIDEDDTPVEETTSAPTEDMPPLEGDDDASR
M

EEVD

>hsp90aa1.2 Oryzias latipes

MPENAAHVMEEEVETFAFQAEIAQLMSLIINTFYSNKEIFLRELISNSSDALDKIRYESL

TDPAKLDSGKELKIEITPNKQERTLTLMDTGIGMTKADLINNLGTIAKSGTKAFMEAL
QA

GADISMIGQFGVGFYSAYLVAEKVTVITKHNDDEQYAWESSAGGSFTVKLDNSEPLG
RGT

KVILHLKEDQSEYLEERRIKEIVKKHSQFIGYPITLFVEKERDKEVSDDEAEEEEKEKD
K

EKNEEEKDEDKPEIEDVGSDEEDDHDKSSDKKKKKKKIKEKYIDQEELNKTKPLWTR
NPD

DITNEEYGEFYKSLTNDWEDHLAVKHFSVEGQLEFRALLFVPRRAPFDLFENKKKKN
NIK

LYVRRVFIMDNCDELIPEYLNFIRGVVDSEDLPLNISREMLQQSKILKVIRKNLVKKCL
E

LFTELSEDKDNYKKLYEQFSKNIKLGIHEDSQNRKKLSELLRYYTSASGDEMVSLKDY
VT

RMKDNQKHIYYITGETKDQVANSAFVERLRKAGLEVIYMIEPIDEYCVQQLKDFDGK
NLV

SVTKEGLELPEDEEEKKKQEEKKAQFENLCKIMKDILEKKVEKVTVSNRLVSSPCCIV
TS

TYGWTANMERIMKAQALRDNSTMGYMAAKKHLEINPDHPIMETLRQKAEADKNDK
SVKDL

VILLFETALLSSGFTLEDPQTHANRIYRMIKLGLGIDEDDTPVEETTSAPTEDMPPLEG
D

DDASRMEEVD

>hsp90aa1 Larimichthys crocea

MPEKAGNVMEEDVETFAFQAEIAQLMSLIINTFYSNKEIFLRELISNSSDALDKIRYESL
TDPSRLESCK

ELKIEVRPDLLNRTLTLIDTGIGMTKADLINNLGTIAKSGTKAFMEALQAGADISMIGQ
FGVGFYSAYLV

ADKVTVITKHNDDEQYVWESAAGGSFTVKPDPGEPIGRGTKVILHLKEDQTEYCEEK
RIKEVVKKHSQFI

GYPITLYVEKTREKEVDLEDGAKVEEIEKEAAEAAENKDKPKIEDVGSDEDEDTKDG
QNKRKKKVKEKYI

DAQELNKTKPIWTRNPDDITNEEYGEFYKSLTNDWEDHLAVKHFSVEGQLEFRALLF
VPRRAAFDLFENK

KKKNNIKLYVRRVFIMDNCEELIPEYLNFIKGVVDSEDLPLNISREMLQQSKILKVIRK
NLVKKCLELFT

ELAEDKDNYKKCYEQFSKNVKLGIHEDSQNRKKLSELLRYYTSASGDEMVSLKDYC
SRMKENQKHIYYIT

GETKDQVANSAFVERLRKAGLEVIYMIEPIDEYCVQQLKEYDGKNLVSVTKEGLELP
EDEDEKKKQEELK

NKFENLCKIMKDILDKKIEKVTVSNRLVASPCCIVTSTYGWTANMERIMKSQALRDNS
TMGYMTAKKHLE

INPMHPIIETLREKAEADKNDKAVKDLVILLFETALLSSGFTLEDPQTHANRIYRMIKL
GLGIDDDDSAV

EDLIQPADEDMPVLEGDDDTSRMEEVD

>hsp90aa1.1 Oreochromis niloticus

MESISAHIMEEEVETFAFQAEIAQLMSLIINTFYSNKEIFLRELISNSSDALDKIRYESL

TDPTKLESCKDLKIEIRPDLHARTLTILDTGIGMTKADLINNLGTIAKSGTKAFMEALQ
A

GADISMIGQFGVGFYSAYLVAEKVTVITKHNDDEQYMWESAAGGSFTVRPDTGEPIG
RGT

KVILHLKEDQTEYCEEKRVKEVVKKHSQFIGYPITLFKTREKEVDLEEGEKEEEVEKE
AA

EDKDKPKIEDVGSDEDEDTKDGKNKRKKKVKEKYIDAQELNKTKPIWTRNPDDITNE
EYG

EFYKSLTNDWEDHLAVKHFSVEGQLEFRALLFVPRRAAFDLFENKRKRNNIKLYVRR
VFI

MDNCEELIPEYLNFIKGVVDSEDLPLNISREMLQQSKILKVIRKNLVKKCLELFSELAE
D

KDNYKKFYEQFSKNIKLGIHEDSQNRKKLSELLRYYTSASGDEMVSLKDYVSRMKD
NQKH

IYYITGETKDQVANSAFVERLRKAGLEVIYMIEPIDEYCVQQLKEYDGKNLVSVTKEG
LE

LPEDEEEKKKQEELKTKFEELCKIMKDILDKKIEKVVVSNRLVASPCCIVTSTYGWTA
NM

ERIMKSQALRDNSTLGYMTAKKHLEINPLHPIIETLREKAEADKNDKAVKDLVILLFET
A

LLSSGFTLEDPQTHANRIYRMIKLGLGIDDDDSAVEDIIQPADEDMPVLEGDDDTSRM
EE

VD

>hsp90aa1.2 Oreochromis niloticus

MLIPQCYIEMPEMHDQPMEEEAETFAFQAEIAQLMSLIINTFYSNKEIFLRELISNSSDA

LDKIRYESLTDPSKLETGKDLKIEIIPNKEERTLTLVDTGIGMTKADLINNLGTIAKSGT

KAFMEALQAGADISMIGQFGVGFYSAYLVAEKVTVVTKHNDDEQYAWESSAGGSFT
VKVD

NSEPLGRGTKVILHLKEDQTEYLEERRVKEIVKKHSQFIGYPITLFVEKERDKEVSDDE
A

EEEKEKEEKEKDEEKDEDKPEIEDVGSDEEQDHEKSDKKKKKKKIKEKYIDQEELNK
TKP

LWTRNPDDITNEEYGEFYKSLTNDWEDHLAVKHFSVEGQLEFRALLFVPRRAPFDLFE
NK

KKKNNIKLYVRRVFIMDNCEELIPEYLNFIKGVVDSEDLPLNISREMLQQSKILKVIRK

N

LVKKCLELFTELAEDKDNYKKFYEQFSKNIKLGIHEDSQNRKKLSELLRYYTSASGDE
MV

SLKDYVTRMKDNQKHIYYITGETKDQVANSAFVERLRKAGLEVIYMIEPIDEYCVQQ
LKE

FEGKNLVSVTKEGLELPEDEEEKKKQEEKKSQFENLCKIMKDILEKKVEKVTVSNRL
VSS

PCCIVTSTYGWTANMERIMKAQALRDNSTMGYMAAKKHLEINPDHPIMETLRQKAE
ADKN

DKSVKDLVILLFETALLSSGFTLDDPQTHSNRIYRMIKLGLGIDEDDVTSDDNTSAPTE
D

MPPLEGEDDDTSRMEEVD

>hsp90aa1 Ictalurus punctatus

MPEAHDQPMEEEAETFAFQAEIAQLMSLIINTFYSNKEIFLRELISNSSDALDKIRYESL
TDPSKLDSGK

DLKIEIIPNKEDRTLTIIDTGIGMTKADLINNLGTIAKSGTKAFMEALQAGADISMIGQF
GVGFYSAYLV

AENVTVITKHNDDEQYAWESSAGGSFTVKVDNSEPIKRGTKVILHLKEDQTEYIEEKR
IKEIVKKHSQFI

GYPITLYVEKERDKEVSDDEAEEEKEKDKSEEEGEKDDDKPEIEDVGSDDEDHDKNT
DKKKKKKIKEKYI

DQEELNKTKPLWTRNPDDITNEEYGEFYKSLTNDWEDHLAVKHFSVEGQLEFRALLF
VPRRAPFDLFENK

KKKNNIKLYVRRVFIMDNCDELIPEYLNFIKGVVDSEDLPLNISREMLQQSKILKVIRK
NLVKKCLELFT

ELAEDKDNYKKYYEQFSKNIKLGIHEDSQNRKKLSELLRYYTSASGDEMVSLKDYVT
RMKDTQKQIYYIT

GETKEQVANSAFVERLRKAGLEVIYMIEPIDEYCVQQLKEFEGKNLVSVTKEGLELPE
DEEEKKKQEEKK

AQFENLCKIMKDILEKKVEKVTVSNRLVASPCCIVTSTYGWTANMERIMKAQALRDN
STMGYMAAKKHLE

INPDHPIIETLRQKAEADKNDKSVKDLVILLFETALLSSGFTLDDPQTHSNRIYRMIKLG
LGIEEDDLST

EEPSSAPIEDMPPLEGDDDTSRMEEVD

>hsp90aa1 Lepisosteus oculatus

MPEEVKNSVPSQPMDEEVETFAFQAEIAQLMSLIINTFYSNKEIFLRELISNSSDALDKI
RYESLTDPSR

LESCKQLKIDIIPDQLTRTLTIIDTGIGMTKADLINNLGTIAKSGTKAFMEALQAGADIS
MIGQFGVGFY

SAYLVAEKVTVITKHNDDEQYVWESAAGGSFTVKPDSSEPLGRGTKVILHLKEDQVE
YVEEKRIKEIVKK

HSQFIGYPITLYVEKQREKEVDMDEDEKEEETEKKDEAEGAKGDKPEIEDVGSDEEE
DKKEEKKKRKKKI

KEKYIDSQELNKTKPIWTRNPDDITNEEYGEFYKSLTNDWEDHLAVKHFSVEGQLEFR
ALLFVPRRAAFD

LFENRKKRNNIKLYVRRVFIMDNCEELMPEYLNFIKGVVDSEDLPLNISREMLQQSKI
LKVIRKNLVKKC

LELFTELAEDKDNYKKYYEQFSKNIKLGIHEDSQNRKKLSELLRYYTSASGDEMVSL
KDYVTRMKDNQKH

IYYITGESKEQVANSAFVERLRKAGLEVIYMIEPIDEYCVQQLKEFEGKNLVSVTKEGL
ELPEDEEEKKR

QEEKKAQFENLCKIMKDILEKKVEKVAVSNRLVSSPCCIVTSTYGWTANMERIMKSQ
ALRDNSTMGYMAA

KKHLEINPEHPIIETLRQKAEVDKNDKSVKDLVILLFETALLSSGFSLDDPQTHANRIY
RMIKLGLGIDE

DDMTTEDTTAAPIEDMPPLEGDDDTSRMEEVD

>hsp90aa1 Thamnaconus septentrionalis

MPEKAGHVVEEDMETFAFQAEIAQLMSLIINTFYSNKEIFLREVISNSSDALDKIRYES
L

TDPSRLESCKDLKIELRPDLHARTLTIIDTGIGMTKADLINNLGTIAKSGTKAFMEALQ
A

GADISMIGQFGVGFYSAYLVADKVTVITKHNDDEQYIWESAAGGSFTVKPDTGESIGR

GT

KVILHLKEDQTEYCEEKRVKDVVKKHSQFIGYPITLFVEKTREKEVDLQEGEKEEEVE
KE

AAENTDKPKIEDVGSDEDEDSKDGQNKRKKKVKEKYMDAQELNKTKPIWTRNPDDI
TNEE

YGEFYKSLTNDWEDHLAVKHFSVEGQLEFRTLLFVPQRAPFDLFENKKKKNNIKLYV
RRV

FIMDNCEELIPEYLNFIKGVVDSEDLPLNISREMLQQSKILKVIRKNLVKKCLELFAELA

EDKENYKKWYEQFSKNIKLGIHEDSQNRKKLSELLRYWTSASGDEMVSLKDYVTRM
KDNQ

KHIYYITGETKDQVANSAFVERLRKAGLEVIYMIEPIDEYCVQQLKEFEGKNLVSVTK
EG

LELPEDEDEKKKLEDLKTKFESLCKIMKDILEKKIEKVTVSIRLVSSPCCIVTSTYGWT
A

NMERIMKAQALRDTSTMGYMAAKKHLEINPKHPIVETLRVKAEADKNDKSVKDLVN
LLFE

TALLSSGFSLDDPQTHSNRIYRMIKLGLGIDEDDPTSDEPTVAPTEDMPPLEGDDDDTS
R

MEEVD

>HSP90AB1 Homo sapiens

MPEEVHHGEEEVETFAFQAEIAQLMSLIINTFYSNKEIFLRELISNASDALDKIRYESLT

DPSKLDSGKELKIDIIPNPQERTLTLVDTGIGMTKADLINNLGTIAKSGTKAFMEALQA
G

ADISMIGQFGVGFYSAYLVAEKVVVITKHNDDEQYAWESSAGGSFTVRADHGEPIGR
GTK

VILHLKEDQTEYLEERRVKEVVKKHSQFIGYPITLYLEKEREKEISDDEAEEEKGEKEE
E

DKDDEEKPKIEDVGSDEEDDSGKDKKKKTKKIKEKYIDQEELNKTKPIWTRNPDDIT
QEE

YGEFYKSLTNDWEDHLAVKHFSVEGQLEFRALLFIPRRAPFDLFENKKKKNNIKLYVR
RV

FIMDSCDELIPEYLNFIRGVVDSEDLPLNISREMLQQSKILKVIRKNIVKKCLELFSELA

EDKENYKKFYEAFSKNLKLGIHEDSTNRRRLSELLRYHTSQSGDEMTSLSEYVSRMK
ETQ

KSIYYITGESKEQVANSAFVERVRKRGFEVVYMTEPIDEYCVQQLKEFDGKSLVSVTK
EG

LELPEDEEEKKKMEESKAKFENLCKLMKEILDKKVEKVTISNRLVSSPCCIVTSTYGW
TA

NMERIMKAQALRDNSTMGYMMAKKHLEINPDHPIVETLRQKAEADKNDKAVKDLV
VLLFE

TALLSSGFSLEDPQTHSNRIYRMIKLGLGIDEDEVAAEEPNAAVPDEIPPLEGDEDASR
M

EEVD

>HSP90B1 Homo sapiens

MRALWVLGLCCVLLTFGSVRADDEVDVDGTVEEDLGKSREGSRTDDEVVQREEEAI
QLDG

LNASQIRELREKSEKFAFQAEVNRMMKLIINSLYKNKEIFLRELISNASDALDKIRLISL

TDENALSGNEELTVKIKCDKEKNLLHVTDTGVGMTREELVKNLGTIAKSGTSEFLNK
MTE

AQEDGQSTSELIGQFGVGFYSAFLVADKVIVTSKHNNDTQHIWESDSNEFSVIADPRG
NT

LGRGTTITLVLKEEASDYLELDTIKNLVKKYSQFINFPIYVWSSKTETVEEPMEEEEAA
K

EEKEESDDEAAVEEEEEEKKPKTKKVEKTVWDWELMNDIKPIWQRPSKEVEEDEYK
AFYK

SFSKESDDPMAYIHFTAEGEVTFKSILFVPTSAPRGLFDEYGSKKSDYIKLYVRRVFITD

DFHDMMPKYLNFVKGVVDSDDLPLNVSRETLQQHKLLKVIRKKLVRKTLDMIKKIA
DDKY

NDTFWKEFGTNIKLGVIEDHSNRTRLAKLLRFQSSHHPTDITSLDQYVERMKEKQDKI
YF

MAGSSRKEAESSPFVERLLKKGYEVIYLTEPVDEYCIQALPEFDGKRFQNVAKEGVKF
DE

SEKTKESREAVEKEFEPLLNWMKDKALKDKIEKAVVSQRLTESPCALVASQYGWSGNMER

IMKAQAYQTGKDISTNYYASQKKTFEINPRHPLIRDMLRRIKEDEDDKTVLDLAVVLFET

ATLRSGYLLPDTKAYGDRIERMLRLSLNIDPDAKVEEEPEEEPEETAEDTTEDTEQDEDE

EMDVGTDEEEETAKESTAEKDEL

>TRAP1 Homo sapiens

MARELRALLLWGRRLRPLLRAPALAAVPGGKPILCPRRTTAQLGPRRNPAWSLQAGRLFS

TQTAEDKEEPLHSIISSTESVQGSTSKHEFQAETKKLLDIVARSLYSEKEVFIRELISNA

SDALEKLRHKLVSDGQALPEMEIHLQTNAEKGTITIQDTGIGMTQEELVSNLGTIARSGS

KAFLDALQNQAEASSKIIGQFGVGFYSAFMVADRVEVYSRSAAPGSLGYQWLSDGSGVFE

IAEASGVRTGTKIIIHLKSDCKEFSSEARVRDVVTKYSNFVSFPLYLNGRRMNTLQAIWM

MDPKDVREWQHEEFYRYVAQAHDKPRYTLHYKTDAPLNIRSIFYVPDMKPSMFDVSRELG

SSVALYSRKVLIQTKATDILPKWLRFIRGVVDSEDIPLNLSRELLQESALIRKLRDVLQQ

RLIKFFIDQSKKDAEKYAKFFEDYGLFMREGIVTATEQEVKEDIAKLLRYESSALPSGQL

TSLSEYASRMRAGTRNIYYLCAPNRHLAEHSPYYEAMKKKDTEVLFCFEQFDELTLLHLR

EFDKKKLISVETDIVVDHYKEEKFEDRSPAAECLSEKETEELMAWMRNVLGSRVTNVKVT

LRLDTHPAMVTVLEMGAARHFLRMQQLAKTQEERAQLLQPTLEINPRHALIKKLNQLRAS

EPGLAQLLVDQIYENAMIAAGLVDDPRAMVGRLNELLVKALERH

>hsp90ab1 Mus musculus

MPEEVHHGEEEVETFAFQAEIAQLMSLIINTFYSNKEIFLRELISNASDALDKIRYESLT

DPSKLDSGKELKIDIIPNPQERTLTLVDTGIGMTKADLINNLGTIAKSGTKAFMEALQA
G

ADISMIGQFGVGFYSAYLVAEKVVVITKHNDDEQYAWESSAGGSFTVRADHGEPIGR
GTK

VILHLKEDQTEYLEERRVKEVVKKHSQFIGYPITLYLEKEREKEISDDEAEEEKGEKEE
E

DKEDEEKPKIEDVGSDEEDDSGKDKKKKTKKIKEKYIDQEELNKTKPIWTRNPDDITQ
EE

YGEFYKSLTNDWEDHLAVKHFSVEGQLEFRALLFIPRRAPFDLFENKKKKNNIKLYVR
RV

FIMDSCDELIPEYLNFIRGVVDSEDLPLNISREMLQQSKILKVIRKNIVKKCLELFSELA

EDKENYKKFYEAFSKNLKLGIHEDSTNRRRLSELLRYHTSQSGDEMTSLSEYVSRMK
ETQ

KSIYYITGESKEQVANSAFVERVRKRGFEVVYMTEPIDEYCVQQLKEFDGKSLVSVTK
EG

LELPEDEEEKKKMEESKAKFENLCKLMKEILDKKVEKVTISNRLVSSPCCIVTSTYGW
TA

NMERIMKAQALRDNSTMGYMMAKKHLEINPDHPIVETLRQKAEADKNDKAVKDLV
VLLFE

TALLSSGFSLEDPQTHSNRIYRMIKLGLGIDEDEVTAEEPSAAVPDEIPPLEGDEDASR
M

EEVD

>hsp90b1 Mus musculus

MRVLWVLGLCCVLLTFGFVRADDEVDVDGTVEEDLGKSREGSRTDDEVVQREEEAI
QLDG

LNASQIRELREKSEKFAFQAEVNRMMKLIINSLYKNKEIFLRELISNASDALDKIRLISL

TDENALAGNEELTVKIKCDKEKNLLHVTDTGVGMTREELVKNLGTIAKSGTSEFLNK
MTE

AQEDGQSTSELIGQFGVGFYSAFLVADKVIVTSKHNNDTQHIWESDSNEFSVIADPRG
NT

LGRGTTITLVLKEEASDYLELDTIKNLVRKYSQFINFPIYVWSSKTETVEEPLEEDEAA
K

EEKEESDDEAAVEEEEEEKKPKTKKVEKTVWDWELMNDIKPIWQRPSKEVEEDEYK
AFYK

SFSKESDDPMAYIHFTAEGEVTFKSILFVPTSAPRGLFDEYGSKKSDYIKLYVRRVFITD

DFHDMMPKYLNFVKGVVDSDDLPLNVSRETLQQHKLLKVIRKKLVRKTLDMIKKIA
DEKY

NDTFWKEFGTNIKLGVIEDHSNRTRLAKLLRFQSSHHSTDITSLDQYVERMKEKQDKI
YF

MAGSSRKEAESSPFVERLLKKGYEVIYLTEPVDEYCIQALPEFDGKRFQNVAKEGVKF
DE

SEKTKESREATEKEFEPLLNWMKDKALKDKIEKAVVSQRLTESPCALVASQYGWSGN
MER

IMKAQAYQTGKDISTNYYASQKKTFEINPRHPLIRDMLRRIKEDEDDKTVMDLAVVLF
ET

ATLRSGYLLPDTKAYGDRIERMLRLSLNIDPEAQVEEEPEEEPEDTSEDAEDSEQDEGE
E

MDAGTEEEEEETEKESTEKDEL

>trap1 Mus musculus

MACELRAVLLWGRGLQTVLRAPALAGVRRGKPVLHLQKTTVQFRGPTQSLASGISA
GQLY

STQAAEDKEEESLHSIISNTEAVRGSVSKHEFQAETKKLLDIVARSLYSEKEVFIRELIS

NASDALEKLRHKLVCEGQVLPEMEIHLQTDAKKGTITIQDTGIGMTQEELVSNLGTIA
RS

GSKAFLEALQNQAETSSKIIGQFGVGFYSAFMVADKVEVYSRSAAPESPGYQWLSDG
SGV

FEIAEASGVRPGTKIIIHLKSDCKDFASESRVQDVVTKYSNFVSFPLYLNGKRINTLQAI

WMMDPKDISEFQHEEFYRYIAQAYDKPRFTLHYKTDAPLNIRSIFYVPEMKPSMFDVS
RE

LGSSVALYSRKVLIQTKAADILPKWLRFIRGVVDSEDIPLNLSRELLQESALIRKLRDV
L

QQRLIKFFIDQSKKDAEKYAKFFEDYGLFMREGIVTTAEQDIKEDIAKLLRYESSALPA
G

QLTSLPDYASRMQAGTRNIYYLCAPNRHLAEhspYYEAMKQKHTEVLFCYEQFDELTL
LH

LREFDKKKLISVETDIVVDHYKEEKFEDTSPADERLSEKETEDLMAWMRNALGSRVT
NVK

VTFRLDTHPAMVTVLEMGAARHFLRMQQLAKTQEERAQLLQPTLEINPRHTLIKKLC
QLR

ESEPELAQLLVDQIYENAMIAAGLVDDPRAMVGRLNDLLVKVLEKH

>hsp90ab1 Gallus gallus

MPEQVQHGEDEVETFAFQAEIAQLMSLIINTFYSNKEIFLRELISNASDALDKIRYESLT
DPSKLDTGKD

LKIDIVPNPRDRTLTLLDTGIGMTKADLVNNLGTIAKSGTKAFMEALQAGADISMIGQ
FGVGFYSAYLVA

EKVVVITKHNDDEQYAWESSAGGSFTVRTDHGEPIGRGTKVILYLKEDQTEYLEERRV
KEVVKKHSQFIG

YPITLYVEKEREKEISDDEAEEEKAEKEEEESKDEEKPKIEDVGSDEEEEGEKSKKKKT
KKIKEKYIDQE

ELNKTKPIWTRNPDDITQEEYGEFYKSLTNDWEDHLAVKHFSVEGQLEFRALLFIPRR
APFDLFENKKKK

NNIKLYVRRVFIMDSCDELIPEYLNFIRGVVDSEDLPLNISREMLQQSKILKVIRKNIVK
KCLELFTELA

EDKENYKKFYEAFSKNLKLGIHEDSTNRKRLSELLRYHTSQSGDEMTSLSEYVSRMK
ESQKSIYYITGES

KEQVANSAFVERVRKRGFEVVYMTEPIDEYCVQQLKEFDGKTLVSVTKEGLELPEDE
EEKKKMEESKAKF

ETLCKLMKEILDKKVEKVTISNRLVSSPCCIVTSTYGWTANMERIMKAQALRDNSTM
GYMMAKKHLEINP

DHPIVETLRQKADADKNDKAVKDLVVLLFETALLSSGFSLEDPQTHSNRIYRMIKLGL
GIDEDEVIAEES

SIAPPDEIPPLEGDEDTSRMEEVD

>hsp90b1 Gallus gallus

MKSAWALALACTLLLAASVTAEEVDVDATVEEDLGKSREGSRTDDEVVQREEEAIQL DGLNASQIKEIRE

KSEKFAFQAEVNRMMKLIINSLYKNKEIFLRELISNASDALDKIRLISLTDENALAGNE ELTVKIKCDKE

KNMLHVTDTGIGMTKEELIKNLGTIAKSGTSEFLNKMTEMQDDSQSTSELIGQFGVG FYSAFLVADRVIV

TSKHNNDTQHIWESDSNEFSVIDDPRGNTLGRGTTITLVLKEEASDYLELDTVKNLVK KYSQFINFPIYV

WSSKTETVEEPVEEEEAKEEKEETDDDEAAVEEEEEEKKPKTKKVEKTVWDWELMN DIKPIWQRPSKEVE

EDEYKAFYKTFSKEHDDPMAYIHFTAEGEVTFKSILFVPNSAPRGLFDEYGSKKSDFIK LYVRRVFITDD

FHDMMPKYLNFVKGVVDSDDLPLNVSRETLQQHKLLKVIRKKLVRKTLDMIKKIAE EKYNDTFWKEFGTN

VKLGVIEDHSNRTRLAKLLRFQSSHHESNLTSLDQYVERMKEKQDKIYFMAGASRKE AESSPFVERLLKK

GYEVIYLTEPVDEYCIQALPEFDGKRFQNVAKEGVKFEESEKSKESREALEKEFEPLLN WMKDKALKDKI

EKAVLSQRLTQSPCALVASQYGWSGNMERIMKAQAYQTGKDISTNYYASQKKTFEIN PRHPLIKDMLRRV

KENEDDKTVSDLAVVLFETATLRSGYMLPDTKEYGDRIERMLRLSLNIDLDAKVEEEP EEPEDAAEEAEQ

DEEEVDADAEDSETQKESTDVKDEL

>trap1 Gallus gallus

MASSAKGPPCLQREVLCSQRLPHRIPAACISACRAYSTQTAESKEEEPLHTIISNTENVK GAASKHEFQA

ETKKLLDIVARSLYSEKEVFIRELISNGSDALEKLRHRLMAEGKALPEMEIHLQTDSGK GTITIQDTGIG

MTQEELVSNLGTIARSGSKAFLDALQSQAEASSKIIGQFGVGFYSAFMVADKVEVFSQ SAEPGSLGYHWS

SDGSGMFEIAEASGVRTGTKIIIHLKEDCKEFANEDRVKEVVTKYSNFISFPLYLNGRRI
NTLQALWMLD

PKDIGEWQHEEFYRFIAQAYDKPRYILHYKTDAPLNIRSIFYVPEQKPSMFDISRELGSS
VALYSRKILI

QTKAADILPKWLRFLRGVVDSEDIPLNLSRELLQESALIRKLRDVLQKRLIKFFVDQS
KKDPEKYAKFFE

DYGVFMREGIVTIAEQDVKEDIAKLLRYESSALPAGQLTSLTEYASRMKAGSRNIYYL
CAPNRHLAEHSP

YFEAMKKKDMEVLFCYEQFDELTLLHLREFDKKKLISVETDIVVDHYKEEKFEESRP
AAERLTDVEAEDL

MAWMRNALGSRVTGVKVTTRLDTHPAMITVLEMGAARHFLRMQQLAKTQEERAQL
LQPTLEINTGHTLIK

KLNELKDSQPDLAQMLLDQIYENAMIAAGLNEDPRPMVSRLNELLTKILEKN

>hsp90ab1 Danio rerio

MPEEMRQEEEAETFAFQAEIAQLMSLIINTFYSNKEIFLRELVSNASDALDKIRYESLTD

PTKLDSGKDLKIDIIPNVQERTLTLIDTGIGMTKADLINNLGTIAKSGTKAFMEALQAG
A

DISMIGQFGVGFYSAYLVAEKVTVITKHNDDEQYAWESSAGGSFTVKVDHGEPIGRGT
KV

ILHLKEDQTEYIEEKRVKEVVKKHSQFIGYPITLYVEKERDKEISDDEAEEEKAEKEEK
E

EEGEDKPKIEDVGSDDEEDTKDKDKKKKKKIKEKYIDQEELNKTKPIWTRNPDDISNE
EY

GEFYKSLTNDWEDHLAVKHFSVEGQLEFRALLFIPRRAPFDLFENKKKKNNIKLYVRR
VF

IMDNCEELIPEYLNFIRGVVDSEDLPLNISREMLQQSKILKVIRKNIVKKCLELFAELAE

DKDNYKKFYDAFSKNLKLGIHEDSQNRKKLSELLRYQSSQSGDEMTSLTEYVSRMKE
NQK

SIYYITGESKDQVAHSAFVERVCKRGFEVLYMTEPIDEYCVQQLKDFDGKSLVSVTKE
GL

ELPEDEDEKKKMEEDKAKFENLCKLMKEILDKKVEKVTVSNRLVSSPCCIVTSTYGW

TAN

MERIMKAQALRDNSTMGYMMAKKHLEINPDHPIMETLRQKAEADKNDKAVKDLVI
LLFET

ALLSSGFSLDDPQTHSNRIYRMIKLGLGIDEDEDVPVEEPSSAAAPEDIPPLEGDDDAS
R

MEEVD

>hsp90b1 Danio rerio

MRRLWIIGLLCALLAFASVKADDDVDIDGTVEEDLGKSRDGSRTDDEVVQREEEAIQ
LDG

LNTSQLKEIRDKAEKHAFQAEVNRMMKLIINSLYKNKEIFLRELISNASDALDKIRLLS
L

TNEDALAGNEELTIKIKSDKEKNMLHITDTGIGMTKEELVKNLGTIAKSGTSEFLNKM
TE

VQDDSQSTSELIGQFGVGFYSAFLVADKVIVTSKHNNDTQHMWESDSNQFSVIEDPR
GDT

LGRGTTITLVMKEEASDYLELETIKNLVKKYSQFINFPIYVWSSKTETVEEPIEDEAEA
E

KEEATEDEAEVEEEEEDKDKPKTKKVEKTVWDWELMNDIKPIWQRPAKEVEEDEYT
AFYK

TFSRDTDEPLSHIHFTAEGEVTFKSILFVPASAPRGLFDEYGTKKNDFIKLFVRRVFITD

DFHDMMPKYLNFIKGVVDSDDLPLNVSRETLQQHKLLKVIRKKLVRKTLDMIKKIAE
EQY

NDKFWKEFGTNIKLGVIEDHSNRTRLAKLLRFQTSHSDTVLSSLEQYVERMKEKQDK
IYF

MAGTSRKEAESSPFVEKLLKKGYEVVYLTEPVDEYCIQALPEFDGKRFQNVAKEGVK
FDE

SDKAKEKREALEKEFEPLTTWMKDKALKEQIEKAVLSQRLTNSPCALVASQYGWSGN
MER

IMKAQAYQTGKDISTNYYASQKKTLEINPKHPLIKEMLRRVNEDAEDKTAADLAVVL
FET

ATLRSGYQLQDTKAYGERIERMLRLSMNVDLDAQVEEEPEEEPEEQTEEAEDEEEVQ

ADE

AEEESEATSKDEL

>trap1 Danio rerio

MIRSLNLLKYSLNVSQRAQLRLLSSSKWTGHRIVPAAFTGVDTNGRQQLHQPRPFRL
WSPSCSGVSVHHR

SYSTQQHTEPAEEETLHNIITDTENVQGSFSKHEFQAETKKLLDIVARSLYSEKEVFIRE
LISNGSDALE

KLRHRMITAGGDTAPMEIHLQTDSVKGTFTIQDTGVGMNKEDLVSNLGTIARSGSKA
FLDALQNQAEASS

SIIGQFGVGFYSAFMVADKVEVYSQSAEADAPGYKWSSDGSGVFEVAEASGVRQGTK
IVLHLKDDCKEFS

SEDRVKEVVTKYSNFVSFPIFLNGRRLNTLQALWMMEPKDISEWQHEEFYRYVAQAY
DKPRYTLHYRADA

PLNIRSIFYVPEMKPSMFDVSREMGSSVALYSRKILIQTKATDILPKWLRFLRGVVDSE
DIPLNLSRELL

QESALIRKLRDVLQQRVIRFLLDQSKKDPEKYARFFEDYGLFMREGIVTTGEQSVKED
IAKLLRFESSAL

PAGQQTSLMEYSSRMKAGTRNIYYLCAPNRHLAEHSPYFEAMKQKDMEVLFCFEQF
DELTLLHLREFDRK

KLISAETDIVVDHYKEEKFQDSKPASERLSSEQAEDLLAWMRNALVQRVTNIKVTPRL
DTHPAMITVLEM

GAARHFLRTQQLARSSEERAQILQPTLEINTGHDLIKKLHALKDSNPELAQLLLEQIYD
NAMIAAGLNED

PRPMISRLNQLLTRALEKH

>hsp90b1 Oryzias latipes

MKRLWVVGLLCALLAFAAVRAEDDEVDVDATVEEELGKSRDGSRTDDEVVQREEEA
IQLD

GLNAAQIKELREKSEKHMFQAEVNRMMKLIINSLYKNKEIFLRELISNASDALDKIRL
LS

LTHEDALAANEELTIKIKSDKEKNMLHITDTGIGMTKEELIKNLGTIAKSGTSEFLNKM
S

EMQSEGQSTSELIGQFGVGFYSAFLVADKVIVSSKHNNDTQHIWESDSNQFSVIEDPR
GD

TLGRGTTITLVLKEEASDFLELETIKNLVRKYSQFINFPIYVWASKTETVEEPIEEDAET

EEPEKEASEDEVEVEEEEGEDKDKPKTKKVEKTVWDWELMNDIKPIWQRPSKEVEE
DEYK

AFYKTFSKDSEDPLTHIHFTAEGEVTFKSILFVPSAAPRGLFDEYGSKKNDFVKLFVRR
V

FITDDFNDMMPKYLNFVKGVVDSDDLPLNVSRETLQQHKLLKVIRKKLVRKTLDMIK
KIA

EEQYNEKFWKEFGTNIKLGVIEDHSNRTRLAKLLRFQTSNSETGLASLEQYVERMKE
KQD

KIYFMAGTSRKEAESSPFVEKLLKKGYEVIYLTEPVDEYCIQALPEFDGKRFQNVAKE
GV

KFEESEKAKEKREALEKEFEPLMTWLKDKALKDKIEKAVLSQRLTDSPCALVASQYG
WSG

NMERIMKAQAYQTGKDISTSYYASQKKTLEINPKHPLIKQMLGRVNDDAEDQTASDL
AVV

LFETATLRSGYQLADTKAYGDRIERMLRLSMNVPLDEQVEEEPEEEPEEQAEDDSEAK
EE

VVDDDDEDETTEKDEL

>trap1 Oryzias latipes

MSRFLGLCRFGLSSLQRSNSRVMSRGLSSRTMSLPLARDLQALQKRHCQPKLCRLQG
SCH

VLSQQSFYSTQEVEKEPEVEPLHTIISDTESVQGSFSKHEFQAETKKLLDIVARSLYSEK

EVFIRELISNGSDALEKLRHKLITAGGETAPMEIHLKTDAGKGTITIQDTGVGMNKEEL
V

ANLGTIARSGSKAFLDALQNQAEASSSIIGQFGVGFYSAFMVADRVDVFTRSADRDAP
GY

KWSSDGSGLFEVAEASGVQQGTKIVLHLKDDCKEFSSEDRVKEVVTKYSNFVSFPIFL
NG

RRLNTLQALWLMEPKEISDWQHEEFYRYIAQAYDKPRYTLHYRADAPLNIRSIFYVPD

SK

PSMFDVSREMGSSVALYSRKVLIQTKASDILPKWLRFLRGVVDSEDIPLNLSRELLQES
A

LIRKLRDVLQQRVIRFLLDQSKKEPEKYNTFFEDYGLFMREGIVTTQEQDIKEDIAKLL
R

FESSALPAGQQTNLMEYGSRMKAGTRNIYYLCAPNRHLAEHSPYFEAMKKKDMEVL
FCYE

QFDELTLLHLREFDKKKLISVETDIMVDHYKEEKFEDSKPASERLTQEQADDLMAWM
KNA

LGPRVTNIKLTPRLDTHPAMITVLEMGAARHFLRTQQLARSAEERAQILQPTLEINAGH
D

LIKKLHTLKDSNSELAVLLLEQVYDNAMIAAGLNDDPRPMIARLNDLLTKALEKH

>hsp90ab1 Larimichthys crocea

MPEEMHQEEEAETFAFQAEIAQLMSLIINTFYSNKEIFLRELISNASDALDKIRYESLTE
PTKLDSGKDL

KIDIIPNKDDRTLTLIDTGIGMTKADLINNLGTIAKSGTKAFMEALQAGADISMIGQFG
VGFYSAYLVAE

KVVVITKHNDDEQYAWESSAGGSFTVKVDSGEPIGRGTKIILHLKEDQTEYIEEKRVK
EIVKKHSQFIGY

PITLFVEKERDKEISDDEAEEERAEKEEKEEGEDKPKIEDVGSDDEEDSKDKDKKKKK
KIKEKYIDQEEL

NKTKPIWTRNPDDITNEEYGEFYKSLTNDWEDHLAVKHFSVEGQLEFRALLFIPRRAP
FDLFENKKKKNN

IKLYVRRVFIMDNCEELIPEYLNFVRGVVDSEDLPLNISREMLQQSKILKVIRKNIVKK
CLELFAELAED

KENYKKFYEGFSKNIKLGIHEDSQNRKKLSELLRYHSSQSGDETTSLTEYLTRMKENQ
KSIYYITGESKD

QVANSAFVERVRKRGFEVLYMTEPIDEYCVQQLKEFDGKSLVSVTKEGLELPEDEEEK
KKMEEDKAKFES

LCKLMKEILDKKVEKVTVSNRLVSSPCCIVTSTYGWTANMERIMKAQALRDNSTMG
YMMAKKHLEINPDH

PIVETLRQKADADKNDKAVKDLVILLFETALLSSGFSLDDPQTHSNRIYRMIKLGLGID
DDDVPTEEAVT

TAVPDEIPPLEGDGDDDASRMEEVD

>hsp90b1 Larimichthys crocea

MKRVWAIGLLFALLAFAAVKAEEDELDIDGTVEEDIGKSRDGSRTDDEAVQREEEAIQ
LDGLNAAQIKEI

REKSEKHVFQAEVNRMMKLIINSLYKNKEIFLRELISNASDALDKIRLLSLTNEDAMA
ANEELTIKIKSD

KERNMLHITDTGVGMTKDELVRNLGTIAKSGTSEFLNKMTEMQTEGQSTSELIGQFG
VGFYSAFLVADKV

IVTSKHNNGTQHIWESDSNQFSVIEDPRGDTLGRGTTITLVLKEEASDYLELETIKNLV
RKYSQFINFPI

YVWASKTETVEEPIEEDAEAAEEPEKETAEDEAEVEEEEEDKEKPKTKKVEKTVWD
WELMNDIKPIWQRP

AKDVEEDEYKAFYKTFSKDSDDPLAHIHFMAEGEVTFKSILFVPTSAPRGLFDEYGSK
KNDYIKLFVRRV

FITDDFNDMMPKYLNFVKGVVDSDDLPLNVSRETLQQHKLLKVIRKKLVRKTLDMIK
KIAEDQYNDKFWK

EFGTNIKLGVIEDHSNRTRLAKLLRFQTSNSETVLASLEEYVERMKEKQDKIYFMAGT
SRKEAESSPFVE

RLLKKGYEVIYLTEPVDEYCIQALPEFDGKRFQNVAKEGVKFDESDKAKEKRETLEK
EFEPLTTWLKDKA

LKDKIEKAIISQRLTNSPCALVASQYGWSGNMERIMKAQAYQTGKDISTNYYASQKKT
LEINPKHPLVKK

MLTRITSDPEDQTASDLAVVLFETATLRSGYQLADTKAYGDRIERMLRLSMNVDLDEQ
IEEEPEEEPEEA

AEDEDKEDDSEDKEEIVDEDDETETTEKDEL

>trap1 Larimichthys crocea

MSRCLALARFALGTNSRLTLCARGLSRCTISRSVAAGLQVGQQQRWSSQPRVWSSQR
PCLGFSQQSYYST

QEAEKEPEEEPLHTIISDTESVQGSFSKHEFQAETKKLLDIVARSLYSEKEVFIRELISNG

SDALEKLRH

SLITAGGATAPMEIHLQTDGAKGIFTIQDTGVGMNQEELVANLGTIARSGSKAFLDAL QNQTEASSTIIG

QFGVGFYSAFMVADKVDVYSQSAEPGVPGYKWSSDGSGVFEIAEASGVQQGTKIVL HLKDDCKEFSSEDR

VKEVVTKYSNFVSFPIFLNGRRLNTLQALWMMEPKEISDWQHEEFYRYIAQSYDHPR YTLHYRADAPLNI

RSIFYVPDTKPSMFDVSREMGSSVALYSRKVLIQTKATDILPKWLRFLRGVVDSEDIPL NLSRELLQESA

LIRKLRDVLQQRVIRFLLDQSKKDPEKYNKFFEDYGLFMREGIVTTQEQDVKEDIAKL LRFESSALPAGQ

QTNLMEYASRMKAGTRNIYYLCAPNRHLAEHSPYYEAMKQKDMEVLFCYEQFDELT LLHLREFDKKKMIS

AETDIVVDHYKEEKFEETKPASERLTQEQADDLISWMKNSLGPRVTNIKLTPRLDTHP AMITVLEMGAAR

HFLRTQQLARTAEERAQILQPTLEINAGHDLIKKLHELKQSNNELAGLLLEQIYDNAMI TAGLNDDPRPM

ISRLNDLLTKALEKH

>hsp90ab1 Oreochromis niloticus

MQMPEEMHQEEEAETFAFQAEIAQLMSLIINTFYSNKEIFLRELISNASDALDKIRYES L

TDPSKLDSGKDLKIDIIPNKAERTLTLVDTGIGMTKADLINNLGTIAKSGTKAFMEALQ A

GADISMIGQFGVGFYSAYLVAEKVVVITKHNDDEQYAWESSAGGSFTVKVDNSEPIGR GT

KIILYLKEDQTEYVEEKRVKEIVKKHSQFIGYPITLFVEKERDKEVSDDEAEEEKAEKE E

KEEGEDKPKIEDVGSDDEEDSKDKDKKKKKKIKEKYIDQEELNKTKPIWTRNPDDIT NEE

YGEFYKSLTNDWEDHLAVKHFSVEGQLEFRALLFIPRRAPFDLFENKKKKNNIKLYVR RV

FIMDNCEELIPEYLNFIRGVVDSEDLPLNISREMLQQSKILKVIRKNIVKKCLELFAELG

EDKENYKKFYEAFSKNLKLGVHEDSQNRKKLSELLRYHSSQSGDETTSLSEYVTRMK
ENQ

KSIYYITGESKDQVANSAFVERVRKRGFEVLYMTEPIDEYCVQQLKEFDGKTLVSVTK
EG

LELPEDEEEKKKMEDDKAKFENLCKLMKEILDKKVEKVTVSNRLVSSPCCIVTSTYG
WTA

NMERIMKAQALRDNSTMGYMMAKKHLEINPDHPIMETLRQKAEADKNDKAVKDLV
ILLFE

TALLSSGFSLDDPQTHSNRIYRMIKLGLGKKTPDPLDFLEVGIDLGEQLAQCIH

>hsp90b1 Oreochromis niloticus

MTEMQEEGQSTSELIGQFGVGFYSAFLVADKVIVTSKHNNDTQHIWESDSNQFSVIED
PR

GDTLGRGTTITLVLKEEASDYLELETIKNLVKKYSQFINFPIYVWASKTETVEEPIEEDA

EAAEEPEKEASEDEAEVEEEEGEDKDKPKTKKVEKTVWDWELMNDIKPIWQRPAKE
VEED

EYKAFYKTFSKDNDDPMAHIHFTAEGEVTFKSILFVPTSAPRGLFDEYGSKKNDYIKL
FV

RRVFITDDFNDMMPKYLNFIKGVVDSDDLPLNVSRETLQQHKLLKVIRKKLVRKTLD
MIK

KIAEEQYNDKFWKEFGTNIKLGVIEDHSNRTRLAKLLRFQTSNSETDLSSLEQYVERM
KE

KQDKIYFMAGTSRKEAESSPFVERLLKKGYEVIYLTEPVDEYCIQALPEFDGKRFQNV
AK

EGVKFDESEKAKEKREALEKEFEPLTTWLKDKALKDKIEKAVLSQRLTNSPCALVASQ
YG

WSGNMERIMKAQAYQTGKDISTNYYASQKKTLELNPKHPLVKQLLNRVNADAEDQT
ASDL

AVVLFETATLRSGYQLVDTKAYGDRIERMLRLSLNVPLDEQAILVSVSELMVPLPSVTF
F

EFSPVSAF

>trap1 Oreochromis niloticus

ISYVFGARRGLSVGQLQRWSSRPRVWSSQQACLGFSPQSYYSTQEAEKEPEEEPLHNII
S

DTEAVQGSFHKHEFQAETKKLLDIVARSLYSEKEVFIRELISNGSDALEKLRHKLISSG
G

ETAPMEIHLQSDAAKGTFTIQDTGIGMNKDELVSNLGTIARSGSKAFLDALQNQAEAS
ST

IIGQFGVGFYSAFMVADHVDVYTRSSETGAPGYKWSSDGSGVFEIAEATGVQQGTKI
VLH

LKDDCKEFSSEDRVKEVVTKYSNFVSFPIFLNGRRLNTLQALWMMEPKEISDWQHEE
FYR

YIAQAYDKPRYTLHYRADAPLNIRSIFYVPEAKPSMFDVSREMGSSVALYSRKVLIQT
KA

TDILPKWLRFLRGVVDSEDIPLNLSRELLQESALIRKLRDVLQQRVIRFLLDQSKKEPE
K

YSKFFEDYGLFMREGIVTTQEQDVKEDIAKLLRFESSALPAGQQTNLMEYASRMKAG
TRN

IYYLCAPNRHLAEHSPYYEAMKQKDMEVLFCYEQFDELTLLHLREFDKKKLISVETDI
VV

DHYKEEKYEDSKPASERLTQEQADDLMVWMKNSLGPRVTNIKLTPRLDTHPAMITVL
EMG

AARHFLRTQQLARTAEERAQILQPTLEINAGHDLIKKLFALKDTNSELAGLLLEQIYDN
A

MITAGLNDDPRPMISRLNDLLTKALEKH

>hsp90ab1 Salmo salar

MPEEMRQEEEAETFAFQAEIAQLMSLIINTFYSNKEIFLRELISNASDALDKIRYESLTD
PTKLDNGKEL

KIDVIPNVEERTLTLIDTGIGMTKADLINNLGTIAKSGTKAFMEALQAGADISMIGQFG
VGFYSAYLVAE

RVTVITKHNDDEQYIWESSAGGSFTVKVDTGEPMLRGTKVILHMKEDQTEYVEEKRV
KEVVKKHSQFIGY

PITLFVEKEREKEISDDEEEKAEEEKEEKEAEDKPKIEDVGSDDEEDSKDKDKKKTKK
IKEKYIDQEELN

KTKPIWTRNPDDITMEEYGEFYKSLTNDWEEHLAVKHFSVEGQLEFRALLFIPRRAPF
DLFENKKKKNNI

KLYVRRVFIMDSCEELIPEYLNFVRGVVDSEDLPLNISREMLQQSKILKVIRKNIVKKC
MELFGELAEDR

ENYNKFYDGFSKNLKLGIHEDSQNRKKLSELLRYHSSQSGDELTSLTEYLTRMKDNQ
KSIYYITGESKDQ

VANSAFVERVRKRGFEVLYMTEPIDEYCVQQLKEFDGKTLVSVTKEGLELPEDEEEK
KKMDEDKTKFENL

CKLMKEILDKKVEKVP

>hsp90b1 Salmo salar

MNRMWTIGVFCALLAFSSVRAEDEVDVDGTVEEDLGKSRDGSRTDDEVVQREEEAI
QLDGLNAAQVKEIR

EKSEKHVFKAEVNRMMKLIINSLYKNKEIFLRELISNASDALDKIRLLSLTNDEALTGN
EELTVKIKADK

EKNMLHITDTGIGMTKEDLVRNLGTIAKSGTSEFLNKMTEMQTEGQSTSELIGQFGVG
FYSAFLVADKVI

VTTKHNNGTQHIWESDSNEFSVIEDPRGDTLGRGTTITLVMKEEATDYLELETIKNLV
KKYSQFINFPIY

VWSSKTETVEEPIDETEADKDEDHDEVEVEEEEDKPKMKKVEKTVWDWELMNDIKP
IWQRPAKEVEEDEY

KAFYKTFSRDTDEPISHIHFTAEGEVTFKSILFVPAAAPRGLFDEYGSKKNDFIKLFVRR
VVYITDDFHDM

MPKYLNFVKGMVDSDDLPLNVSRETLQQHKLLKVIRKKLVRKTLDMIKKIAEEQYN
EKFWKEFGTNIKLG

VIEDHSNRTRLAKLLRFQTSNSDTVLASLEQYVERMKEKQDKIYFMAGTSRKEAESS
PFVEKLLKRGYEV

VYLTEPVDEYCIQALPEFDGKRFQNVAKEGVKFDESDKTKEKREGLEKEYEPLTTWM
KDSALKDKIEKAV

LSQRLTNSPCALVASQYGWSGNMERIMKAQAYQTGKDISTNYYASQKKTLEINPKHP

LIKEMLKRINDNA

EDQTASDLAVVLFETATLRSGYQLADTKAYGDRIERMLRLSMNVDLNEEPEEEPEEEP
EEELKADEEDED

SGATGRDEL

>trap1 Salmo salar

MSRCLKLFKFALGSSQRANSRLLSSGRGLGSRTVGPFSEDAHISQQQKQWSRNGMW
NPSHPGLGLTQSSY

YSTKEAEKVPLDTDKVLEAEKAPATEKVEEVENIQEAEDKDPEEPLHTIIQDTENVRG
TFSKHEFQAETK

KLLDIVARSLYSEKEVFIRELISNGSDALEKLRHKLITGGGDTAPMEIHLQTDQAKGTF
TIQDTGVGMNQ

EELVANLGTIARSGSKAFLDALQSQAEASSSIIGQFGVGFYSAFMVADRVDVYSQSAEP
SSPAYKWSSDG

SGVFEIAEASGVRPGTKIVLHLKDDCKEFSAEDRVKEVVTKYSNFVSFPIFLNGRRLN
TLQALWMMEPKS

ISEWQHEEFYRYVAQSYDKPRYTLHYRADAPVNIRSIFYVPDVKPSMFDVSREMGSS
VALYSRKVLIQTK

ATEILPKWLRFLRGVVDSEDIPLNLSRELLQESTLIRGEQDVRVRLGGWDRGEQDVRV
SLGGWDRGEQDV

RVRLGGWDRGEQDVRGRAGRQGEAGGLGQGRAGRQGETGGWDRGEQDVREDIGK
LLRFESSALPVGQQTN

LKEYGSRMKAGTRNIYYLCAPNRHLAEHSPYFEAMKQKDMEVLFCYEQFDELTLLH
LREFDRKKLISVET

DIVVDHYKEEKYQDSKPASERLTEEQAEDLISWMRNNLGNRVTNIKVTPRLDTHPAMI
TVLEMGAARHFL

RTQQMARSTEERAQILQPTLEINAGHDLIKKMHSLKESDSALAQLLLEQIYDNAMIAA
GLNDDPRPMIAR

LNDLLTRALEKH

>hsp90ab1 Ictalurus punctatus

MPEEMRQEEEAETFAFQAEIAQLMSLIINTFYSNKEIFLRELISNASDALDKIRYESLTD
PSKLDSGKDL

KIDIIPNKHERTLTIIDTGIGMTKADLINNLGTIAKSGTKAFMEALQAGADISMIGQFGV
GFYSAYLVAE

KVVVITKNNDDEQYAWESSAGGSFTVKVDHGEPIGRGTKVILHLKEDQTEYVEEKRV
KEVVKKHSQFIGY

PITLFVEKERDKEISDDEAEDEKEEKEEKEEEEEGEDKPKIEDVGSDDEEDSSKDKDK
KKKKKIKEKYID

QEELNKTKPIWTRNPDDITNEEYGEFYKSLTNDWEDHLAVKHFSVEGQLEFRALLFIP
RRAPFDLFENKK

KKNNIKLYVRRVFIMDSCEELIPEYLNFIRGVVDSEDLPLNISREMLQQSKILKVIRKNI
VKKCLELFAE

LCEDKDNYKKFYEAFSKNIKLGIHEDSQNRKKLSELLRYHSSQSGDEMTSLSEYLSR
MKDNQKSIYYITG

ESKDQVANSAFVERVRKRGFEVLYMVEPIDEYCVQQLKEFEGKTLVSVTKEGLELPE
DEEEKKKMEEDKA

KFESLCKLMKEILDKKVEKVTVSNRLVSSPCCIVTSTYGWTANMERIMKAQALRDNS
TMGYMMAKKHLEI

NPDHPIMEMLRQKAEADKNDKAVKDLVILLFETALLSSGFSLDDPQTHSNRIYRMIKL
GLGIDDDDVPAD

EPTSAPAPEEIPPLEGDDDASRMEEVD

>hsp90b1 Ictalurus punctatus

MRRLWILGLLCALLVFTSVKADDEADVDGTVEDDLGKSRDGSRTDDEVVQREEEAI
QLDGLNAAQIKELR

EKSEKFAFQAEVNRMMKLIINSLYKNKEIFLRELISNASDALDKIRLLSLTNEDALAGN
EELTVKIKSDK

EKNMLHITDTGIGMTKEELVKNLGTIAKSGTSEFLNRMTEMQTEGQSTSELIGQFGVG
FYSAFLVADKVI

VTSKHNNDTQHIWESDSNEFSVIEDPRGNTLGRGTTITLVMKEEASDYLELETIKNLV
RKYSQFINFPIY

VWSSKTETVEEPIEEEEEAEKEATEDEAEVEEEEEDKDKPKTKKVEKTVWDWELMN
DIKPIWQRPAKEVE

EDEYKAFYKTFSRDTDEPLSHIHFTAEGEVTFKSILFVPAAAPRGLFDEYGSKKNDFIK

LFVRRVFITDD

FHDMMPKYLNFIRGVVDSDDLPLNVSRETLQQHKLLKVIRKKLVRKTLDMIKKIAEE
QYNDKFWKEFGTN

IKLGVIEDHSNRTRLAKLLRFQTSHSESGLASLEQYVERMKEKQDKIFFMAGTSRKEA
ESSPFVEKLLKK

GYEVIYLTEPVDEYCIQALPEFDGKRFQNVAKEGVKFDESEKTKEKREALEKEFEPLT
TWMKEKSLKDKI

EKAVLSQRLTSSPCALVASQYGWSGNMERIMKAQAYQTGKDISTNYYASQKKTLEINP
KHPLIKEMLKRV

NANEEDQTASDLAVVLFETATLRSGYQLADTKAYGDRIERMLRLSMNVGLDEQVEEE
PEEEPEEPEEPAE

EAEDEEVPADDAEETETTEKDEL

>trap1 Ictalurus punctatus

MFRCLTLLRLSLQTRGKASLFSCSRSRSVTATFSEVNASTRTKQYGTKLRHWSPSHFG
VKLTCRSYSTQA

ETAEEEILHNIINDTENVQGSYSKHEFQAETKKLLDIVARSLYSEKEVFIRELISNSSDAL
EKLRHKLIT

RGGDSEPLEIHLQTDAARGILTIQDTGIGMNQEELITNLGTIACSGSKAFLEALQSQAD
TQSSIIGQFGV

GFYSAFMVADKLDVYSQSAEPGIPGYKWSSDGSGVFHIAVASGVRQGTKIVLHLKDE
CKEFSSEDRVKEV

VKKYSNFVSFPIYLNGRRLNTLQALWMMDPKEISDWQHEEFYRYVAKAYDKPRYTL
HYRADAPLNIRSIF

YVPEMKPTMFDVSREMGSSVALYSRKVLIQTKATDILPKWLRFLQGVVDSEDIPLNLS
RELLQESALIRK

LRDVLQQRIIRFLLDQSKKEPEKYARFFEDYGLFMREGIVTSAEQDIKEDIAKLLRFES
SALPAGQQTSL

MDYASRMKAGTRNIYYLCAPNRHLAEHSPYFEAMKMKDMEVLFCYEQFDELTLLHL
REFDKKQLMSVETD

IVVDHYKEEKFEASKLASEQLSEQHAEDLMAWMRNCLGQKVTNIKVTPRLNTHPAM
ITVLEMGALRHFLR

TQQLARTVEERAQILQPTLEINAGHDLIRKLHVLKDSNPELAQLLLEQIFDNAMITAGL
NDDPRPMISRL

NELLTKALEKH

>hsp90ab1 Lepisosteus oculatus

MPEEMRQEEEAETFAFQAEIAQLMSLIINTFYSNKEIFLRELISNASDALDKIRYESLTD
PSKLESGKDL

KIDVIPNKNERTLTLIDTGIGMTKADLINNLGTIAKSGTKAFMEALQAGADISMIGQFG
VGFYSAYLVAE

KVVVITKHNDDEQYAWESSAGGSFTVKVDHGEPIGRGTKVILHLKEDQTEYIEEKRV
KEVVKKHSQFIGY

PITLFVEKEREKEISDDEAEEEKTEKEEEKEDADKPKIEDVGSDDEEESADKKKKKKI
KEKYIDQEELNK

TKPIWTRNPDDITTEEYGEFYKSLTNDWEDHLAVKHFSVEGQLEFRALLFVPRRAPFD
LFENKKKKNNIK

LYVRRVFIMDSCEELIPEYLNFVRGVVDSEDLPLNISREMLQQSKILKVIRKNIVKKCL
ELFAELAEDKE

NYKKFYDAFSKNLKLGIHEDSQNRKKLSELLRYHSSQSGDEMTSLTEYISRMKENQN
SIYYITGESKDQV

ANSAFVERVRKRGFEVLYMIEPIDEYCVQQLKEFDGKTLVSVTKEGLELPEDEVEKKK
MEEDKAKFENLC

KLMKDILEKKVEKVTVSNRLVSSPCCIVTSTYGWTANMERIMKAQALRDNSTMGYM
MAKKHLEINPDHPI

VETLRQKAEADKNDKAVKDLVVLLFETALLSSGFSLEDPQTHSNRIYRMIKLGLGIDD
DDVPIEEPSSAP

VPEEIPPLEGEDDASRMEEVD

>hsp90b1 Lepisosteus oculatus

MKKLWMVGLLCALLAFTSVRASDEELDVDGAVEEDLGKSRDGSRTDDEVVQREEEA
IQLDGLNASQIKEL

REKSEKHVFQAEVNRMMKLIINSLYKNKEIFLRELISNASDALDKIRLLSLTDDDALAG
NEELTIKIKSD

KEKNMLHITDTGIGMTKEDLVKNLGTIAKSGTSEFLSKITEMQTEGQSTSELIGQFGV

GFYSAFLVADRV

IVSSKHNNGTQHIWESDSNEFSVIEDPRGDTLGRGTTITLVMKEEASDFLELDTIKNLV
RKYSQFINFPI

YVWSSKTETVEEPIDEEEAADSDKKETTDEEAEAEVEEEEEEKEKPKTKKVEKTVWD
WELMNDIKPIWQR

PAKEVEEEEYKAFYKSFSRDSDEPLSHIHFTAEGEVTFKSILFVPSAAPRGLFDEYGSK
KNDFIKLFVRR

VFITDDFHDMMPKYLNFIKGVVDSDDLPLNVSRETLQQHKLLKVIRKKLVRKTLDMI
KKIAEETYNDKFW

KEFGTNIKLGVIEDHSNRTRLAKLLRFQTSHSESGFSSLEQYTERMKEKQDKIYFMAG
TSRKEAESSPFV

EKLLKKGYEVIYLTEPVDEYCIQALPEFDGKRFQNVAKEGVKFEESETGKEKREALEK
EYEPLTTWMKEK

ALKDKIEKAVLSQRLTDSPCALVASQYGWSGNMERIMKAQAYQTGKDISTNYYASQK
KTFEINPRHPLIK

EMLRRVKDNAEDETASDLAVVLFETATLRSGYQLVDTKAYGDRIERMLRLSMNVDLA
EAVEEEPEEEPEE

PGEEEDAEEEVKAEDEETTSSEKDEL

>trap1 Lepisosteus oculatus

MHDKKNSYRRHLQLWLPVLTLVTKETLKHSLLNPKYLKTHVCPQFKLLTAVYSQPGT
VVAELLLCFSIPC

TWRCCCPAQASSLKGVCAVLGRCAFPDSSAVTPRIFRGPPRVLSVQRQPTLPGRALAW
RAHSTQTAESSE

EEPLHNIISDTEDVQGYCISIESSRYLHLLVILPSFALLLVQCSSHPLPPSLQDTGIGMTK
EELVSNLGT

IARSGSKAFLEALQNQAEASSKIIGQFGVGFYSAFMVADRVDVFSQCAEPGSTGYKW
CSDGSGVFEIAEA

KGVRQGTKIVLHLKDDCKEFASEDRVKEVVTKYSNFVSFPIFLNGRRLNTLQALWM
MDPKEISEWQHEEF

YRYVAQAYDKPRYVVHYRTDAPLNIRSVFYVPEMKPSMFDVSREMGSSVALYSRKVL
IQTKATDILPKWL

RFLRGVVDSEDIPLNLSRELLQESALIRKLRDVLQRRLIKFFVDQSKKEPEKYSKFFED
YGLFMREGIVT

TAEQDVKEDIAKLLRYESSALPAGQQTSLQDYASRMKAGTRNIYYLCAPNRHLAEHS
PYFEAMRKKDMEV

LFCFEQFDELTLLHLREFDKKKLISVETDIVVDHYKEEKFEDSKPASERLPEKQAEDL
MSWMRNVLGQRV

TNIKVTPRLDTHPAMITVLEMGAARHFLRTQQLAKSSEERAQILQPTLEINAGHDLIKK
LNELKDSDPDL

AQLLLEQIYDNAMIAAGLNDDPRPMIGRLNELLTRALEKH

>hsp90ab1 Thamnaconus septentrionalis

MPEELQHEEEGETFFFQAEIAQLMSLIINTFYSNKEIFLRELISNASDALDKIRYESLTE

PTKLDSGKDLKIDVIPDKATNTLTLIDTGIGMTKADLINNLGTIAKSGTKAFMEALQA
GA

DISMIGQFGVGFYSAYLVAEKVVVITKHNDDEQYIWESSAGGSFTVAIDKNESIGRGT
KI

ILHLKEDQAEYLEEKRVKEIVKKHSQFIGYPITLFVEKERDKEISDDEAEEEKADEEEE
K

EDGENKPKIEDVGAEEDEDSKDKKKTKKIKEKYIDQEELNKTKPIWTRNPDDITNEEY
GE

FYKSLTNDWEDHLAVKHFSVEGQLEFRALLFIPRRAPFDLFENKKKKNNIKLYVRRVF
IM

DNCEELIPEYLNFVRGVVDSEDLPLNISREMLQQSKILKVIRKNIVKKCLELFAELTED
K

ENYKKFYEGFSKNIKLGIHEDSQNRKRLSELLRYHSSQCGDETTSLTEYITRMKENQK
SI

YYITGESKDQVANSAFVERVRKRGFEVLYMTEPIDEYCVQQLKEFDGKTLVSVTKEA
LEL

PEDEEEKKKMEEDKAKFEELCKLMKEILDKKVEKVTVSNRLVTSPCCIVTSTYGWTA
NME

RIMKAQALRDNSTMGYMMAKKHLEINPDHAIVATLRMKADADKNDKAVKDLVILLF
ETAL

LSSGFSLDDPQTHSNRIYRMIKLGLGIDDDDVPTEEATSTSVPDEIPPLEGEGEDDASR
M

EEVD

>hsp90b1 Thamnaconus septentrionalis

MKRVWAIGLLFALLAFAAVRAEDEVDVDGTVEDDLGKSRDGSRTDDEVVQREEEAIQ
LDG

LNAAQVKELREKSEKHVFQAEVNRMMKLIINSLYKNKEIFLRELISNASDALDKIRLL
SL

TNDDALAANEELTIKIKSDKEKNMLHITDTGIGMTKDELVRNLGTIAKSGTSEFLNKM
TE

MQTEGQSTSELIGQFGVGFYSAFLVADKVIVTSKHNNGTQHIWESDSNQFSVIEDPRG
DT

LGRGSTVTLVLKEEASDYLELETIKNLVKKYSQFINFPIYVWASKTETVEEPIEEDAEA
T

EEPEKEAEEDEVEVEEEEEDKDKDKPKTKKVEKTVWDWELMNDIKPIWQRSAKEVE
EDEY

TAFYKTFSKDSDDPLAHIHFTAEGEVTFKSILFVPKSAPRGLFDEYGSKKNDFIKLFVR
R

VFITDDFNDMMPKYLNFVKGVVDSDDLPLNVSRETLQQHKLLKVIRKKLVRKTLDMI
KKI

AEEEYDEKFWKEFGTNIKLGVIEDHSNRTRLAKLLRFQTSNSDTVLASLEQYVERMK
EKQ

DKIYFMAGTSRKEAESAPFVERLLKKGYEVIYLTEPVDEYCIQALPEFDGKRFQNVAK
EG

IKFDESEKAKEKRENLEKEFEPLTTWLKDKALKDKIEKAVISQRLTKSPCALVASQYG
WS

GNMERIMKAQAYQTGKDISTNYYASQKKTLEINPKHEIIKWMLQHVNADAEDKAAS
STAE

LLFDTATLRSGYQLVDTKSFSENIEFMLRSSMNIDLNEQIEEEPEDEPADDDAEDKEDD
S

EGKEEVVDEDEDEAKTDKDEL

>trap1 Thamnaconus septentrionalis

MSRCLALARFALGSSKRAYPRIVSCSRGLSTRTVPFSVNGDSKLGQHQRWSSGPRLWS
SQ

RSCLGLIQQSYYSTQEAEKEPEEEPLHNIITDSESVEGEFSKHEFQAETKKLLDIVARSL

YSEKEVFIRELISNGSDALEKLRHKLVTAGGETAQMEIHLQSDGALGTFTIQDTGMGM
NK

EELVSNLGTIARSGSKAFLDALQNQAEASSTIIGQFGVGFYSAFMVADRVDVYSKAAE
PG

APGYKWSSDGSGVFEIAEANGVQQGTKIVLHLKDDCKEFSSEDRVKEVVTKYSNFVS
FPI

FLNGRRLNTLQALWMMEPKEISDWQHEEFYRYVAQTFDKPRYTLHYRADAPLNIRSI
FYV

PDAKPTMFDVSREMGSSVALYSRKVLIQTKATDILPKWLRFLRGVVDSEDIPLNLSRE
LL

QESALIRKLRDVLQQRVIRFLLDQSKKDPEKYRAFFEDFGLFMREGIVTTQEQDVKED
IA

KLLRYESSALPAGQHTSLMEYASRMKAGTRNIHYLCAPNRHLAEHSPYYEAMKQKD
MEVL

FCYEQFDELTLLHLREFDKKKLISVETDIVVDHYKEETFEDSKPASERLTQEQADDLTA
W

MKNTLGPRVTNVKLTPRLDTHPAMITVLEMGAARHFLRTQQLARTPEERAQILQPTLE
IN

AGHDLIKKLYVLKDTNSELAGLLLEQIYDNAMITAGLNDDPRPMISRLNDLLTKAME
KH
