# Supplementary material for: Genome-Wide Identification, Molecular Characterization, and Expression Analysis of the HSP70 and HSP90 Gene Families in Thamnaconus septentrionalis
Source: Int J Mol Sci. 2024 May 24;25(11):5706. doi: 10.3390/ijms25115706 (PMC11172388; doi:10.3390/ijms25115706)
Supplement: Supplementary file 1 [file ijms-25-05706-s001.zip › Table S4-Validated 3D structures of HSP70 and HSP90 proteins produced by SAVES v6.0 website.pdf]

1. *hsp70:*

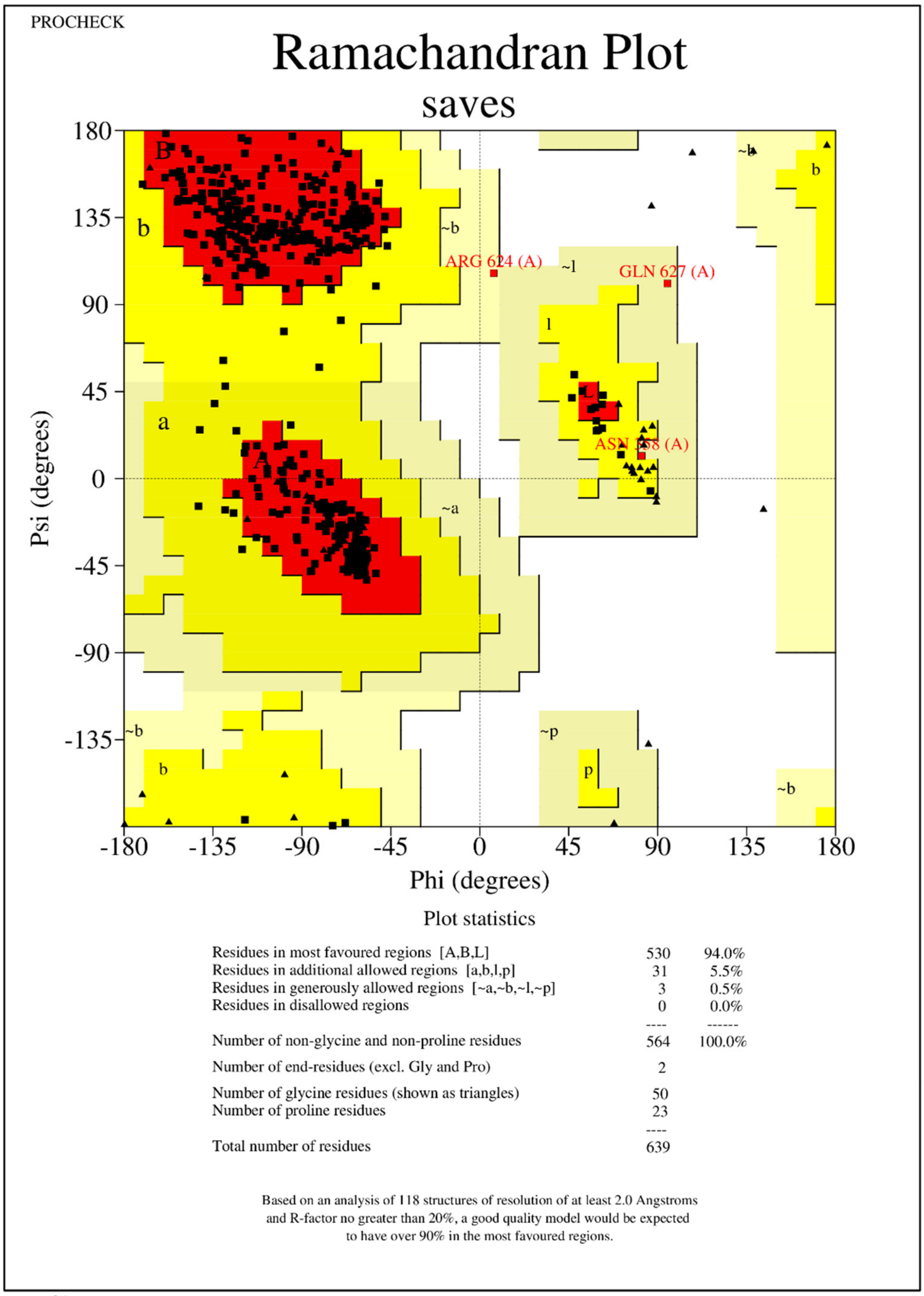

saves_01.ps

2. *hspa1b:*

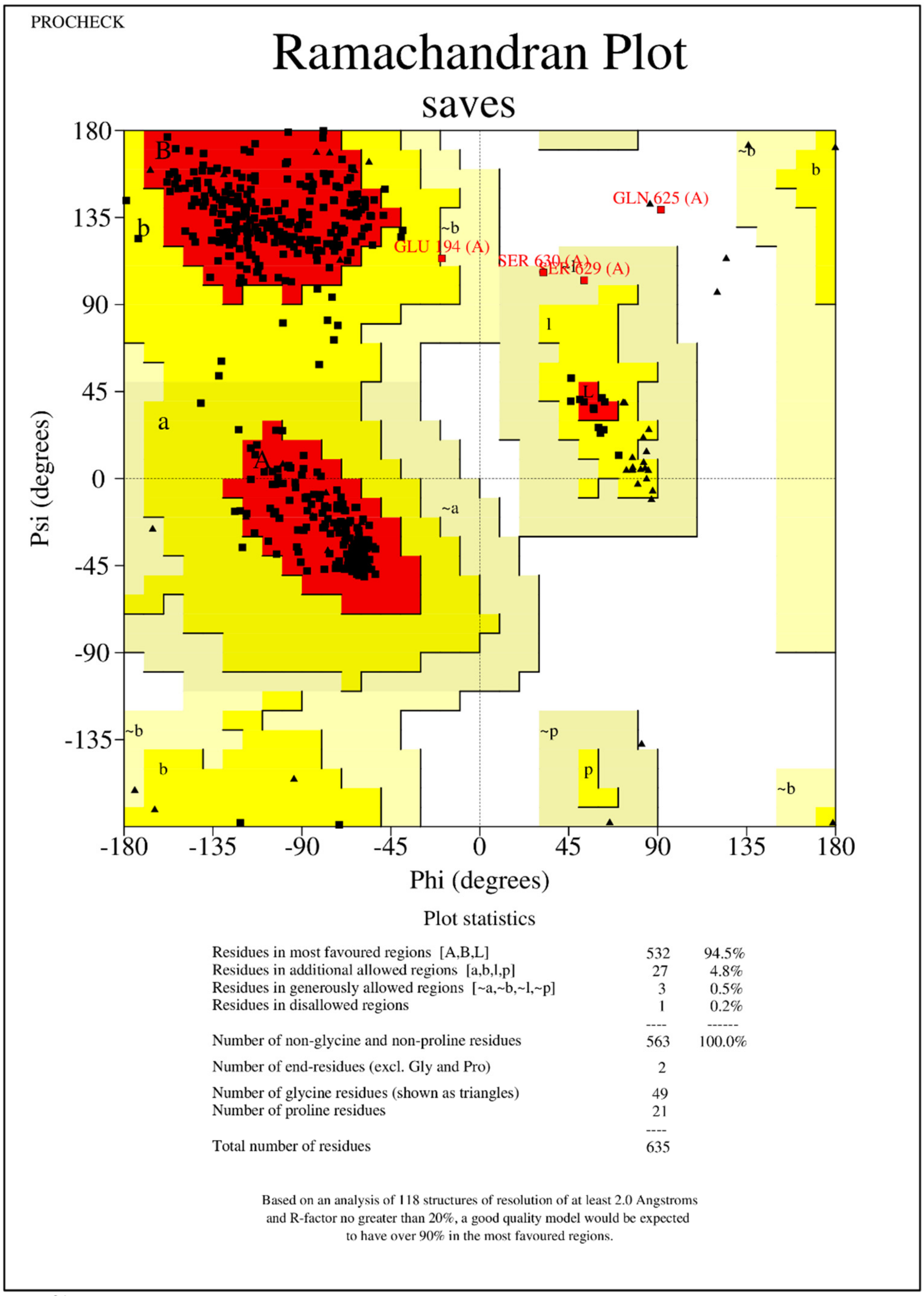

saves_01.ps

3. *hspa4:*

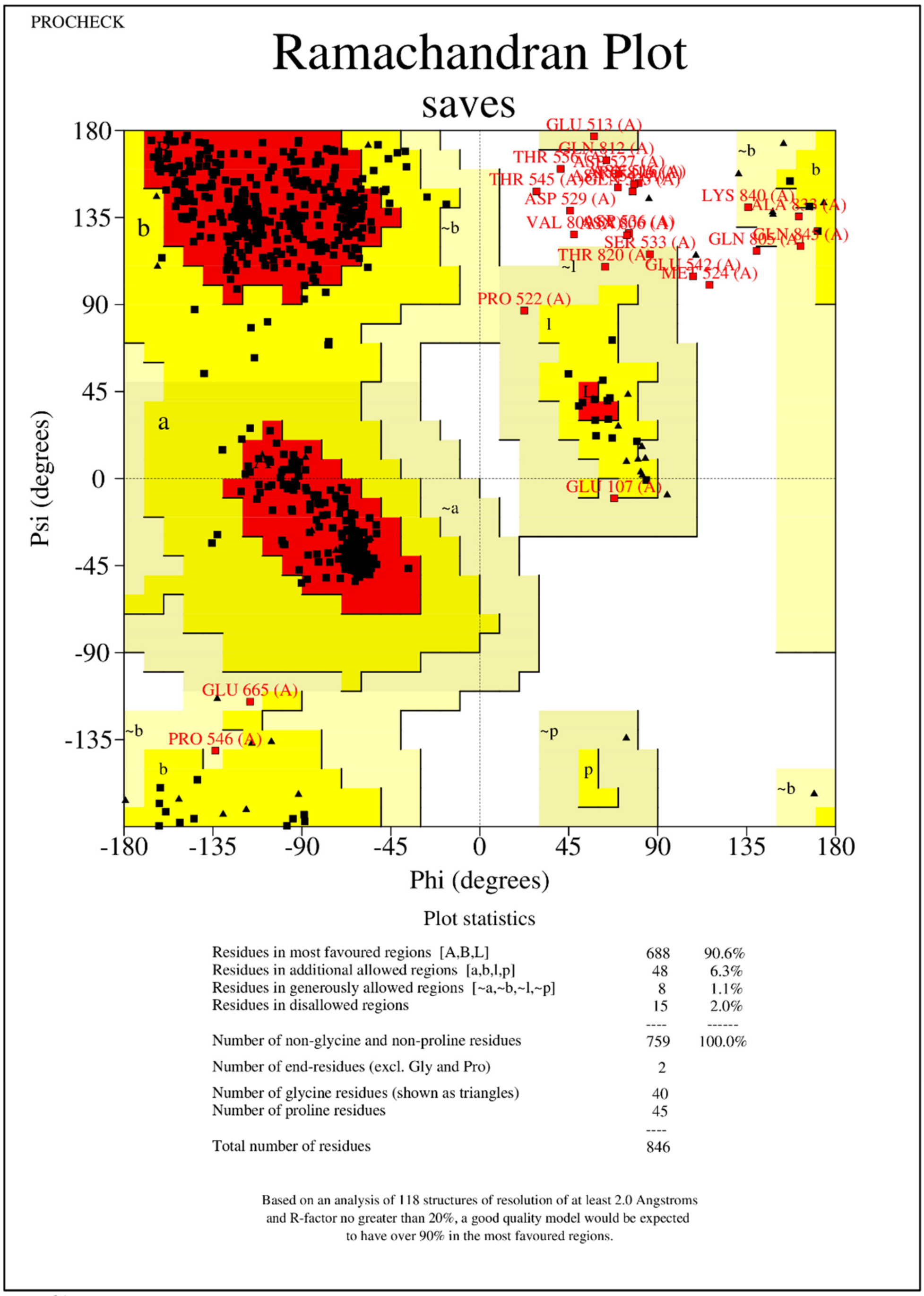

saves_01.ps

4. *hspa4a:*

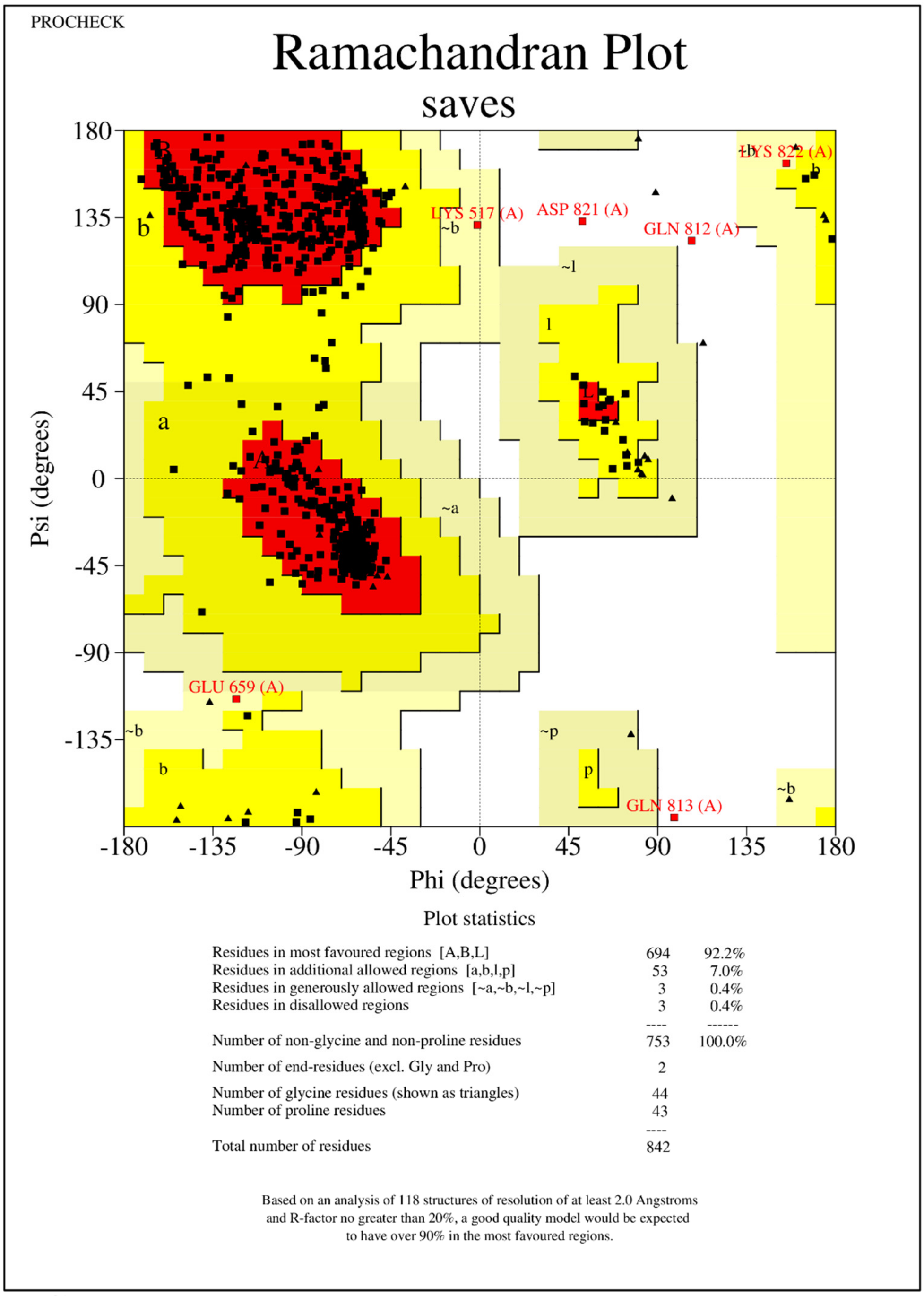

saves_01.ps

5. ***hspa4l:***

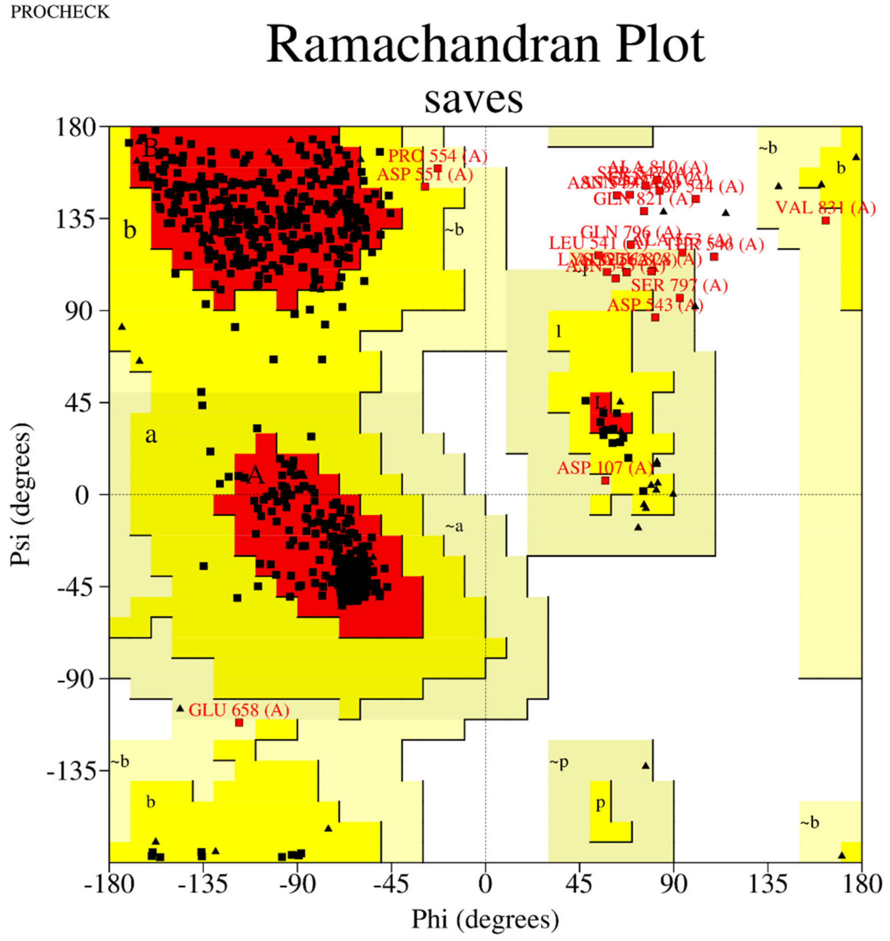

Plot statistics

Residues in most favoured regions [A,B,L]	682	91.9%
Residues in additional allowed regions [a,b,l,p]	39	5.3%
Residues in generously allowed regions [~a,~b,~l,~p]	12	1.6%
Residues in disallowed regions	9	1.2%
	----	------
Number of non-glycine and non-proline residues	742	100.0%
Number of end-residues (excl. Gly and Pro)	2	
Number of glycine residues (shown as triangles)	49	
Number of proline residues	39	

Total number of residues	832	

Based on an analysis of 118 structures of resolution of at least 2.0 Angstroms and R-factor no greater than 20%, a good quality model would be expected to have over 90% in the most favoured regions.

saves_01.ps

6. ***hspa5:***

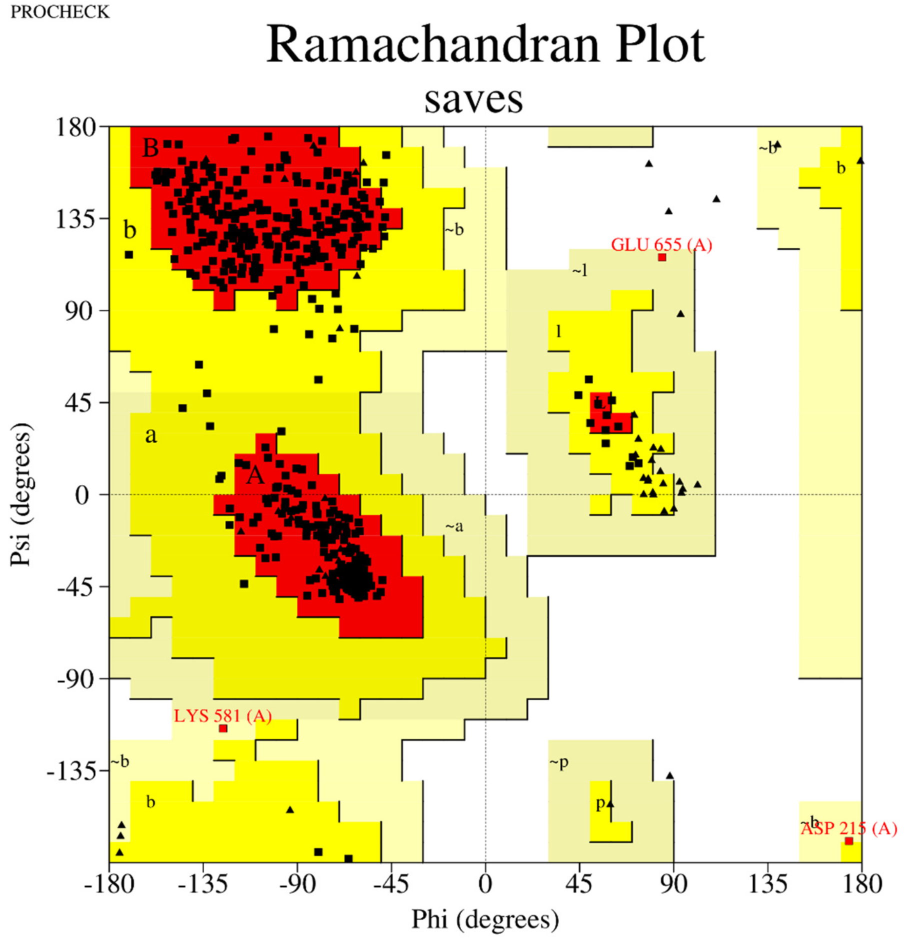

Plot statistics

Residues in most favoured regions [A,B,L]	538	93.6%
Residues in additional allowed regions [a,b,l,p]	34	5.9%
Residues in generously allowed regions [~a,~b,~l,~p]	3	0.5%
Residues in disallowed regions	0	0.0%
	----	------
Number of non-glycine and non-proline residues	575	100.0%
Number of end-residues (excl. Gly and Pro)	2	
Number of glycine residues (shown as triangles)	55	
Number of proline residues	24	

Total number of residues	656	

Based on an analysis of 118 structures of resolution of at least 2.0 Angstroms and R-factor no greater than 20%, a good quality model would be expected to have over 90% in the most favoured regions.

saves_01.ps

7. hspa8.1:

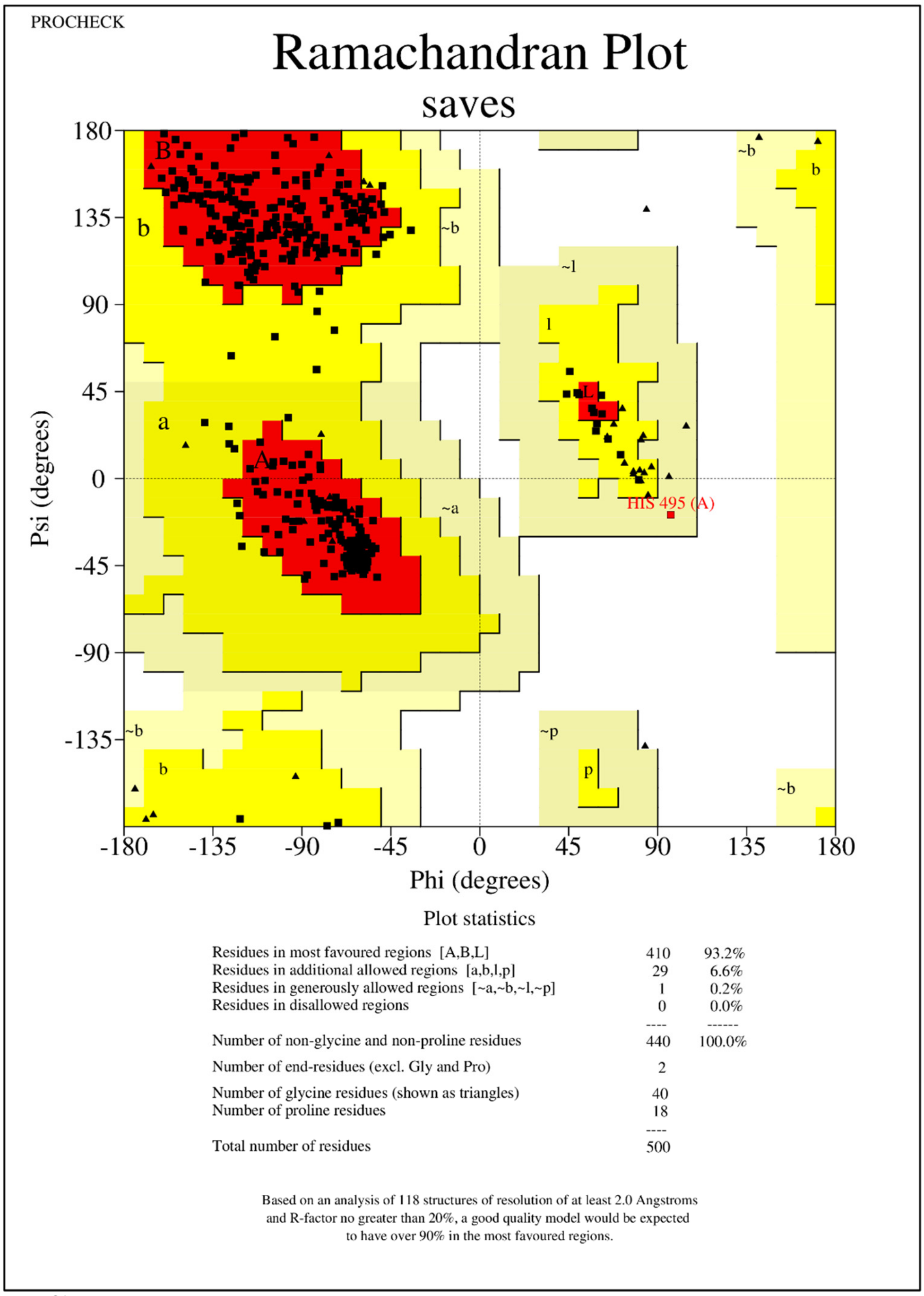

saves_01.ps

8. *hspa8.2:*

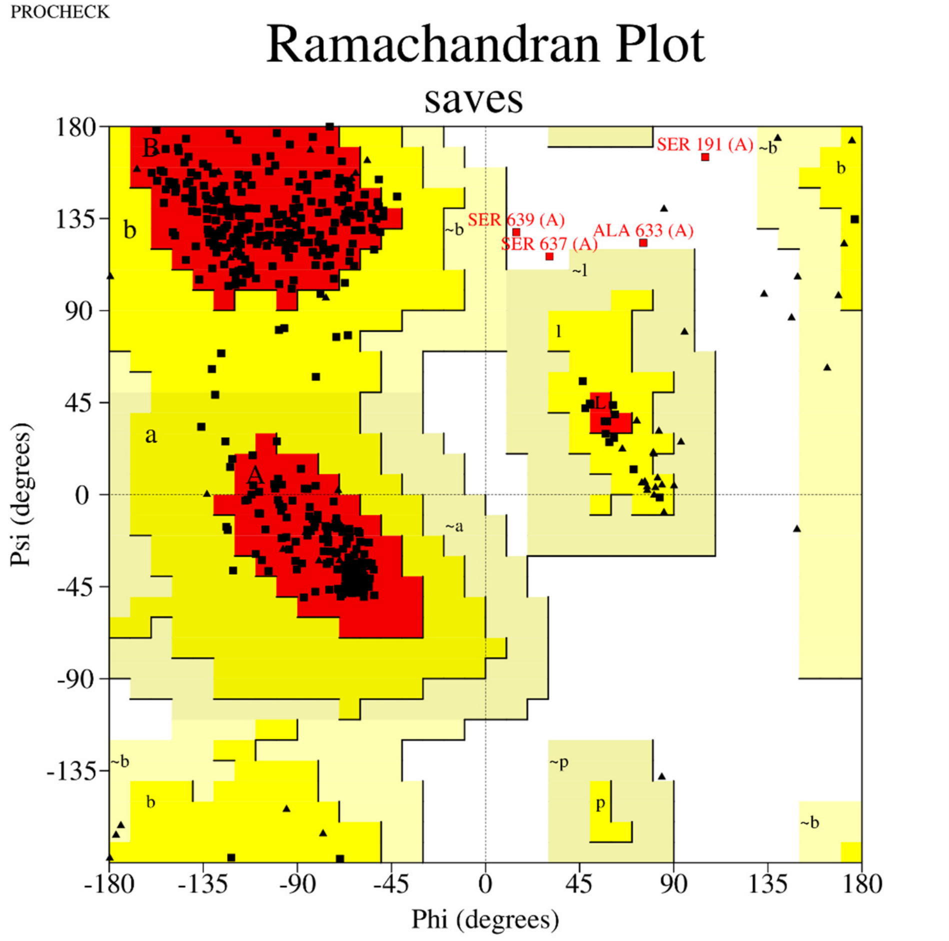

Plot statistics

Residues in most favoured regions [A,B,L]	530	93.8%
Residues in additional allowed regions [a,b,l,p]	31	5.5%
Residues in generously allowed regions [~a,~b,~l,~p]	0	0.0%
Residues in disallowed regions	4	0.7%
	----	------
Number of non-glycine and non-proline residues	565	100.0%
Number of end-residues (excl. Gly and Pro)	2	
Number of glycine residues (shown as triangles)	54	
Number of proline residues	26	

Total number of residues	647	

Based on an analysis of 118 structures of resolution of at least 2.0 Angstroms and R-factor no greater than 20%, a good quality model would be expected to have over 90% in the most favoured regions.

saves_01.ps

9. ***hspa9:***

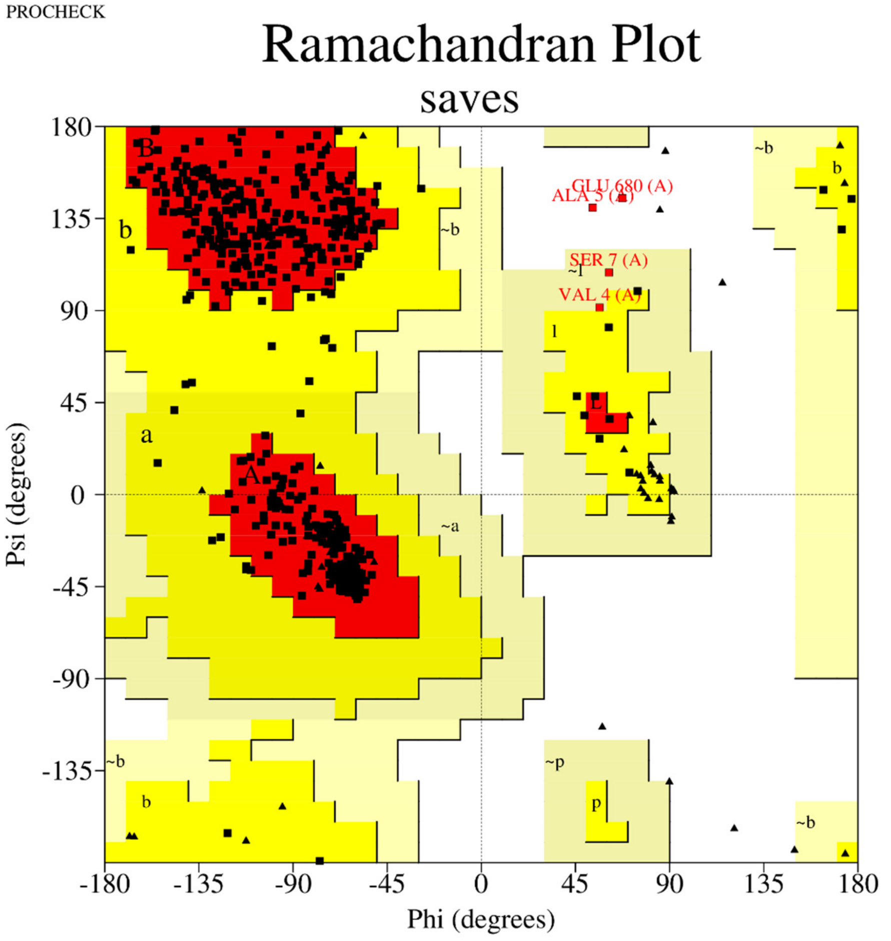

Plot statistics

Residues in most favoured regions [A,B,L]	565	92.9%
Residues in additional allowed regions [a,b,l,p]	39	6.4%
Residues in generously allowed regions [~a,~b,~l,~p]	2	0.3%
Residues in disallowed regions	2	0.3%
	----	------
Number of non-glycine and non-proline residues	608	100.0%
Number of end-residues (excl. Gly and Pro)	2	
Number of glycine residues (shown as triangles)	57	
Number of proline residues	21	

Total number of residues	688	

Based on an analysis of 118 structures of resolution of at least 2.0 Angstroms and R-factor no greater than 20%, a good quality model would be expected to have over 90% in the most favoured regions.

saves_01.ps

10. *hspa13:*

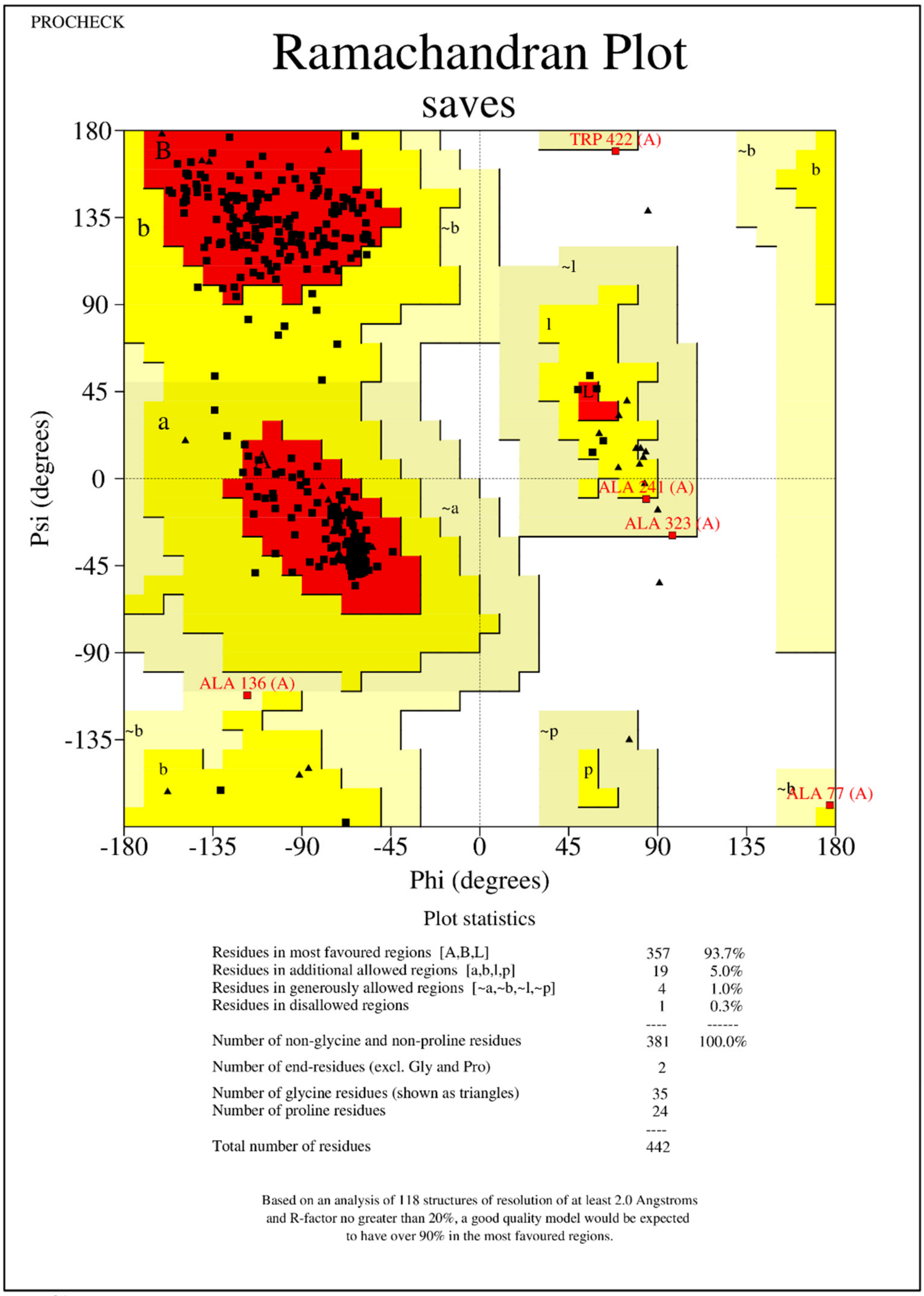

saves_01.ps

11. ***hspa14:***

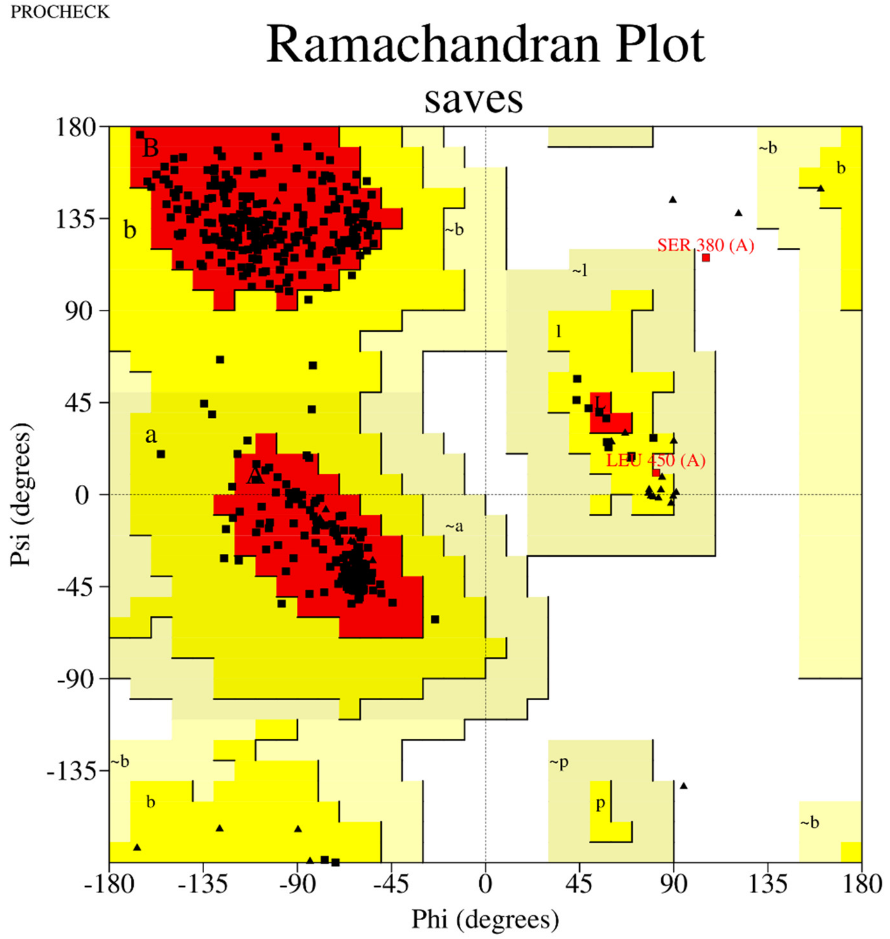

Plot statistics

Residues in most favoured regions [A,B,L]	421	93.6%
Residues in additional allowed regions [a,b,l,p]	27	6.0%
Residues in generously allowed regions [~a,~b,~l,~p]	1	0.2%
Residues in disallowed regions	1	0.2%
	----	------
Number of non-glycine and non-proline residues	450	100.0%
Number of end-residues (excl. Gly and Pro)	2	
Number of glycine residues (shown as triangles)	33	
Number of proline residues	20	

Total number of residues	505	

Based on an analysis of 118 structures of resolution of at least 2.0 Angstroms and R-factor no greater than 20%, a good quality model would be expected to have over 90% in the most favoured regions.

saves_01.ps

12. ***hyou1:***

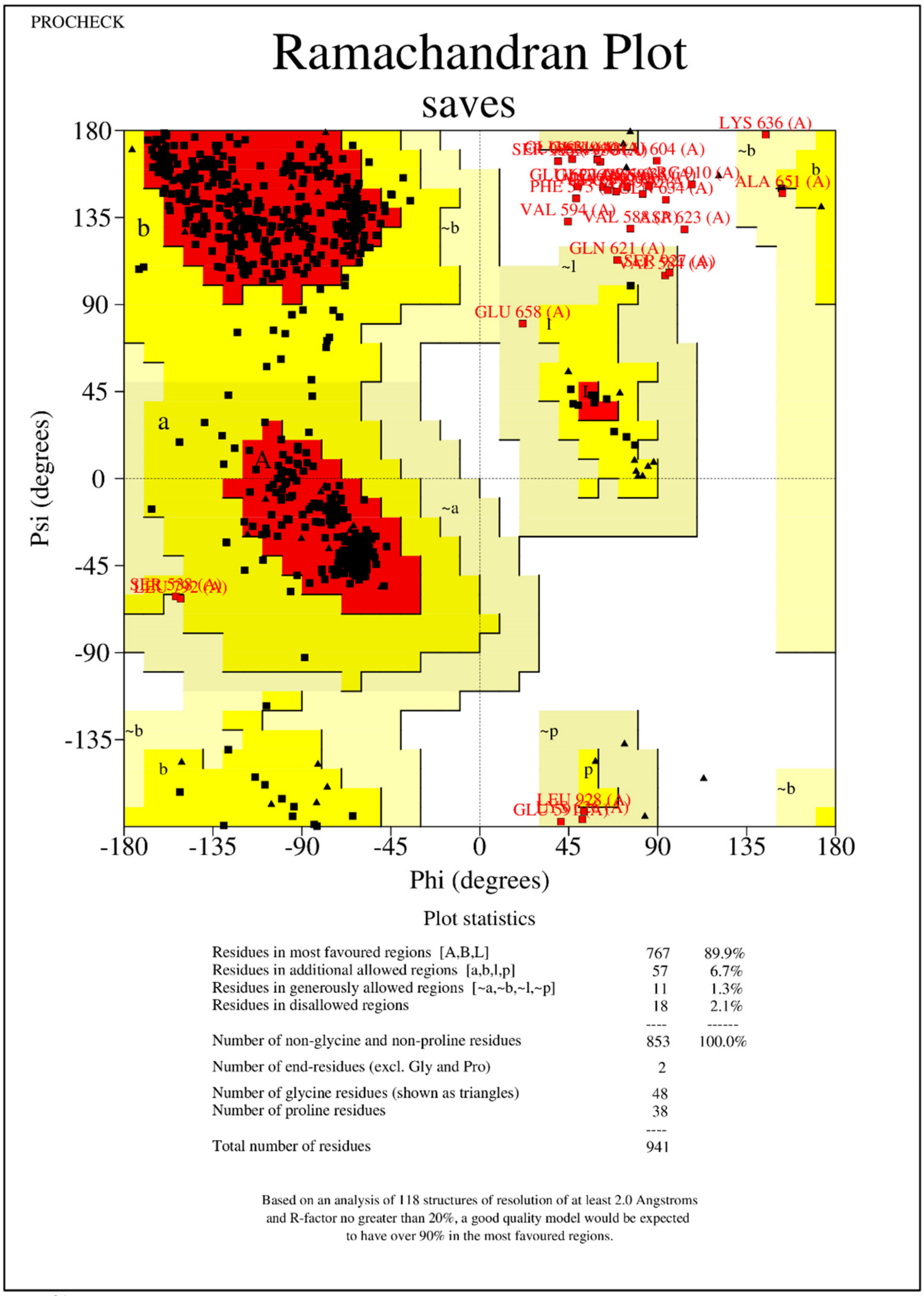

saves_01.ps

13. *hsp90aa1:*

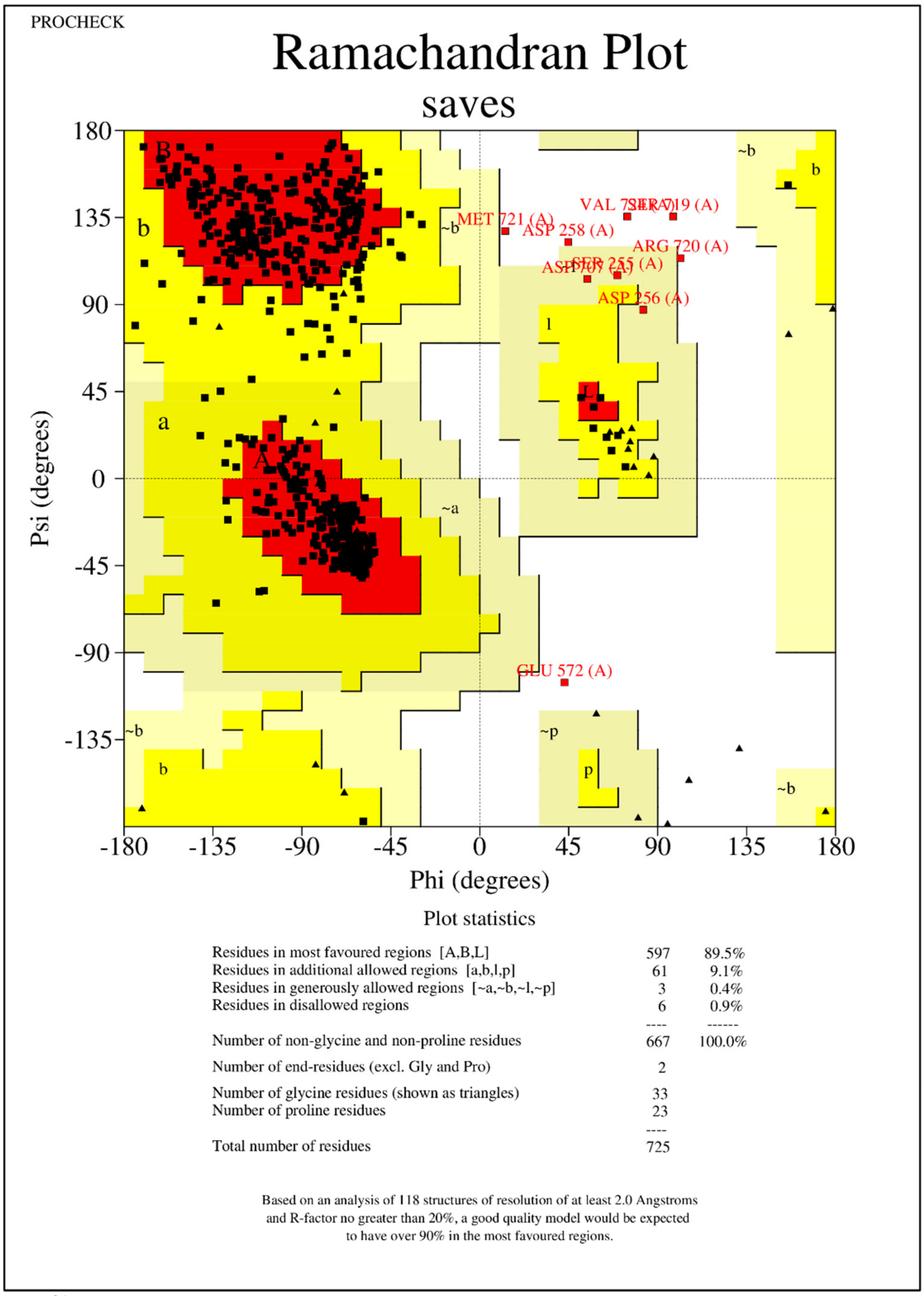

saves_01.ps

14. ***hsp90ab1***:

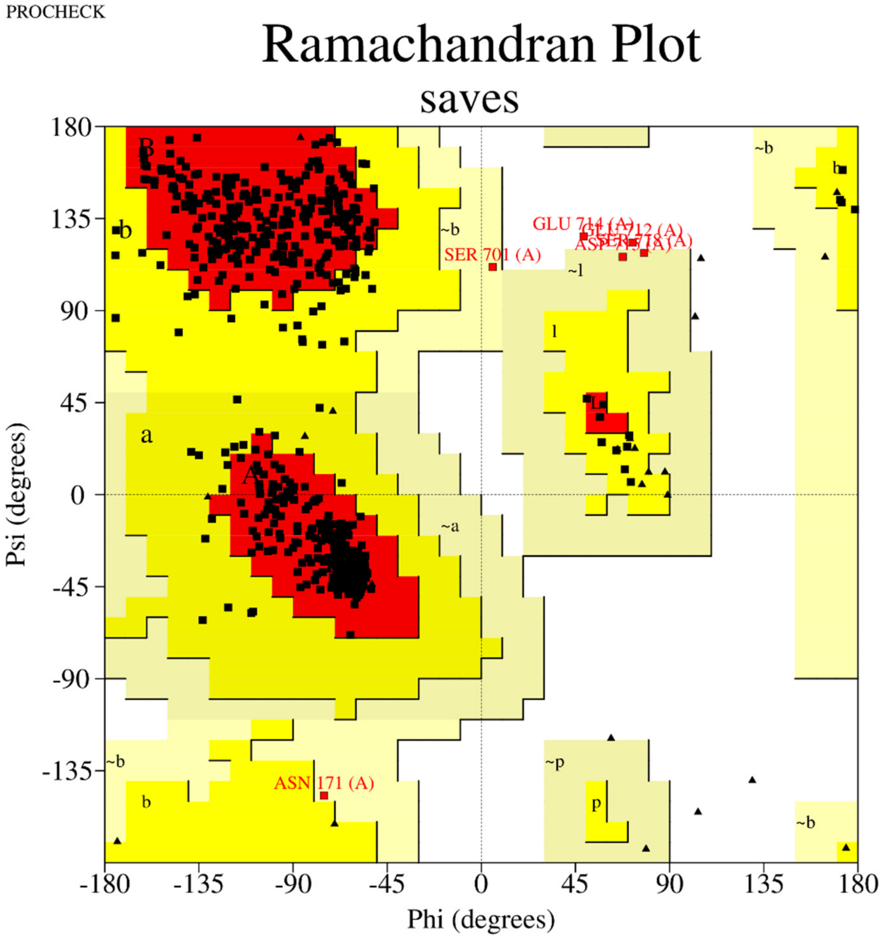

Plot statistics

Residues in most favoured regions [A,B,L]	605	90.4%
Residues in additional allowed regions [a,b,l,p]	58	8.7%
Residues in generously allowed regions [~a,~b,~l,~p]	4	0.6%
Residues in disallowed regions	2	0.3%
	----	------
Number of non-glycine and non-proline residues	669	100.0%
Number of end-residues (excl. Gly and Pro)	2	
Number of glycine residues (shown as triangles)	33	
Number of proline residues	20	

Total number of residues	724	

Based on an analysis of 118 structures of resolution of at least 2.0 Angstroms and R-factor no greater than 20%, a good quality model would be expected to have over 90% in the most favoured regions.

saves_01.ps

15. *hsp90b1:*

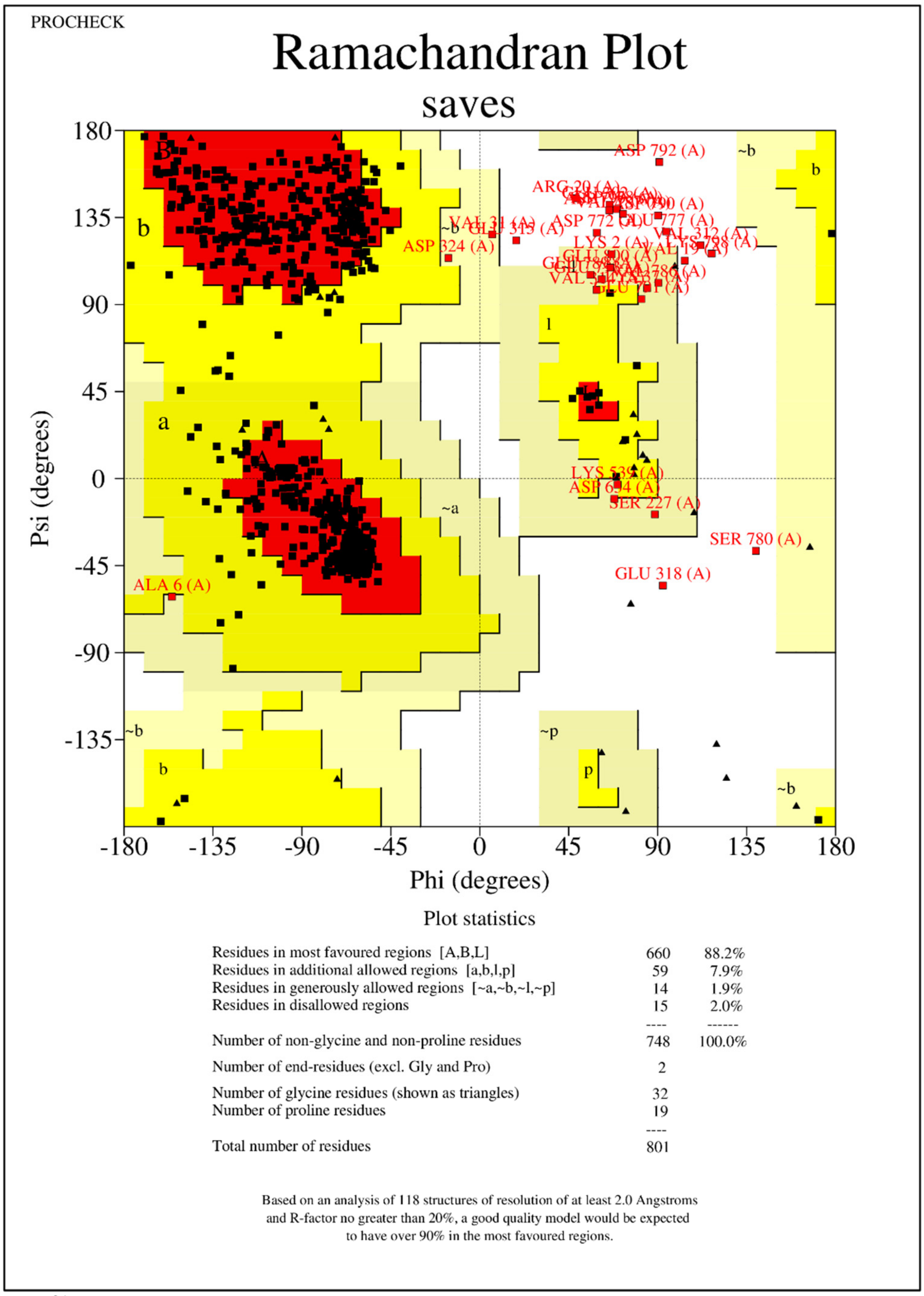

saves_01.ps

16. ***trap1:***

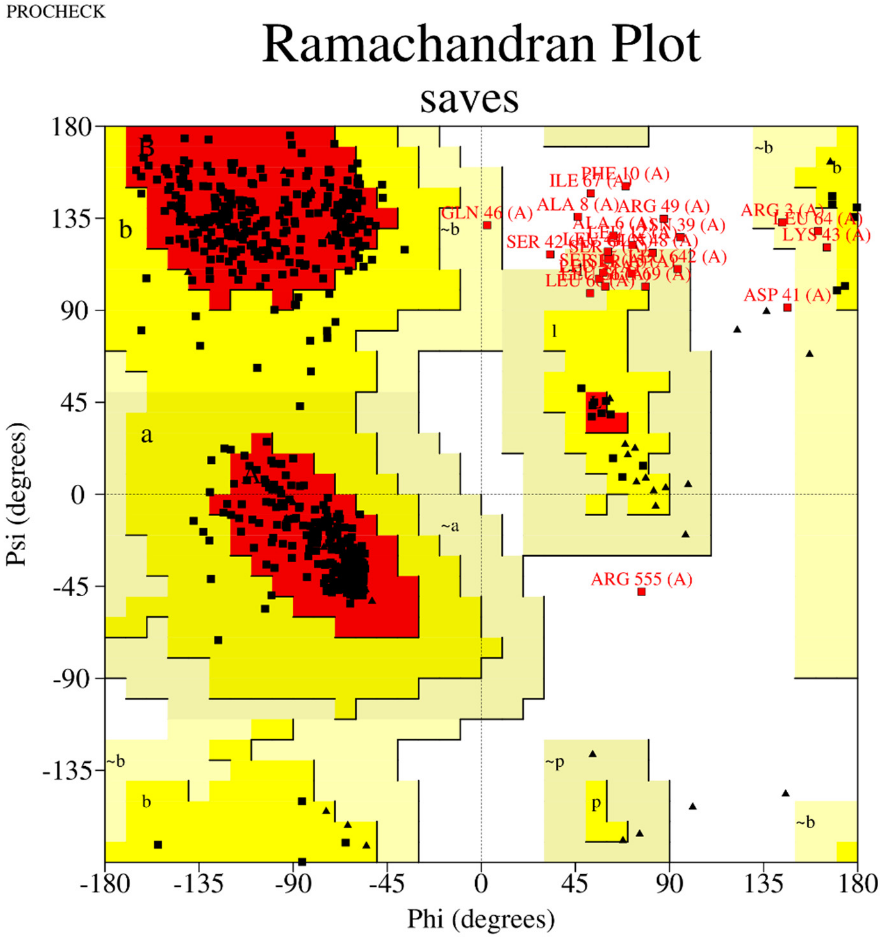

Plot statistics

Residues in most favoured regions [A,B,L]	580	88.8%
Residues in additional allowed regions [a,b,l,p]	49	7.5%
Residues in generously allowed regions [~a,~b,~l,~p]	14	2.1%
Residues in disallowed regions	10	1.5%
	----	------
Number of non-glycine and non-proline residues	653	100.0%
Number of end-residues (excl. Gly and Pro)	2	
Number of glycine residues (shown as triangles)	37	
Number of proline residues	27	

Total number of residues	719	

Based on an analysis of 118 structures of resolution of at least 2.0 Angstroms and R-factor no greater than 20%, a good quality model would be expected to have over 90% in the most favoured regions.

saves_01.ps
